# *Hypericum perforatum* L. Regulates Glutathione Redox Stress and Normalizes Ggt1/Anpep Signaling to Alleviate OVX-Induced Kidney Dysfunction

**DOI:** 10.3389/fphar.2021.628651

**Published:** 2021-04-26

**Authors:** Yan-Ru Liu, Ning-Juan Yang, Meng-Li Zhao, Zhi-Shu Tang, Jin-Ao Duan, Rui Zhou, Lin Chen, Jing Sun, Zhong-Xing Song, Jin-Hang Hu, Xin-Bo Shi

**Affiliations:** ^1^ Shaanxi Province Key Laboratory of New Drugs and Chinese Medicine Foundation Research, Shaanxi Collaborative Innovation Center Medicinal Resources Industrialization, Shaanxi University of Chinese Medicine, Xianyang, China; ^2^ Key Laboratory for High Technology Research of TCM Formulae and Jiangsu Collaborative Innovation Center of Chinese Medicinal Resources Industrialization, Nanjing University of Chinese Medicine, Nanjing, China

**Keywords:** *Hypericum perforatum* L., menopause, Glutathione redox stress, proteomics, metabolomics

## Abstract

Menopause and associated renal complications are linked to systemic redox stress, and the causal factors remain unclear. As the role of *Hypericum perforatum* L. (HPL) in menopause-induced kidney disease therapy is still ambiguous, we aim to explore the effects of HPL on systemic redox stress under ovariectomy (OVX)-induced kidney dysfunction conditions. Here, using combined proteomic and metabolomic approaches, we constructed a multi-scaled “HPL-disease-gene-metabolite” network to generate a therapeutic “big picture” that indicated an important link between glutathione redox stress and kidney impairment. HPL exhibited the potential to maintain cellular redox homeostasis by inhibiting gamma-glutamyltransferase 1 (Ggt1) overexpression, along with promoting the efflux of accumulated toxic amino acids and their metabolites. Moreover, HPL restored alanyl-aminopeptidase (Anpep) expression and metabolite shifts, promoting antioxidative metabolite processing, and recovery. These findings provide a comprehensive description of OVX-induced glutathione redox stress at multiple levels and support HPL therapy as an effective modulator in renal tissues to locally influence the glutathione metabolism pathway and subsequent redox homeostasis.

## Introduction

Women undergoing menopause are at an increased risk of ovarian dysfunction that will lead to systemic estrogen exhaustion and accelerate aging. This process is presumed to have led to the emergence of a number of complications, such as hot flashes (or vasomotor symptoms), chronic kidney disease (CKD), cardiovascular disease, and neurodegenerative disease (Schindler et al., 2009; Torres et al., 2017) (Williamson et al., 2019). Of those conditions, a common comorbidity in women with menopause is closely linked to the process of CKD development, which increases mortality (Kattah et al., 2018; Vellanki and Hou, 2018). Although endogenous estrogen plays a critical role in the protection of renal function, the initiation of hormone replacement therapy (HRT) is recommended to stop or reverse this decline. HRT increases the incidences of breast cancer, kidney stones, heart disease, blood clots, stroke and gallbladder disease in women (Carol Wilson, 2011; Lobo and Roger, 2016; Chlebowski et al., 2020). Therefore, these risks are important factors contributing to the decision of women to use supplementary therapy other than HRT after menopause.

The menopausal estrogen depletion-induced renal redox imbalance has been considered a primary factor that contributes to aging acceleration (van der Schouw and Grobbee, 2005; Ahmed and Ramesh, 2016). Thus, an increasing number of women are choosing antioxidant-rich herbal medicines to relieve menopausal symptoms and prolong the lifespan, with lower risks of side effects (Li et al., 2013; Uhrin et al., 2018; Wang et al., 2018). *Hypericum perforatum L* (HPL) has been used as an estrogenic botanical in the menopausal period to treat depression and anxiety, reduce vasomotor symptoms (e.g., hot flashes), modify reproductive-urinary system dysfunctions, rescue cognitive impairment, and balance lipid metabolism (Butterweck et al., 2003; Liu et al., 2015). The clinical significance of HPL was initially attributed to polyphenols, including naphthodianthrones (0.06–0.4%, hypericin, pseudohypericin, etc.), flavonoids (2–4%, hyperoside, quercitrin, rutin, etc.), and phloroglucinols (0.2–4%, hyperforin, adhyperforin, etc.) (Kennedy and Wightman, 2011). Notably, in the alleviation of reproductive-urinary system dysfunction, especially kidney dysfunction, flavonoids and their metabolites have been shown to be effective at treating kidney diseases. Rutin (∼1.6% in HPL) has been shown to reverse hyperglycaemia-induced renal endothelial dysfunction by reducing RhoA/ROCK activation and to prevent oxidative stress by mediating Nrf2 activation (Wang et al., 2016). Hyperoside (0.1–0.5% in HPL) directly inhibits adriamycin-induced mitochondrial dysfunction and podocyte injury through the inhibition of chondriokinesis *in vivo* and *in vitro*, thereby ameliorating renal and podocyte injury to reduce albuminuria in individuals with early diabetic nephropathy (Chen et al., 2017). In addition, hyperoside exhibits a novel therapeutic potential for diabetic nephropathy by attenuating AGE/RAGE binding and TGF-β1 expression through the inhibition of pERK1/2, p38 MAPK, and IκB phosphorylation (Zhang et al., 2013; Kim et al., 2016). Hyperoside also exerts a significant therapeutic effect on kidney stone formation by inhibiting the deposition of renal calcium oxalate crystals (Nirumand et al., 2018). Specifically, for renal oxidative stress-induced dysfunction, hyperoside prevents renal oxidative stress-induced glomerular basement membrane injury by reducing malondialdehyde, xanthine oxidase, superoxide dismutase (SOD), and catalase levels in mouse models of diabetes mellitus (Zhang et al., 2016; An et al., 2017). Although the majority of HPL-mediated effects on the modulation of redox homeostasis occur in the kidney, which may also facilitate resistance to oxidative stress and extend the lifespan, to date, the evidence of any effects of HPL on estrogen decline-induced renal oxidative stress or metabolic disorders during menopause is equivocal.

Here, we investigated whether an HPL treatment might reduce disturbances in the kidney microenvironment associated with fluctuating protein and metabolite levels following ovarian exhaustion. Using ovariectomized (OVX) rats as a model of systemic estrogen disruption, we performed global proteomics and metabolomics; the identified protein biomarkers were confirmed by performing molecular analyses. We then constructed a multivariate statistical analysis strategy to integrate proteomic and metabolomic data for the evaluation of the therapeutic efficacy in preventing cellular stress, which allowed the assignment of identified biomarkers to protein types within a menopausal animal model. In addition, we utilized these biomarkers to identify a novel HPL rescue function under conditions of ovarian failure-induced kidney abnormalities.

## Materials and Methods

### Chemicals, Antibodies, and Reagents

Optima LC grade acetonitrile was purchased from Merck (Merck, Darmstadt, Germany), formic acid was purchased from Fluka (Honeywell, Morris Plains, NJ, United States), and ultrapure water was prepared using a Milli-Q purification system (Merck Millipore, Darmstadt, Germany). Primary antibodies were purchased from Abcam (Cambridge, MA, United States): anti-ERα (ab3575), anti-ERβ (ab3576), anti-Ggt1 (ab109427), and anti-Anpep (CD13; ab108310). Metabolite standards were obtained from Sigma-Aldrich (Spruce St., St Louis, MO, United States).

### Preparation of HPL Extracts

Erial parts of *Hypericum perforatum* L (HPL) were collected on *Taibai Mountain*, Shaanxi, China, in September 2019 and identified by Prof. Yong-Gang Yan, and the specimens were deposited at Shaanxi University of Chinese Medicine, Shaanxi, China. The herbs were dried at room temperature and stored at 4 °C until use. According to the European Medicines Agency assessment report on HPL, lyophilized 65% ethanol extracts (5:1, 100 mg of extracts were equivalent to 500 mg of HPL) were standardized to 0.3% hypericin and contained 1.09% rutin, 0.65% hyperoside, 0.45% isoquercitin, 0.04% quercitrin, and 0.07% quercetin (the HPL flavonoid content determination is described in Supplementary method I, Supplementary Figure S1, and Supplementary Tables S1, S2).

### Animals and Treatment

**FIGURE 1:**
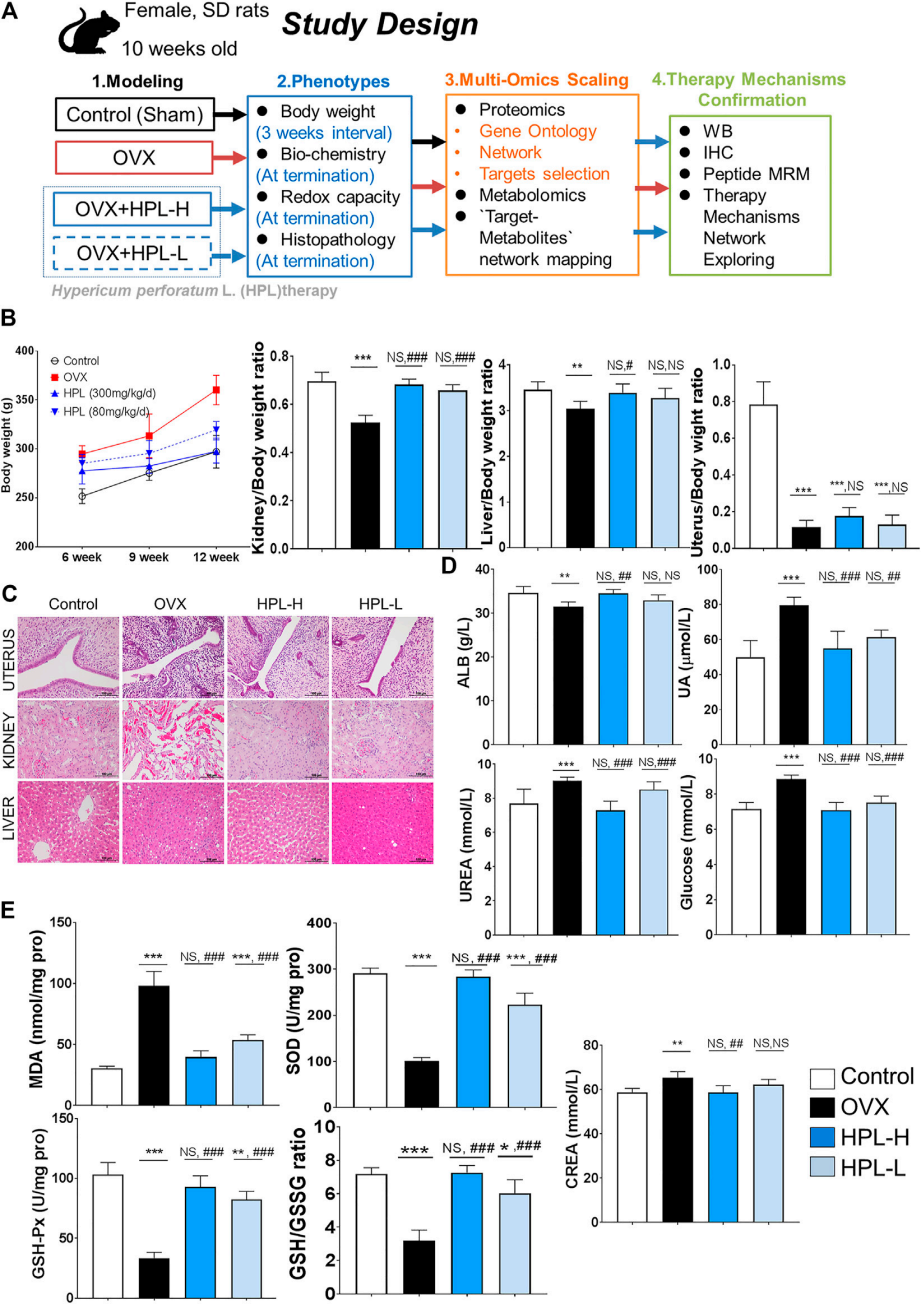
HPL therapy ameliorated pathophysiological and biochemical phenotypes of kidney dysfunction and redox stress in rats with OVX. **(A)** Study design. **(B)** Average weekly body weight (g) from 0 to 12 weeks before and after surgery and average organ/body weight ratios of the kidney, liver, spleen, and brain (*n* = 6). **(C)** Histological images of uterine, kidney, and liver tissues. Scale bar = 100 µm. **(D)** Renal biochemical profiles of serum levels of ALB, UA, urea, GLU, and CREA (*n* = 6). **(E)** Redox profiles of MDA, SOD, and GSH-Px levels, and the GSH/GSSG ratio in serum samples (*n* = 6). The data are pooled from three experiments (means ± *s*.d.). HPL therapy dosage: HPL-H, 300 mg/kg/d; HPL-L, 80 mg/kg/d. ^*^ *p* < 0.05, **p < 0.005, and ***p < 0.0005 compared with control rats; #*p* < 0.05, ##*p* < 0.005, and ###*p* < 0.0005 compared with OVX rats. NS, not significant.

Female Sprague-Dawley rats aged 8–10 weeks were obtained from the Fourth Military Medical University Animal Center (Certification No. SCXK (Shaanxi) 2018–001) and maintained in individual cages on a 12 h light/dark cycle at 23 ± 2°C and 55 ± 10% relative humidity. Food and drinking water were provided regularly and were unrestricted. Body weights were recorded. Rats were randomized and divided into four groups that were subjected to sham surgery (control), OVX, or assigned randomly to receive HPL ethanol extract therapy (HPL-H: OVX + HPL extract 300 mg/kg/d, HPL-L: OVX + HPL extract 80 mg/kg/d). Technical details of OVX and HPL therapy designs for all experiments are described in previous studies (Liu et al., 2014b; Liu et al., 2015). After a 7 day recovery from surgery, experimental sessions commenced using the protocol outlined in Figure 1A. Body weight was monitored weekly, while the organ-to-body weight ratio was determined at the end point. Animal handling and experimentation were carried out in accordance with the guidelines of the Guide for the Care and Use of Laboratory Animals and were approved by the Laboratory Animal Care and Use Committee of the Shaanxi University of Chinese Medicine.

### Urinary Sample Preparation for the Proteomic Analysis

Urine samples were collected at 24 h intervals in 10 ml epoxy resin tubes containing 0.5 ml of 0.1% sodium azide (Alfa Aesar, Ward Hill, MA) over gel ice packs (TECHNI ICE^™^, Frankston Victoria, Australia). For the proteomic analysis, 300 µL aliquots of urine were centrifuged twice at 10,000 × *g* for 20 min at 4°C to separate urinary proteins from small molecules using an Amicon Ultra 0.5 ml Centrifugal Filter (10 kDa, UFC501096, Merck Millipore, Darmstadt, Germany) according to the manufacturer’s instructions. The proteins captured in the filter were washed with water three times by centrifugation at 10,000 × *g* for 30 min at 4°C to remove possible interference. Then, the concentrated ultrafiltrate was centrifuged at 3,000 × *g* for 1 min and freeze-dried for protein determination. All urinary solutions were stored at −20 °C until use.

### Urinary Sample Preparation for the Metabolomic Analysis

For the metabolomic analysis, primary urine samples were prepared using published procedures (Liu et al., 2015). Urine samples were collected at 24 h intervals on 4°C gel ice packs (TECHNI ICE^™^, Frankston, Victoria, Australia) and processed by centrifugation at 12,000 × *g* for 10 min at 4°C (Sorvall Legend Micro 17R, Thermo Fischer Scientific, Inc., Waltham, MA, United States). Prior to the LC-MS analysis, each lyophilized sample was dissolved in a 0.1% formic acid-acetonitrile solution (95:5), and a 5 µL aliquot was used as the injected volume.

### Serum Sample Preparation

All rats were anesthetized with a 2% sodium phenobarbital solution without restraint at the end of the experiments. Blood was drawn from the inferior vena cava and allowed to clot, and then serum was collected by centrifugation at 14,000 × *g* for 10 min. For the metabolomic analysis, 100 μL of serum were mixed with 400 μL of extraction solvent (methanol:acetonitrile:acetone = 1:1:1, v/v). After the mixture was vortexed for 1 min and incubated at 4°C for 4 h, the mixed solution was centrifuged at 14,000 × *g* for 15 min at 4°C. Two hundred microliters of the supernatant from each mixture ere prefrozen at −80°C and dried using a laboratory freeze dryer (Vaco5, Zirbus Technology, Harz, Germany). Prior to the LC-MS analysis, dried samples stored at −80°C were dissolved in 100 μL of a 0.1% formic acid solution (water:acetonitrile = 1:1), and a 5 µL aliquot was used as the injected volume.

### Urinary Proteome Analysis

The total protein concentration was measured in each sample using a BCA Protein Assay Kit (Thermo Scientific) at an absorbance of 570 nm and referenced to a standard curve. Urinary proteins were fractionated by sodium dodecyl sulfate-polyacrylamide gel electrophoresis as described previously (Shevchenko et al., 2006).

#### iTRAQ Labeling and 2D-HPLC-MS/MS Analysis

Each 200 µg extract sample was prepared using standard digestion, reduction, and alkylation procedures prior to isobaric tag for relative and absolute quantitation (iTRAQ) labeling (Shevchenko et al., 2006). Peptide labeling (114–117) with the iTRAQ reagent was performed according to the manufacturer’s protocol (Unwin et al., 2010). The four isotopic iTRAQ-labelled samples were combined and fractionated on a Waters ultra-performance LC instrument using a C18 column (Waters BEH C_18_ 2.1 × 50 mm, 1.7 μm). Peptides were eluted at a flow rate of 600 ml/min with a linear gradient of 5–35% solvent B (acetonitrile) over 10 min; solvent A was 20 mM ammonium formate with the pH adjusted to 10. The absorbance was monitored at 214 nm, and 10 fractions were collected. The fractions were separated by nano-high-performance LC (Eksigent Technologies, Dublin, CA, United States) on a secondary RP analytical column (Eksigent, C18, 3 μm, 150 mm × 75 µm) using a gradient mobile phase of 98% acetonitrile, 2% Milli-Q water (A) and 0.1% formic acid (B) at a flow rate of 0.3 μL/min. The gradient program was as follows: 0–5 min: 5% B, 6–65 min: 5–40% B, 65–66 min: 40–80% B, 66–71 min: 80% B, 71–72 min: 80–5% B, and 72–90 min: 5% B. Full scan and MS/MS experiments were performed in duplicate on an AB SCIEX TripleTOF 5,600 system (Framingham, MA, United States). Information-dependent data acquisition mode was used to switch automatically between MS and MS/MS acquisition with the following parameters: an ion spray voltage of 2,500 V, curtain gas (CUR) of 25 p. s.i., cycle time of 1 s, and dwell time of 30 ms. MS spectra were acquired between 350–1,250 m/z (molecular weight/valency) in high-resolution mode (40,000 resolution for full scans and 20,000 resolution for MS/MS scans) with a rolling collision energy using a 250 ms accumulation time/spectrum and 50 mDa mass tolerance, and the 10 most intense precursors per cycle were selected for fragmentation with a dynamic exclusion time of 30 s. Data were acquired using Analyst 2.0 software (AB Sciex). The collected samples were resuspended in a 0.1% formic acid solution (0.1% formic acid, 2% acetonitrile, and 98% Milli-Q water) and analyzed on an Eksigent C18 column (150 mm × 75 μm, 3 µm) using a gradient mobile phase of 98% acetonitrile, 2% Milli-Q water (A) and 0.1% formic acid (B) at a flow rate of 0.3 μL/min. The gradient program was as follows: 0–5 min: 5% B, 6–65 min: 5–40% B, 65–66 min: 40–80% B, 66–71 min: 80% B, 71–72 min: 80–5% B, and 72–90 min: 5% B.

#### Urinary Proteomic Data Processing

Original data (*.wiff and *.wiff.scan) acquired from the MS/MS analysis in each LC run were converted to Mascot generic files (*.mgf) using ProteinPilot 4.5 software (AB Sciex). The MS/MS peak lists of the formatted data were then searched with Mascot (Matrix Science, London, United Kingdom; version 2.3.02); spectra were searched against the UniProt database, and the taxonomy was set to rat. The following search parameters were adopted: search type, MS/MS ion search; enzyme, trypsin; fragment mass tolerance, ± 0.1 Da; mass values, monoisotopic; variable modifications, oxidation (M), and iTRAQ 4 plex (Y); peptide mass tolerance, 25.0 ppm; instrument type, ESI-QUAD-TOF; one missed cleavage site was allowed, and the carbamidomethylation of cysteine, iTRAQ 4 plex of lysine, and the N-terminus were set as fixed modifications.

Scaffold (version Scaffold_4.4.5, Proteome Software Inc., Portland, OR, United States) was used to validate MS/MS-based peptide and protein identifications. Peptide identifications were accepted if they achieved an FDR <1.0% using the scaffold local FDR algorithm. Protein identifications were accepted if they achieved an FDR <1.0% and contained at least one identified peptide.

#### Gene Ontology Annotation and Network Analyses

The protein annotation was subjected to a GO analysis based on the biological responses or disease analysis for biological processes, molecular functions, and cellular components. The differentially expressed proteome (>1.5-fold changes in all ratios) from the iTRAQ LC-MS/MS analysis of urinary samples was interpreted using Ingenuity Pathway Analysis (IPA) software 9.1 (Ingenuity Systems, Mountain View, CA, United States, www.ingenuity.com). Both upregulated and downregulated proteins were computed in accordance with each network to conduct a pathway analysis and identify proteins associated with the pathways of interest (Ashburner et al., 2000; Harris et al., 2004).

We additionally used the hypothesis strategy GeneMANIA (freely available at http://genemania.org) to generate hypotheses regarding gene functions, calculate gene lists, and weight genes, the algorithm of which is designed to automatically weight networks based on the relevance to the gene set. We performed multiple gene queries to identify the most closely connected genes among the networks and attributes selected from IPA enrichment and to evaluate the significance of selected proteins from the observed network. Physical interactions, pathways, and genetic interactions were generated, and datasets relevant to ovarian failure and the prosurvival signaling network were collected (Vlasblom et al., 2015; Pirooznia et al., 2016; Franz et al., 2018).

#### LC-MS Scheduled MRM Analysis for Target Protein Determination

We next performed an LC-MS MRM scan of the isotopically labeled peptides for relative quantitation of these selected proteins to further determine the functions of the selected proteins in the targeted signaling pathways. Screening the specific peptides representing each target protein is a critical step in MRM quantification. Therefore, we first identified the target proteins corresponding to peptides from the established targeted peptide spectral library (Chen et al., 2014; Yunee et al., 2016). Next, the screened peptides were synthesized using solid-phase peptide synthesis technology and Skyline software (version 2.1.0) to calculate the m/z of the divalent ions of each peptide and the m/z of the fragment ions of each divalent ion. Collision energy optimization was performed for these candidate fragment ions, which ranged from 400 to 1,000 m/z, and the top five highly sensitive precursor-product ions were then selected (the detailed optimization information for each protein is listed in Supplementary Tables S3). When the MRM method was successfully developed, each batch of peptides was obtained from 200 µg of protein following trypsin in-gel digestion, purification, and freeze-drying (Chen et al., 2014; Yunee et al., 2016). One hundred microliters of the peptide solution in acetonitrile from each sample was spiked with 2 pmol/μL of heavy peptide standards; these samples were combined into five batches. Then, 4 µL of the mixed sample were loaded onto an Eksigent C_18_ column (250 mm × 75 μm, 3 µm) and analyzed with an Eksigent NanoLC-1D Plus system. The mobile phase consisted of 0.1% formic acid in acetonitrile (A) and 0.1% formic acid in water (B). The gradient program was as follows: 0–45 min: 5–30% B, 45–50 min: linear gradient to 80% B, 50–52 min: 80% B, 52–53 min: linear gradient to 5% B, and hold for 7.0 min, with a flow rate of 300 nL/min. The column oven temperature was maintained at 30°C. The LC system was coupled with a 6,500 triple-quadruple mass spectrometer LC/MS system (AB Sciex) equipped with an electrospray ion source. Targeted acquisition of eluting ions was performed in MRM mode with the following ion transitions: Q1 and Q3 ions at a full width at half maximum resolution of 0.7 m/z, an ion spray voltage of 2,500 V, CUR of 25 p. s.i, cycle time of 1 s, dwell time of 30 ms, and a 5 ms pause between MS; the declustering potential value was predicted using Skyline software. Skyline software was used for peak visualization and inspection. Each sample mixed with light (endogenous) and heavy (synthetic) peptides was quantified by integrating the quantifier fragment ions to derive a light-to-heavy peptide ratio.

### LC-MS for Metabolite Determination

According to our targeted metabolomic experiment, potential metabolite markers were quantitated by comparing the areas of the peaks corresponding to the unlabelled compounds to the areas of the peaks for the external standards (method validation is shown in Supplementary Method II, Supplementary Tables S4, S5, and Supplementary Figure S2) (Liu et al., 2015).

### Pathway Enrichment Analysis of the HPL Treatment

We performed an integrated metabolic pathway analysis of results obtained from combined metabolomics and proteomics datasets conducted to explore the effect of HPL. We selected the “joint pathway analysis” module from the MetaboAnalyst platform (http://www.metaboanalyst.ca), which aims to represent whether the HPL-induced proteins and metabolites in a particular pathway are significantly enriched (Chong et al., 2018). After the datasets of proteins (corresponding gene id) and metabolites were input, the “all pathways (integrated)” integration method, hypergeometric test, degree centrality and combined *p* values (unweighted) were chosen to evaluate the role of selected proteins or metabolites.

### Body Weight Observation and Histopathological Analysis

A complete histopathological analysis was performed on all OVX rats 3 months after surgery. For the histological analysis, samples of the uterus, kidney, and liver were collected 12 weeks after surgery. Briefly, a gross evaluation was conducted when possible, tissues were infiltrated with paraffin, and hematoxylin-and eosin (H&E) staining was performed. Sections were evaluated by two pathologists who were blinded to the experimental groups. Histopathological changes were diagnosed using the histological classifications for rats.

### Serum Biochemical Assays

Biochemical assays were evaluated 12 weeks after rats underwent OVX. Enzymatic assays for the detection of serum uric acid (UA), urea, glucose (GLU), creatinine (CREA), and albumin (ALB) levels were performed with commercial biochemical kits (MINDRAY Medical International Co., Ltd., Shenzhen, China).

### Renal Antioxidant Activity Evaluation

Renal activities of the antioxidants MDA, SOD, GSH-Px, GSH, and GSSG were measured. The assays were performed using a test kit according to the manufacturer’s instructions (Nanjing Jiancheng Bioengineering Institute, Jiangsu, China). A 10% kidney homogenate was prepared, and SOD, GSH/GSSG, GSH, GSH-Px, and MDA activities were determined according to the instructions provided with the kit.

### Immunohistochemistry Analysis

For the IHC analysis, rat tissues were fixed with 4% paraformaldehyde and embedded in paraffin. Then, 4 μm sections were generated, and IHC staining was performed using antibodies against the following antigens: ERα (ab3575, Abcam, Cambridge, MA, United States), ERβ (Abcam, ab3576), Ggt1 (Abcam, ab175384), and Anpep/CD13 (Abcam, ab108310). Tissue sections (3 mm thick) obtained from each group were treated as described below. The sections were deparaffinized, rehydrated, immersed in Tris-EDTA buffer (pH 9.0), and heated in an autoclave at 120°C for 3 min for antigen exposure. After washing and quenching, slides were blocked with buffer (1% bovine serum albumin (BSA) and 2.5% normal goat serum in TBS-T) for 20 min. Prior to counterstaining and mounting, the slides were incubated with the primary antibodies at 4°C overnight and then rinsed with phosphate-buffered saline (PBS) three times for five min each. On the next day, sections were incubated with Polymer Helper-conjugated secondary antibodies for 20 min in a 37°C incubator and then rinsed with PBS three times for 5 min each. Next, the sections were incubated with horseradish peroxidase-conjugated anti-rabbit antibodies (Alpha Diagnostic, San Antonio, TX, United States) for 20 min in a 37°C incubator and then rinsed with PBS three times for 5 min each. Staining was visualized using 3,3-diaminobenzidine (DAB) according to the manufacturer’s protocol (Vector Labs). The stained sections were scanned and qualified using the Nano Zoomer S210 Digital slide scanner (Hamamatsu Photonics, Shizuoka Pref., Japan) coupled with the digital slide viewer NDP. scan (version 3.2.12, Hamamatsu Photonics, Shizuoka Pref., Japan), and the distribution of staining in the membrane or cytoplasm was also recorded and assessed. DAB staining, as indicated by the brown color in immunohistochemistry analysis, was interpreted as positive expression. Image-Pro Plus 6.0 software was used to measure the area of IHC DAB staining to obtain the integrated optical density (iod) and staining area (area) values. The AOD ratio indicates the ratio of iod to area, and a larger AOD ratio represents a higher protein expression level.

### Western Blot Assay

We next confirmed whether the HPL exerted restorative effects. The uterine and kidney tissues were removed from experimental rats and subjected to western blot analysis. According to the standard procedure, 30 mg tissue samples were lyzed with 500 µL of cold RIPA lysis buffer, lysates were collected, and the total protein content was estimated using a BCA protein assay kit (Sigma, St. Louis, MO). After adjusting the protein concentration using RIPA buffer, an equal amount of total protein (20 µg) was resolved on 5% acrylamide gels (Amresco), and proteins were electrophoretically separated on SDS gels (10–15%, Sigma) at a constant voltage of 120 V for 20 min (*n* = 6 per group). Stain-free gels were then wet-transferred onto polyvinylidene difluoride (PVDF, Millipore) membranes at 300 mV constantly for 1 h, and equal transfer was routinely confirmed by Ponceau S staining. After blocking with 5% BSA-TBST (Sigma), the membranes were incubated with the following primary antibodies from Abcam at 4°C overnight: ERα (1:1,000, ab3575), ERβ (1:1,000, ab3576), Ggt1 (1:1,000, ab175384), and Anpep (1:1,000, ab108310). Blots were then washed and incubated with the appropriate secondary antibody for 40 min at room temperature. Protein bands were visualized using chemiluminescence (ECL, WBKLS0500, Millipore). Relative band pixel intensities were semiquantitatively analyzed using ImageJ software (version 1.8.0, National Institutes of Health, Bethesda, MD, United States), and protein loading was normalized using an antibody against glyceraldehyde 3-phosphate dehydrogenase (GAPDH) (sc-32233, Santa Cruz Biotechnology, Santa Cruz, CA).

### Quantitative Reverse Transcriptase Polymerase Chain Reaction Analysis

One hundred milligrams of kidney tissue were homogenized in TRIzol reagent (Servicebio Technology Co., Ltd., Wuhan, China) to extract total RNA, and 1 mg of RNA was reverse transcribed using a RevertAid First Strand cDNA Synthesis Kit (#K1622, Thermo Fischer Scientific). qRT-PCR was performed in a StepOnePlus^™^ Real-Time PCR System (Applied Biosystems, Foster City, CA, United States) using FastStart universal SYBR Green Master reagent (ROX, Roche, Basel, Switzerland). The oligonucleotides (Invitrogen, Carlsbad, CA, United States) for PCR were as follows: rat Ggt1 (ref. NM_053840.2, 154 bp, 60°C, Servicebio): forward primer: 5′- TTC​ACC​ATC​TAC​AAC​AGC​ACC​AC -3′ and reverse primer: 5′- CAT​AGC​CAC​GGA​TTT​CAC​CAG -3’; rat Anpep (ref. NM_031012.1, 264 bp, 60°C, Servicebio): forward primer: 5′- CCG​TCT​CAC​CCA​ATA​GAG​TCC​A -3′ and reverse primer: 5′- CCT​TGT​TGC​TAA​TGG​AGG​AGG​A -3’. Renal levels of the Ggt1 and Anpep mRNAs were normalized to the GAPDH mRNA and determined using the 2^−ΔΔCt^ method (Livak and Schmittgen, 2001).

### Statistical Analysis

The statistical analysis was conducted using one-way ANOVA followed by Sidak’s multiple comparisons test. GraphPad Prism 7.0 software (GraphPad Software Inc., San Diego, CA, United States) was used for the statistical analysis and data presentation. Differences with *p* values < 0.05 were considered significant. Statistical parameters are provided in the figure legends.

## Results

### HPL Relives the Perturbed Renal Phenotype Induced by OVX-Mediated Estrogen Depletion

The experimental protocol performed following OVX surgery is shown in Figure 1A. We examined the body weights and organic pathological changes at 3 months intervals. After 12 weeks of HPL treatment (both dosages), the body weight and kidney/body weight ratio reached values similar to those observed in control rats (Figure 1B). Consistent with previous reports, the body weight and organ ratios of the uterus, liver, and kidney exhibited trends toward recovery, indicating that HPL restored function following organ degeneration (Liu et al., 2015). A histological examination was conducted after 3 months of treatment to determine whether HPL relieved organ dysfunction. Notably, HPL treatment improved inflammatory degenerative and hemorrhagic lesions in the renal tubules in a dose-dependent manner. Surprisingly, HPL administration to OVX rats improved neither the uterine cavity nor endometrial gland status (Figure 1C).

### HPL Restores Kidney Homeostasis and Rescues the Glutathione Redox Balance

We next investigated the effect of the HPL treatment on serum profiles in OVX rats. HPL significantly reversed the abnormal kidney profiles in OVX rats in a concentration-dependent manner, including urea, glucose, and creatinine levels, to the levels detected in control rats. These data revealed the relationship between estrogen depletion and renal dysfunction and indicated that HPL minimized the risk of kidney disease in OVX rats (Figure 1D). Compared to the control group, the HPL group exhibited increasing trends in renal malondialdehyde (MDA) levels, along with the restoration of the decreasing trend of renal SOD levels, glutathione peroxidase (GSH-Px) levels, and the oxidized glutathione (GSH/GSSG) ratio in a dose-dependent manner (Figure 1E). Based on these findings, HPL functioned to remove the products of oxidative stress by increasing the activities of the antioxidant enzymes SOD and GSH-Px and the GSH/GSSG ratio, thereby balancing the distorted renal and redox conditions of rats with ovarian failure.

### HPL Administration Restores the OVX-Induced Abnormal Fluctuations in the Renal Proteomic Profile

**FIGURE 2:**
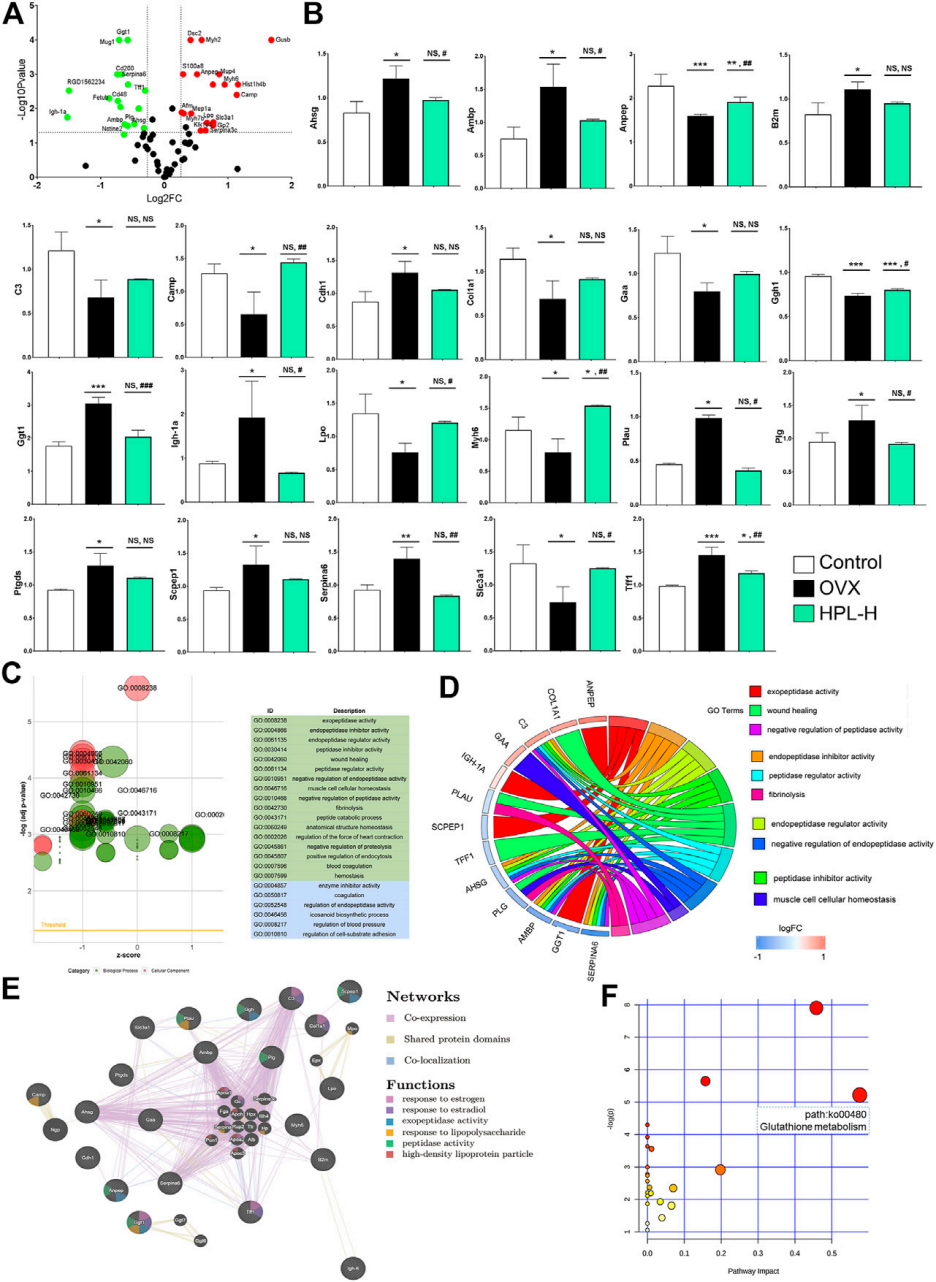
HPL therapy rescues the overall renal proteomic dysfunction induced by OVX. **(A)** Volcano plot of differentially expressed proteins identified in rats treated with HPL (HPL-H, 300 mg/kg/d). The *X*-axis shows the 117/116 ratio of proteins, reflecting the ratio of expression levels in HPL-treated rats to OVX rats, while the *Y* axis represents the *p* value of the repetition test, and each point in the plot represents one protein ratio. Red represents upregulated proteins, and green represents downregulated proteins. **(B)** Levels of the 21 screened proteins after HPL treatment. **(C)** GO bubble plot illustrating the significant enrichment of molecular functions and biological processes (adjusted *p*-value < 0.05) and the *z*-score of the term. The *X*-axis shows *z*-scores, and the *Y*-axis shows adjusted *p* values. Different colors represent three major categories: green indicates biological processes (BPs) and blue indicates molecular functions (MFs). Each bubble represents one GO term, and the bubble size represents the number of regulated genes. **(D)** GO Chord plot illustrating the gene annotation enrichment relationships and representative significant GO terms that are distinctly classified into species-specific gene clusters. The left side shows strongly enriched genes, and the blue-to-red boxes of the selected genes indicate the logFC values. The right side shows the enriched GO terms **(E)** Protein–protein interaction network identified using GeneMANIA (direct interaction database) **(F)** Gene-centred KEGG pathway enrichment plot of the screened proteins. The bubble size and color (varying from yellow to red) represent the significance levels of the pathway. *p < 0.05, **p < 0.005, and ***p < 0.0005 compared with control rats; #*p* < 0.05, ##*p* < 0.005, and ###*p* < 0.0005 compared with OVX rats. NS, not significant.

We analyzed the cellular environment using proteomics to further investigate the role of HPL and dissect the proteins contributing to the restorative effects on the estrogen depletion-driven transformation process. A proteomic shift in the cellular environment was observed, as determined by the calculation of relative protein ratios from the tandem mass spectrometry (MS/MS) reporter ions using ProteinPilot software. Among the global proteins in the rat proteome, 35,811 sequences were detected among control, OVX, and high-dose HPL-treated samples using Mascot software (version 2.3.02). We selected and quantified 286 proteins to further improve the reliability (Figure 2A). We then selected differentially expressed proteins based on cut-off conditions of a 1.2-fold change (control *vs.* OVX) and *p* value < 0.05. The abnormal levels of 21 proteins were rescued by the HPL treatment (Figure 2B and Supplementary Tables S1).

All recovered proteins were mapped to various terms in the Gene Ontology (GO) database, and proteins for each term were counted and calculated using a hypergeometric test and mapped to enriched GO terms. GO annotation by IPA indicated that the 21 identified proteins associated with HPL treatment in OVX rats were significantly enriched in “cellular components of the extracellular space” (12 proteins) and “extracellular exosome” (11 proteins) (Supplementary Tables S2). An analysis of the enriched molecular functions revealed that these 21 proteins exhibited a significant functional association with exopeptidase activity (*p* < 0.001, 4 proteins), endopeptidase inhibitor activity (*p* = 0.002, 4 proteins), endopeptidase regulator activity (*p* = 0.0015, 4 proteins), peptidase inhibitor activity (*p* = 0.002, 4 proteins), and peptidase regulator activity (*p* = 0.002, 4 proteins) (Figure 2C and Supplementary Tables S3).

Consistent with the molecular function evidence, the 21 restored proteins were significantly enriched in the biological processes of “negative regulation of endopeptidase activity” (*p* = 0.0002, 4 proteins), “negative regulation of peptidase activity” (*p* = 0.0002, 4 proteins), “regulation of endopeptidase activity” (*p* = 0.001, 4 proteins), and “response to estradiol” (*p* = 0.049, 3 proteins) (Figure 2D and Supplementary Tables S3). Notably, 13 of the 21 proteins had been previously identified as kidney disease markers, including Ambp, alanyl aminopeptidase (Anpep), B2m, C3, Camp, Cdh1, Col1a1, gamma-glutamyltransferase 1 (Ggt1), Myh6, Plau, Ptgds, Slc3a1, and Tff1 (Dussol et al., 2004; Arumugam et al., 2007; Ryu et al., 2007; Humphreys et al., 2008; Holliday et al., 2010; Chen, 2011; Roberts et al., 2014; Jia et al., 2015; Menon et al., 2015; Lu et al., 2016; Flyvbjerg, 2017; Hayek et al., 2017; Ix et al., 2017; Jerebtsova et al., 2017; Rubinow et al., 2017; Wei et al., 2017).

We further examined the interactions of these proteins responsive to HPL treatment using the GeneMANIA platform. All proteins were subjected to a leading edge analysis according to physical interactions, shared protein domains, and colocalization. Seven of those renal disease-related proteins had higher weighted scores (> 0.60), including Ggt1 (0.941), Camp (0.906), Cdh1 (0.835), Lpo (0.833), Scpep1 (0.803), Anpep (0.644), and Ptgds (0.603). Notably, these proteins have a close relationship with “high-density lipoprotein particle”, “response to estrogen”, “response to estradiol”, “exopeptidase activity”, and “peptidase activity” (Figure 2E, see also Supplementary Tables S4–S6). Based on these data, HPL-evoked changes in peptidase activity in the proteomic profile appear to be kidney tissue-dependent changes. Consistent with the GO enrichment analysis, the results indicated that HPL-induced recovery of Ggt1 and Anpep levels might be responsible for the increased blood GSH/GSSH ratio observed in rats with OVX-induced kidney dysfunction.

Consistent with the enriched molecular functions and biological processes, the KEGG pathway enrichment and GeneMANIA protein relationship analyses indicated that HPL mainly rescued the function of the redox metabolism pathway (glutathione metabolism) that was associated with renal redox function.

In view of the GO results, the Kyoto Encyclopedia of Genes and Genomes (KEGG) network enrichment analysis highlighted a greater effect on a metabolic pathway, glutathione metabolism (path:ko00480), which included upregulated Ggt1 and downregulated Anpep, in OVX rats (Figure 2F, and see Supplementary Tables S7).

### HPL Improves Metabolite Profiles From the Ggt1/Anpep-Mediated Glutathione Metabolism Pathway

**FIGURE 3:**
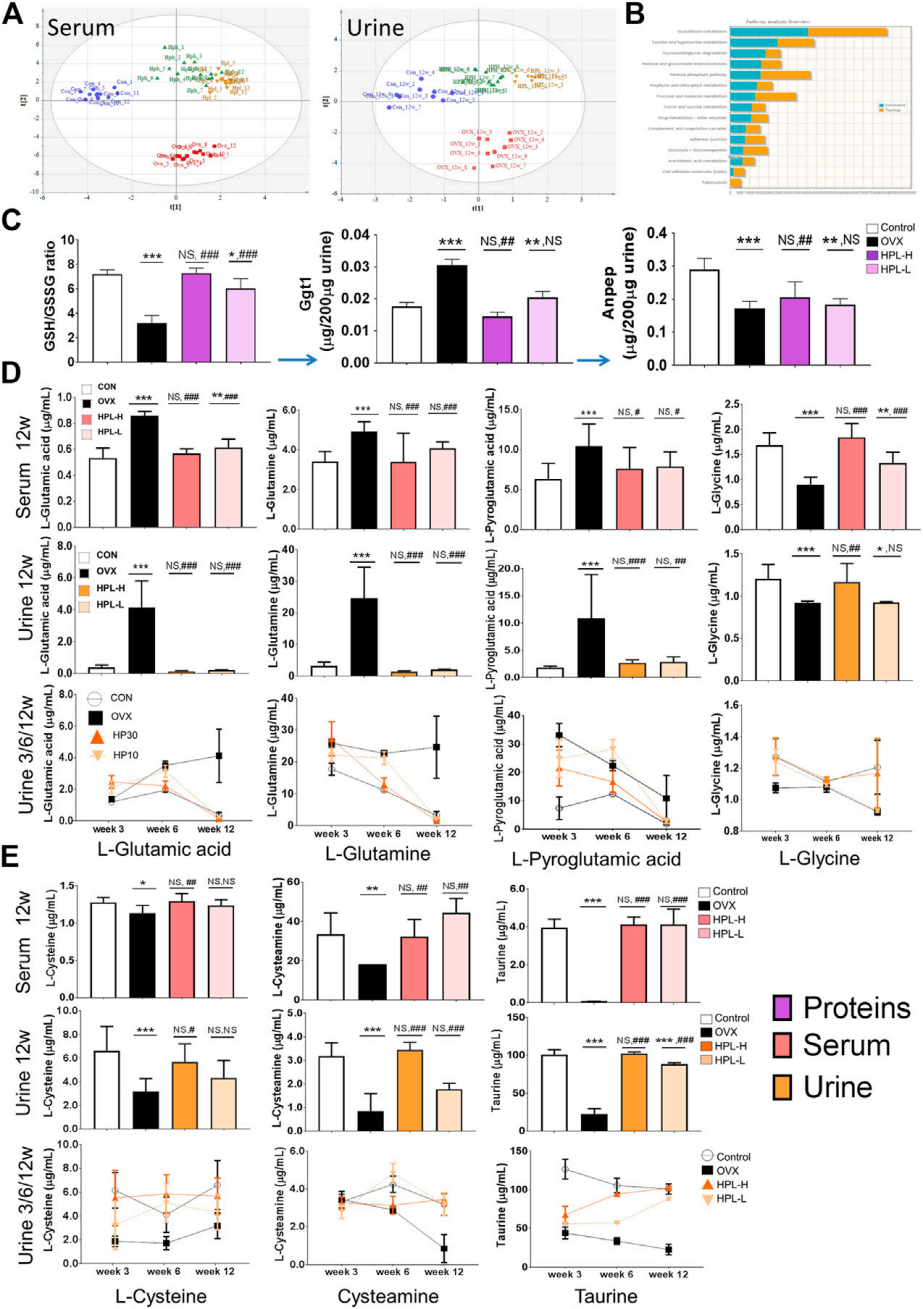
HPL therapy reverses OVX-induced endometabolite imbalances in the glutathione metabolism pathway. **(A)** PLS analysis of the metabolomic profiles of urine and serum samples; the differences among control (blue dot), OVX (red square), HPH (HPL-H, green triangle), and HPL (HPL-L, yellow downward triangle) samples are shown. Each symbol on the 2D score plot represents a result for an individual. **(B)** Gene-metabolite KEGG pathway enrichment plot of the biomarkers in each pathway; the bar length (blue: enrichment and orange: topology) represents the significance levels of the pathways. **(C)** Redox GSH/GSSG ratio and relative peptide quantification for Ggt1 and Anpep. The data are representative of three experiments. The data are pooled from three experiments (*n* = 3, mean ± *s*. e.m.). **(D)** and **(E)** Quantification of metabolites from glutathione metabolism in 3, 6, and 12 weeks urine samples and 12 weeks serum samples using a liquid chromatography–tandem mass spectrometry analysis. The data were pooled from three experiments (*n* = 9, means ± *s*.d.). HPL therapy dosage: HP30, 30 mg/kg/d; HP10, 10 mg/kg/d. *p < 0.05, **p < 0.005, and ***p < 0.0005 compared with control rats; #*p* < 0.05, ##*p* < 0.005, and ###*p* < 0.0005 compared with OVX rats. NS, not significant.

We measured local serum and urine metabolite levels by performing a targeted metabolomic analysis to determine whether endometabolite perturbations in OVX rats were due to the depletion of the substantial redox function and an imbalance in the system. The PLS (partial least squares regression) analysis of the targeted urinary and serum metabolomes showed distinct restorative change in metabolite profiles following HPL treatment (Figure 3A). The highly enriched genes and metabolites were mapped to KEGG metabolic pathways for an overrepresentation analysis and pathway topology analysis using MetaboAnalyst to evaluate the relative importance of the genes/compounds based on their relative locations. “Gene centric pathways” correlation analyses showed that the “glutathione metabolism” pathway (*p* = 0.001) was highly ranked, as well as its linked “taurine and hypotaurine metabolism” pathway (*p* = 0.009) (Figure 3B and see Supplementary Tables S8).

We targeted glutathione metabolism-related proteins and metabolites to further investigate the roles of the enriched metabolite pathways and dissect which of the identified associated proteins contributed to these pathways. Menopausal syndromes have been reported to be associated with altered cellular glutathione levels. Under normal physiological conditions, glutathione is mainly degraded to glutamate and Cys-Gly dipeptide by Ggt1, which belongs to the N-terminal nucleophile hydrolase family. Subsequently, the Cys-Gly dipeptide is degraded by membrane-bound Anpep to yield cysteine and glycine for another glutathione cycle. Accordingly, a systemically decreased shift in the GSH/GSSH ratio in the redox environment was observed in OVX rats, the parameters of which were restored by HPL therapy (Figure 1E).

By measuring the ratio of heavy isotope-labelled peptides in urine samples using liquid chromatography (LC)-MS in multiple reaction monitoring (MRM) mode, we observed significantly higher Ggt1 levels in the OVX rats than in the control rats (Figure 3C, peptide determination results are shown in Supplementary Tables S9). In addition, OVX also induced a marked increase in the Ggt1-catalyzed glutamate levels and cascade glutamine levels in the serum and urine, whereas HPL therapy significantly reversed these changes. Pyroglutamic acid (5-oxoproline), a glutamate cyclized derivative, also presented a higher abundance in OVX rat serum/urine samples that was significantly reversed by HPL therapy (Figure 3D, metabolite determination results are shown in Supplementary Tables S10).

We further investigated whether Ggt1-induced glutamate and its products perturbed downstream pathways. As Anpep also prevents cellular toxicity from Cys-Gly dipeptide accumulation in addition to promoting cysteine and glycine recovery, we next measured urinary Anpep levels using LC-MS in MRM mode (Figure 3C, peptide determination results are shown in Supplementary Tables S9). Notably, Anpep levels were decreased in the OVX rats compared to those in the control rats. This difference corresponded to the decrease in levels of the Anpep-catalyzed products (cysteine and glycine) compared with serum and urine from control rats (Figures 3D,E, metabolite determination results are shown in Supplementary Tables S10). Consistent with the trend of decreased cysteine levels, neuroprotective intermediates in the downstream taurine/hypotaurine metabolism pathway also exhibited a trend toward depletion (Levin et al., 2014) (Figure 3E, metabolite determination results are shown in Supplementary Tables S10). Cysteamine levels were decreased by 30 and 70% in the serum and urine of OVX rats, respectively, compared to sham-treated rats; moreover, the level of the cysteamine metabolite taurine was also decreased by 60% in OVX rats. These trends of alterations in metabolite levels correspond to the proteomic data and indicated that estrogen depletion increased the proteomic phenotype, metabolic induction appeared to be strongly associated with glutathione depletion and oxidative stress in renal cells, and that HPL targeting rescued these trends toward an imbalance.

### HPL Restores the Expression of Key Proteins Involved in Glutathione Metabolism

**FIGURE 4:**
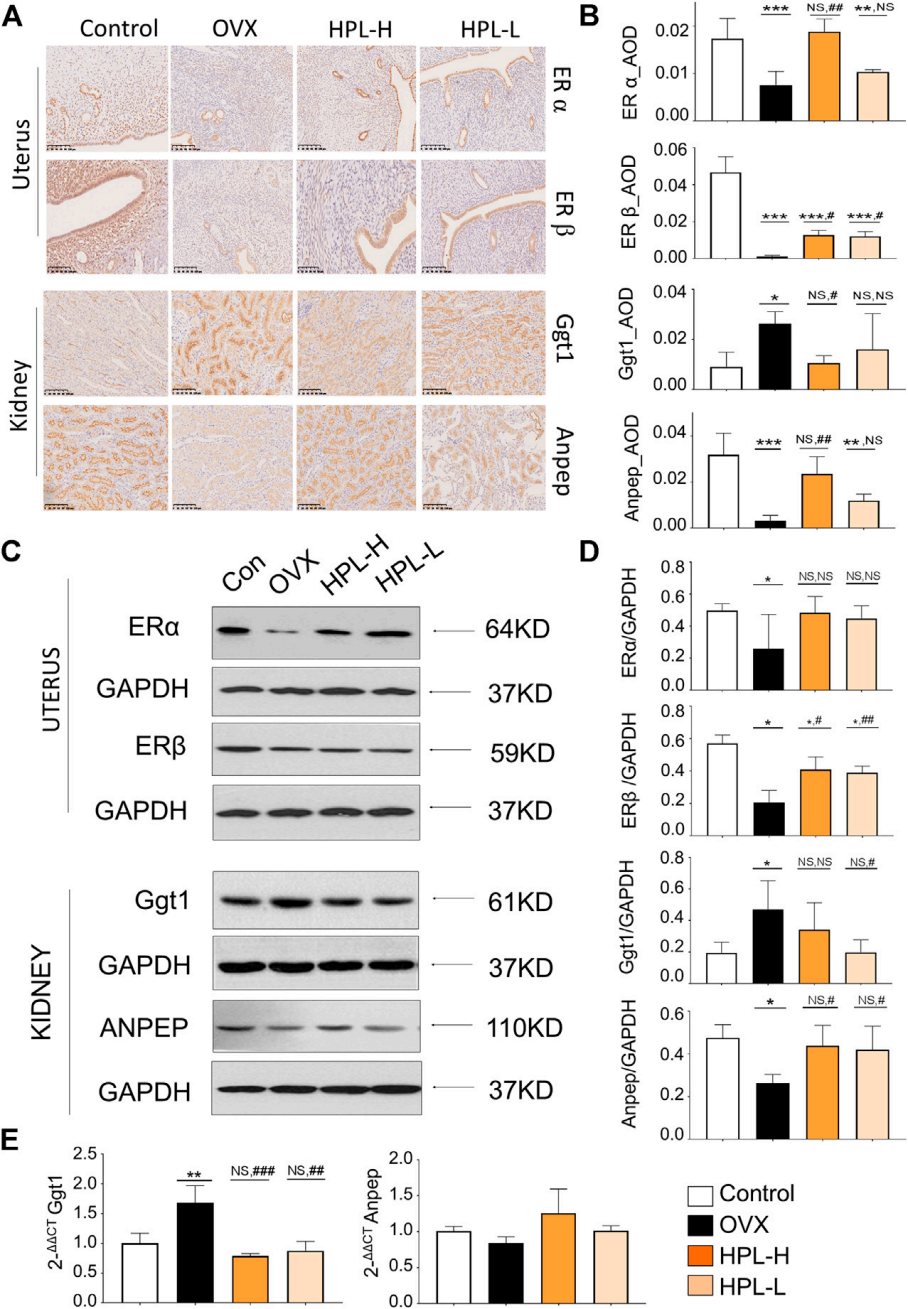
Immunohistochemistry (IHC) and western blot assays of HPL-treated samples. **(A)** IHC revealed a disturbed morphology of uterine and renal tissues (DAB staining, scale bar, 200 µm). **(B)** The IHC average optional density (AOD) ratio. **(C)** Representative images of ERα, ERβ, Ggt1, and Anpep levels detected using western blotting. **(D)** Representative quantitation of ERα, ERβ, Ggt1, and Anpep levels detected using western blotting. **(E)** qRT-PCR analysis of renal levels of the Ggt1 and Anpep mRNAs. The results of the statistical analysis are presented in bar charts (mean ± *s*. e.m.). HPL therapy dosage: HPL-H, 300 mg/kg/d; HPL-L, 80 mg/kg/d. *p < 0.05, **p < 0.005, and ***p < 0.0005 compared with control rats; #p < 0.05, ##p < 0.005, and ###p < 0.0005 compared with OVX rats. NS, not significant.

Estrogen receptor alpha (ERα), estrogen receptor beta (ERβ), Ggt1, and Anpep expression were determined using immunohistochemistry, western blot and qRT-PCR assays to further examine how the HPL treatment influenced renal glutathione redox stress. DAB staining, as indicated by the brown color in the immunohistochemistry analysis, was interpreted as positive expression (Figures 4A,B and see Supplementary Tables S11). Consistent with the proteomic data, positively stained areas were measured by calculating the average optical density (AOD) values of ERα and ERβ in the uteri of the OVX rats and were obviously lighter with a significantly lower AOD value. Ggt1 expression was increased in the renal tubules, whereas Anpep expression was decreased. We next assessed the effects of HPL on the expression of ERα, ERβ, Ggt1, and Anpep using western blot assays (Figures 4C,D and see Supplementary Tables S11 and Supplementary Figure S1). ERβ expression was significantly decreased in the uteri of OVX rats, whereas no obvious decrease in ERα expression was observed. Moreover, a substantial increase in Ggt1 expression was observed in OVX rat kidneys compared to that in the control rats; however, decreased Anpep expression was identified. HPL therapy rescued ERα, ERβ, Ggt1, and Anpep expression at both dosages. Consistent with these data, the qRT-PCR analysis also indicated that HPL administration (dose-dependent) significantly restored renal Ggt1 expression to the control levels, and a positive effect of HPL on Anpep expression was also observed. (Figure 4E and see Supplementary Tables S11). Thus, HPL therapy reversed OVX-induced redox stress and alleviated glutathione metabolism, which is associated with kidney dysfunction.

## Discussion

This study reveals a link between the effects of HPL on OVX-induced kidney dysfunction and glutathione redox stress. In view of our previous findings, the effect of HPL on the redox system is likely a function of its flavonoids. Consistent with this result, HPL flavonoids with a typical flavonol “C6–C3–C6” carbon framework exhibited stable free radical scavenging activity by interacting with free radicals to form stable adducts and then generating neutral molecules via retro-Diels-Alder degradation (Liu et al., 2014a). Similar data indicate that HPL flavonoids initiate cytoprotective effects on oxidative stress by increasing the activities of antioxidant enzymes such as GSH-Px, SOD, and catalase in the kidney (Xing et al., 2015). However, the cellular and molecular mechanisms underlying the effect on menopause redox imbalance-induced kidney dysfunction remain unclear.

Under conditions of estrogen deficiency, the macromolecular enzymes that maintain the balance of free radicals in the body exhibit reduced free radical scavenging activities. Subsequent oxidative stress results in the age-dependent peroxidation of proteins, lipids, and nucleic acids by intracellular mitochondria, destruction of the mitochondrial membrane permeability, and reduction of oxygen respiratory chain activity, leading to an increase in the number of active oxygen species (Gotoh et al., 2012; Oveland et al., 2012; Gonzalez-Garcia et al., 2018). Accordingly, we previously found that estrogen deficiency-induced menopausal syndromes are caused by the joint regulation of various biological factors. A notable feature of this process is that physiological stress adaptability is reduced, which might increase the susceptibility of the body to various diseases, including hot flashes, adiposity, cognitive degeneration, and depression. Conversely, the administration of HPL (or herbs containing this agent) rescued these phenotypes.

The nephroprotective action of HPL during menopause also deserves special consideration. The association between menopause and the kidney status is consistent with data from an epidemiological study revealing that postmenopausal women are susceptible to chronic kidney disease, which is characterized by diabetic nephropathy, hypertension, and central obesity. A common comorbidity in menopausal women is closely linked to the process of chronic kidney disease development, which increases mortality (Chevalier, 2018; Yun et al., 2018). As estrogen deficiency-induced redox imbalance may contribute to renal deterioration. Based on the data presented in this paper, HPL treatment of OVX rats improves renal function, as concluded from the normalization of renal serum profiles (urea, glucose, and creatinine concentration) and an increase in reactive oxygen species scavenger activities (SOD and GSH).

**FIGURE 5:**
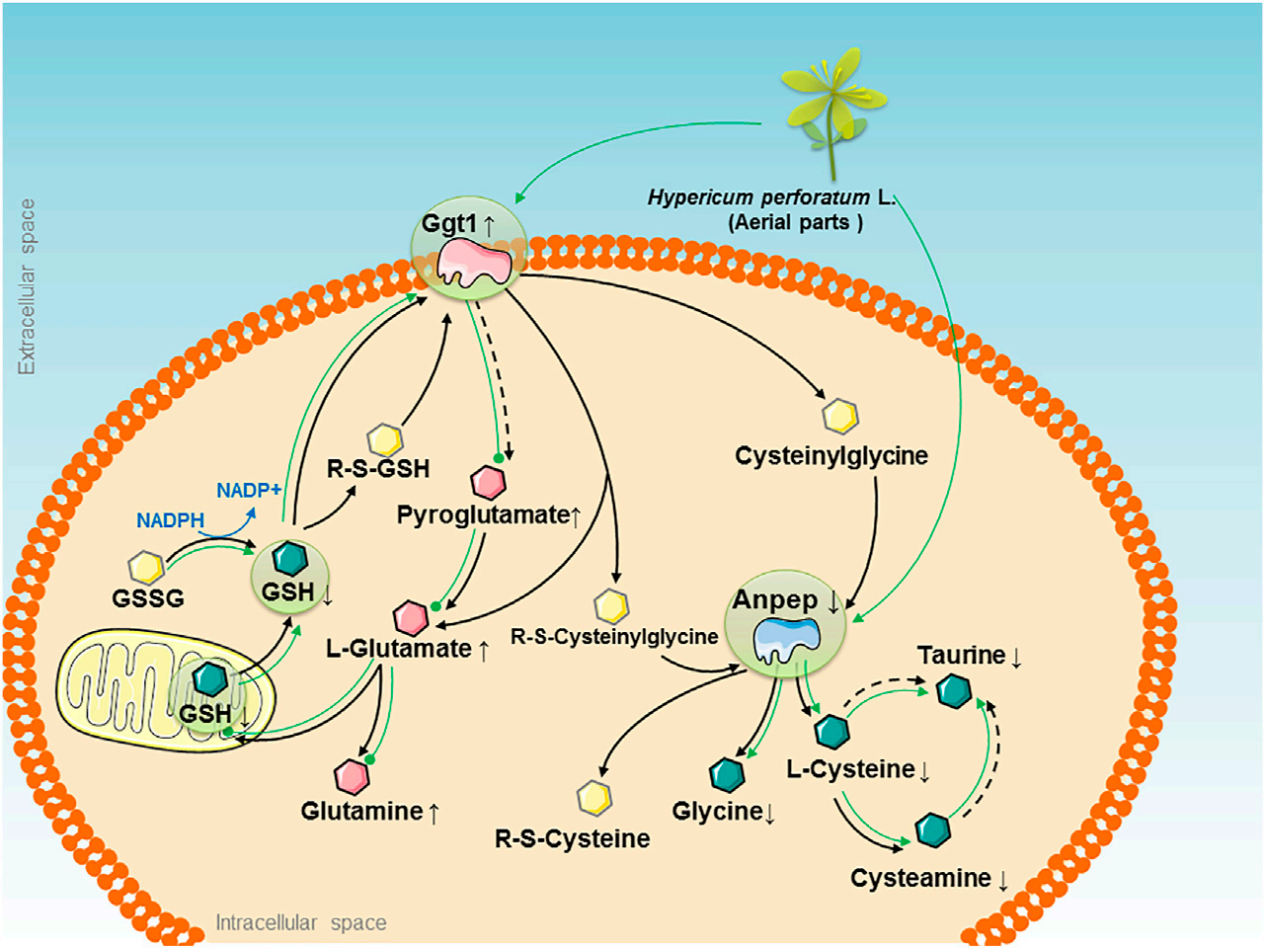
The therapeutic “big picture” of “HPL-disease-gene-metabolite” networks modulated by the HPL treatment in rats with OVX-induced glutathione redox stress. OVX-induced glutathione redox stress triggered abnormalities in pathways that are presented as black solid arrows that link the main glutathione metabolism pathway, and black dotted arrows indicate indirect catalytic reactions. Significant upregulation of Ggt1 and Anpep is represented by pink and blue irregular shapes, respectively. The increased and decreased levels of quantified metabolites are represented by pink and green hexagons, respectively. Other metabolites are represented by yellow hexagons. The effects of HPL therapy on reversing the increased levels of metabolites are represented by dotted green lines, while the effects on restoring the decreased levels of metabolites are represented by solid green arrows.

We analyzed multiple sets of “-omics” data individually and then combined the resulting “HPL-disease-gene-metabolites” to generate a therapeutic “big picture” and to explore the possible nephroprotective mechanisms of HPL in OVX rats (Figure 5). We identified three main findings. First, by integrating the power of networks and evidence-based biological knowledge, the selected genes and metabolites that were associated with HPL therapy were co-projected onto the networks, indicating an important link between OVX-induced kidney abnormalities and intercellular signaling along with the glutathione metabolism pathway.

Second, HPL exhibited the potential ability to maintain cellular redox homeostasis by inhibiting Ggt1 overexpression and decreasing glutamate accumulation. Ggt1, which is expressed at high levels in renal proximal tubules, is considered the central enzyme that maintains the systemic redox balance by hydrolyzing glutathione to glutamate and Cys-Gly via the glutathione pathway. Published *in vivo* studies have indicated that elevated Ggt1 activity along with cellular glutathione depletion are associated with the increased oxidative stress induced by estrogen deficiency under ovariectomy or menopause conditions (Serviddio et al., 2002; Inoue, 2016). However, regardless of the enzyme that initiates glutathione degradation, glutamate is one of the invariant products formed. Glutamate serves as an important excitatory neurotransmitter in the central nervous system. When bound to a glutamate receptor such as the N-methyl-d-aspartate receptor, it initiates synaptic transmission. As these processes are protected and modulated by estrogen, loss of estrogen following OVX reduces the level of glutamate receptors and excitatory glutamate accumulation to influence functional plasticity in prefrontal cortical neurons, which increases the risk of developing neurological insults such as stroke, dementia, Huntington’s disease, and Alzheimer’s disease (Fonnum, 1984; Wei et al., 2014; Waters et al., 2019). In addition, glutamate is catalyzed by glutamine synthetase in the liver to synthesize glutamine; glutamic acid decarboxylase produces gamma-aminobutyric acid (GABA). A decrease in the GABA concentration is related to memory loss (Downs and Wise, 2009; McCarthy, 2011). Because Ggt1 overexpression and abnormal glutamate accumulation damage cells, these excess glutathione conjugates must be cleared from the cell, followed by subsequent glutathione metabolite recycling to protect the system from toxin-induced kidney impairment and maintain the redox balance. Based on the OVX model, we hypothesized that the role of HPL in estrogen depletion-induced effects on glutathione redox stress might be mediated by modulating the levels of key proteins during glutathione metabolism. In subsequent studies, oral administration of HPL reversed the significant increase in Ggt1 expression elicited by OVX and promoted the efflux of the toxic levels of accumulated glutamate and its metabolites.

Third, based on the data presented in this paper, HPL exhibited the potential to restore Anpep expression in the glutathione metabolism pathway and turnover-induced metabolite shift, which reflect the powerful effect of HPL on glutathione redox stress. Anpep, also known as aminopeptidase N or CD13, constitutes a multifunctional membrane-bound zinc-dependent metalloprotease that is ubiquitously conserved in renal, intestinal, placental, brain, and hepatic tissues and is also expressed on monocytes and myeloid progenitor cells (Dringen et al., 2001; Rawlings et al., 2006). Upon degradation by Anpep, cysteine participates in oxidation-reduction reactions, providing sulfides in the body to regulate proliferation, ERβ mRNA expression, and estrogen response element activity (Han et al., 2016). Taurine, which is further catalyzed from Cys by cysteine sulfinic acid decarboxylase, exerts an effect as an inhibitory neurotransmitter on the central nervous system (Renteria et al., 2004; Kim and Kim, 2005). Therefore, consistent with our previous research, we found that HPL therapy restored OVX-induced fluctuations in the levels of amino acids from the Cys and taurine metabolism pathways, such as Cys, cysteamine, glycine, dimethylglycine, or taurine, which might be attributed to reduced Anpep expression as a consequence of glutamate accumulation.

## Conclusion

In view of our findings, data presented in this paper indicate that HPL promotes an amelioration of OVX-induced glutathione redox stress and kidney dysfunction. This action leads to increased GSH activity through the regulation of Ggt1 and Anpep signaling, an action that is involved with estrogen depletion induced glutathione metabolism balance attenuation, specifically in kidney function. In this context, our experiment provide a comprehensive proteomic and metabolomic description for HPL treatment effects on OVX-induced renal redox stress at multiple levels and suggest that HPL administration might be beneficial for the therapy of menopausal kidney dysfunction.

## Data Availability

The data generated in this study can be found in ProteomeXchange: http://proteomecentral.proteomexchange.org/cgi/GetDataset?ID=PXD023104.

## Glossary

ALB: albumin, Anpep: alanyl-aminopeptidase
AOD: average optical density (IOD area)
CREA: creatinine
CKD: chronic kidney disease
CUR: curtain gas
Cys-Gly: l-cysteine-l-glycine
DAB: 3,3-diaminobenzidine
ERα: estrogen receptor alpha
ERβ: estrogen receptor beta
ESI: electron spray ionization
GABA: gamma-aminobutyric acid
GAPDH: glyceraldehyde 3-phosphate dehydrogenase
Ggt1: gamma-glutamyltransferase 1
GLU: glucose
GO: Gene Ontology
GSH-Px: glutathione peroxidase
GSH/GSSG ratio: reduced/oxidized glutathione ratio
H&E: hematoxylin and eosin
HPL: Hypericum perforatum L.
HRT: hormone replacement therapy
IHC: immunohistochemistry
iod: integrated optical density
IPA: Ingenuity Pathway Analysis
iTRAQ: isobaric tag for relative and absolute quantitation
KEGG: Kyoto Encyclopedia of Genes and Genomes
LC: liquid chromatography
MDA: malondialdehyde
MRM: multiple reaction monitoring
MS: mass spectrometry
OVX: ovariectomized
PBS: phosphate-buffered saline
PLS: partial least squares regression analysis
qRT-PCR: quantitative reverse transcriptase polymerase chain reaction
SOD: superoxide dismutase
TBS-T: triethanolamine-buffered saline solution containing Tween
TOF: time of flight
UA: uric acid
WB: western blot
