# Supplementary figures and images for: Hypericum perforatum L. Regulates Glutathione Redox Stress and Normalizes Ggt1/Anpep Signaling to Alleviate OVX-Induced Kidney Dysfunction
Source: Front Pharmacol. 2021 Apr 26;12:628651. doi: 10.3389/fphar.2021.628651 (PMC8109178; doi:10.3389/fphar.2021.628651)

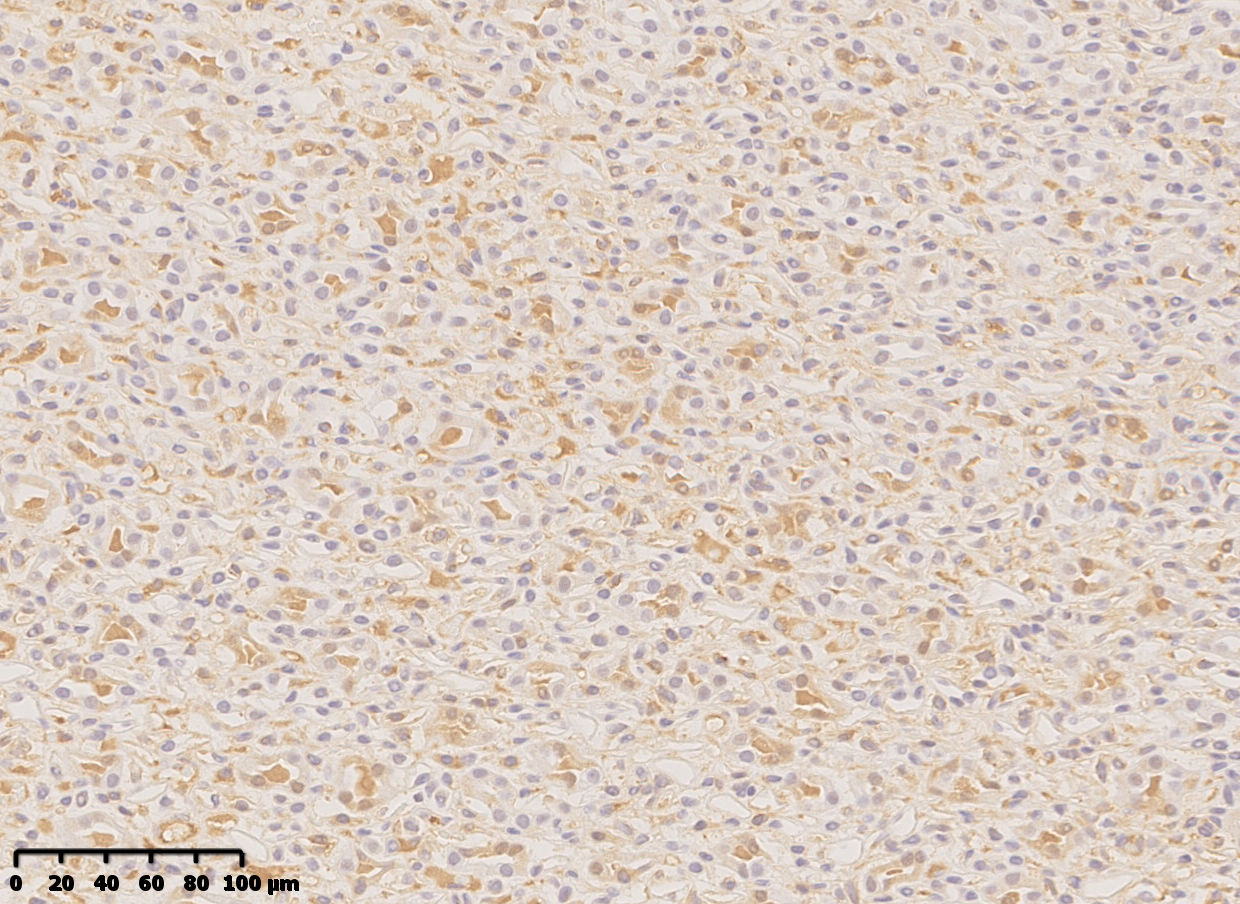

Supplement: Supplementary file 1 [file datasheet1.zip › ╣Γ├▄╢╚╔¿├Φ_final/CD13/CD_HPH-1.jpg]

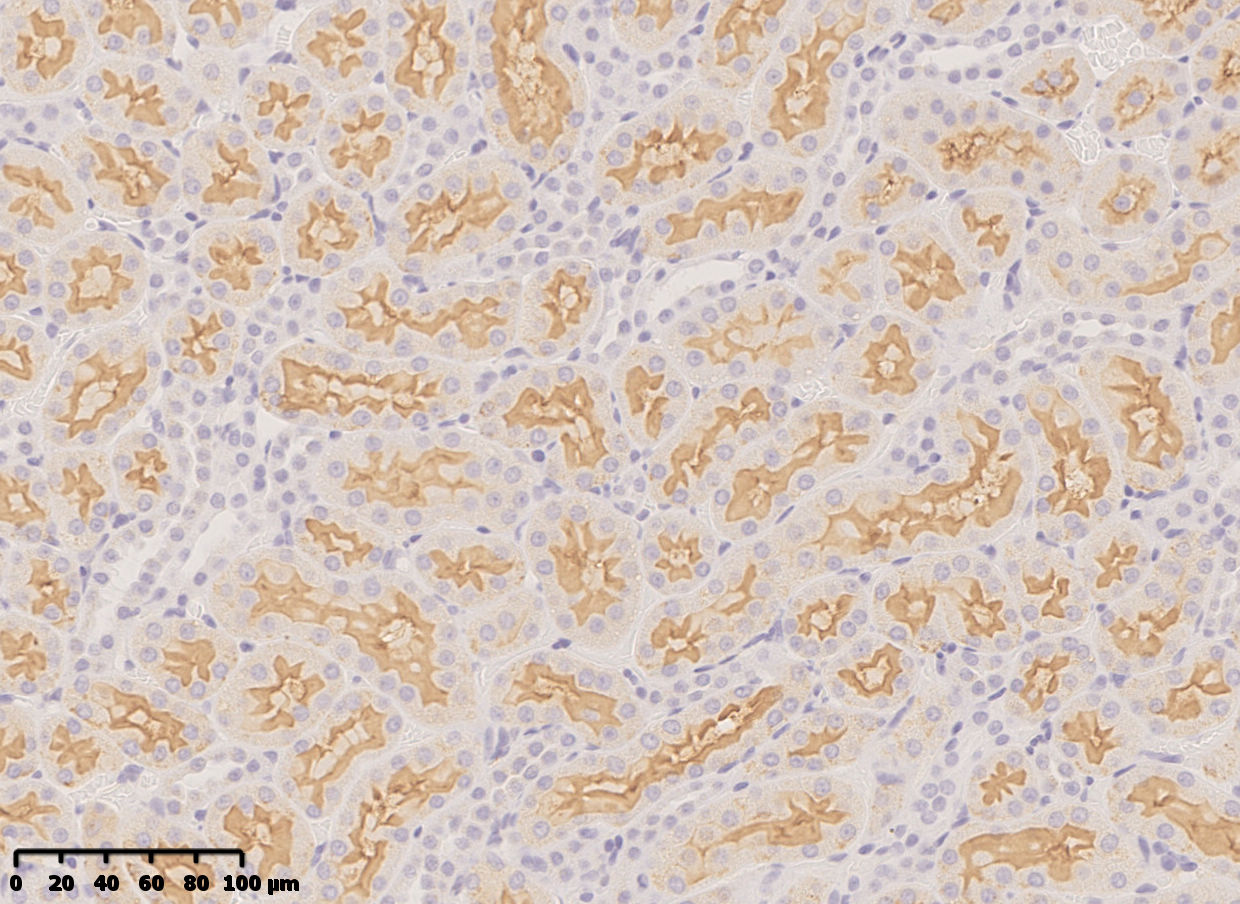

Supplement: Supplementary file 1 [file datasheet1.zip › ╣Γ├▄╢╚╔¿├Φ_final/CD13/CD_HPH-2.jpg]

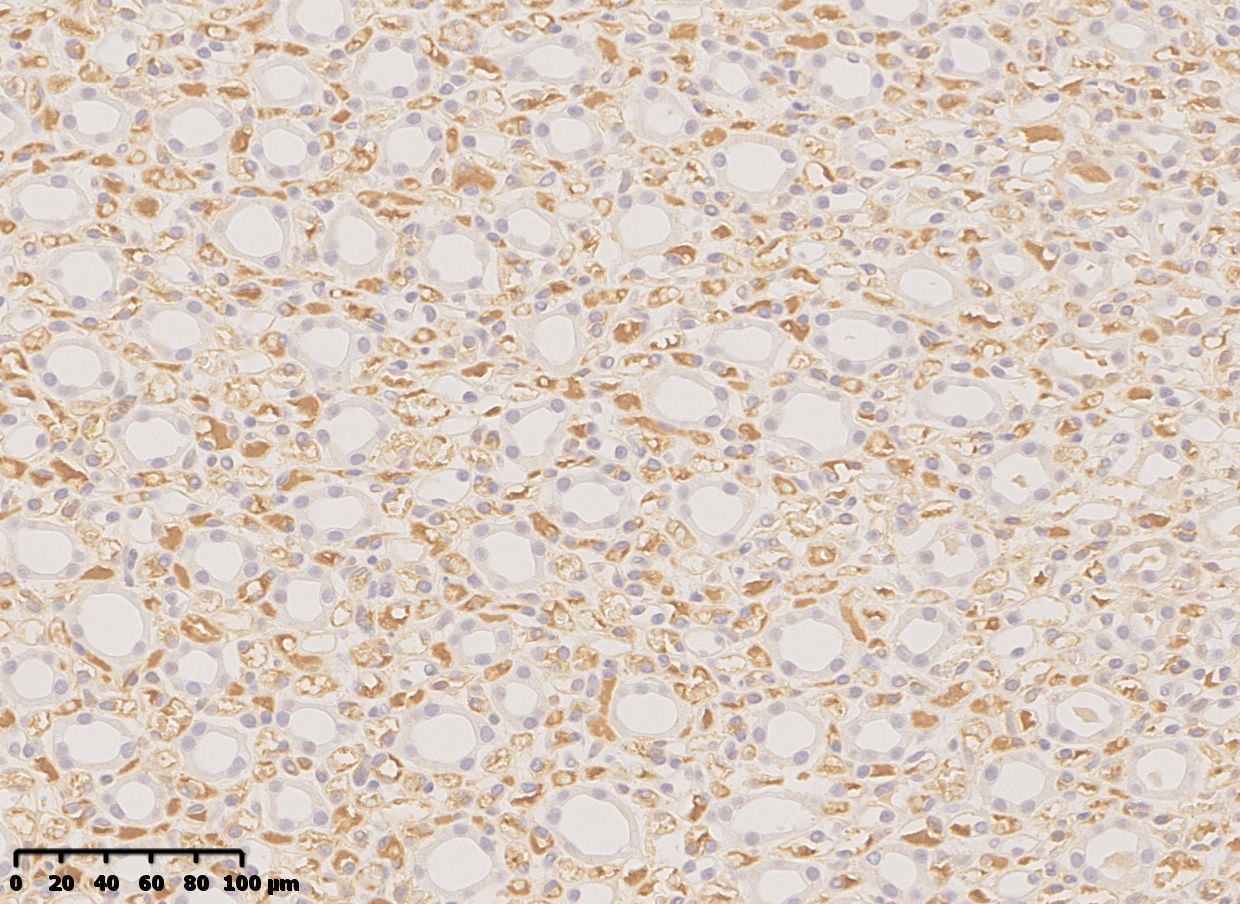

Supplement: Supplementary file 1 [file datasheet1.zip › ╣Γ├▄╢╚╔¿├Φ_final/CD13/CD_HPH-3.jpg]

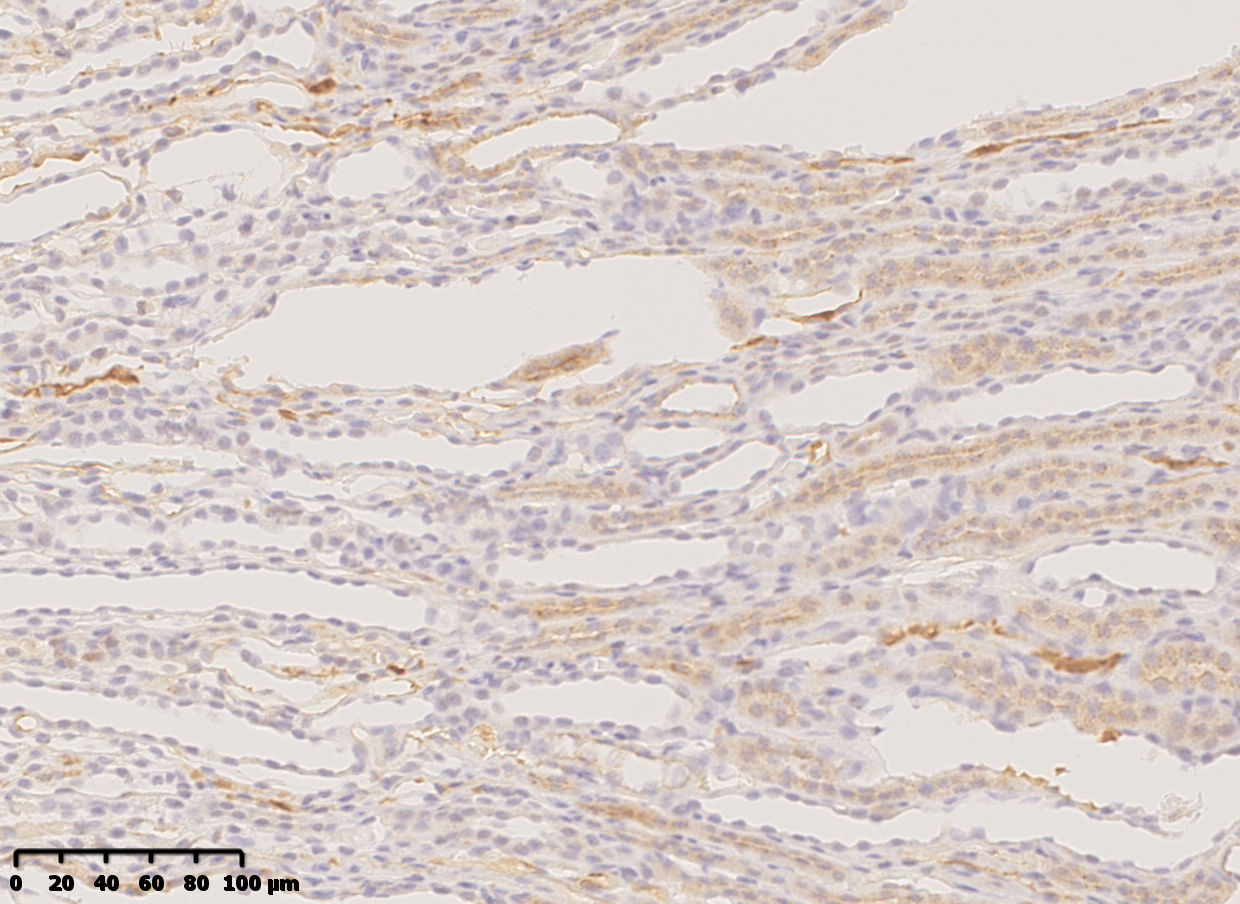

Supplement: Supplementary file 1 [file datasheet1.zip › ╣Γ├▄╢╚╔¿├Φ_final/CD13/CD_HPL-1.jpg]

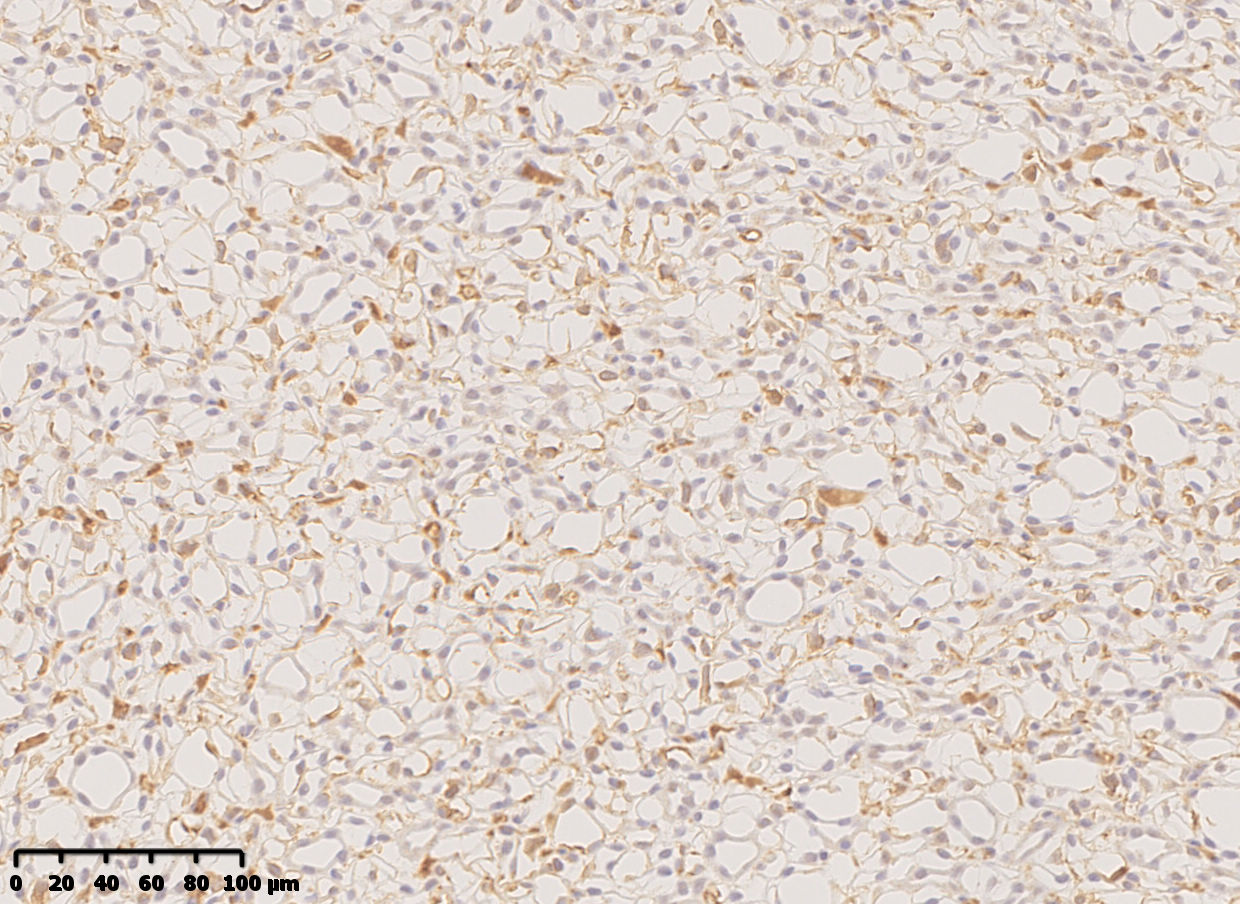

Supplement: Supplementary file 1 [file datasheet1.zip › ╣Γ├▄╢╚╔¿├Φ_final/CD13/CD_HPL-2.jpg]

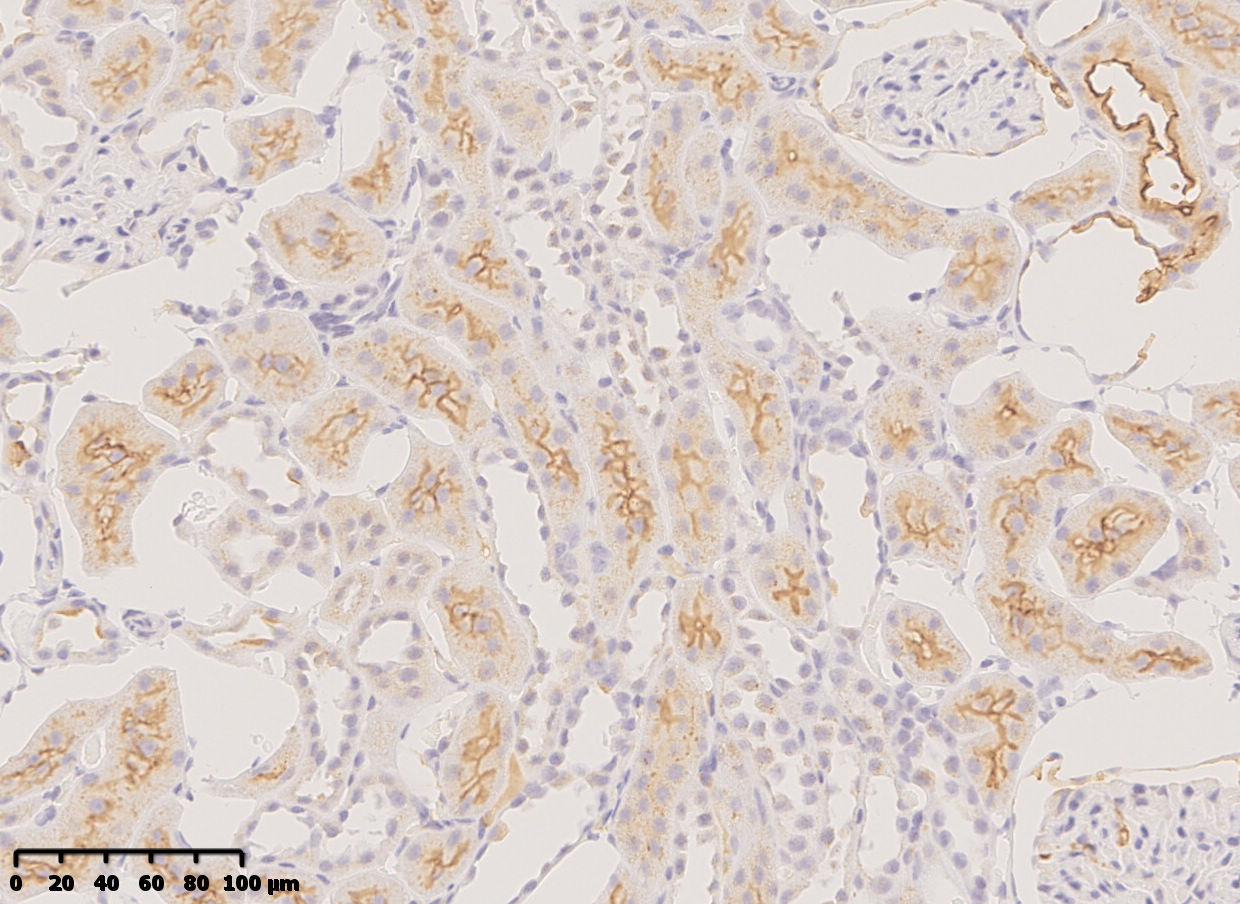

Supplement: Supplementary file 1 [file datasheet1.zip › ╣Γ├▄╢╚╔¿├Φ_final/CD13/CD_HPL-3.jpg]

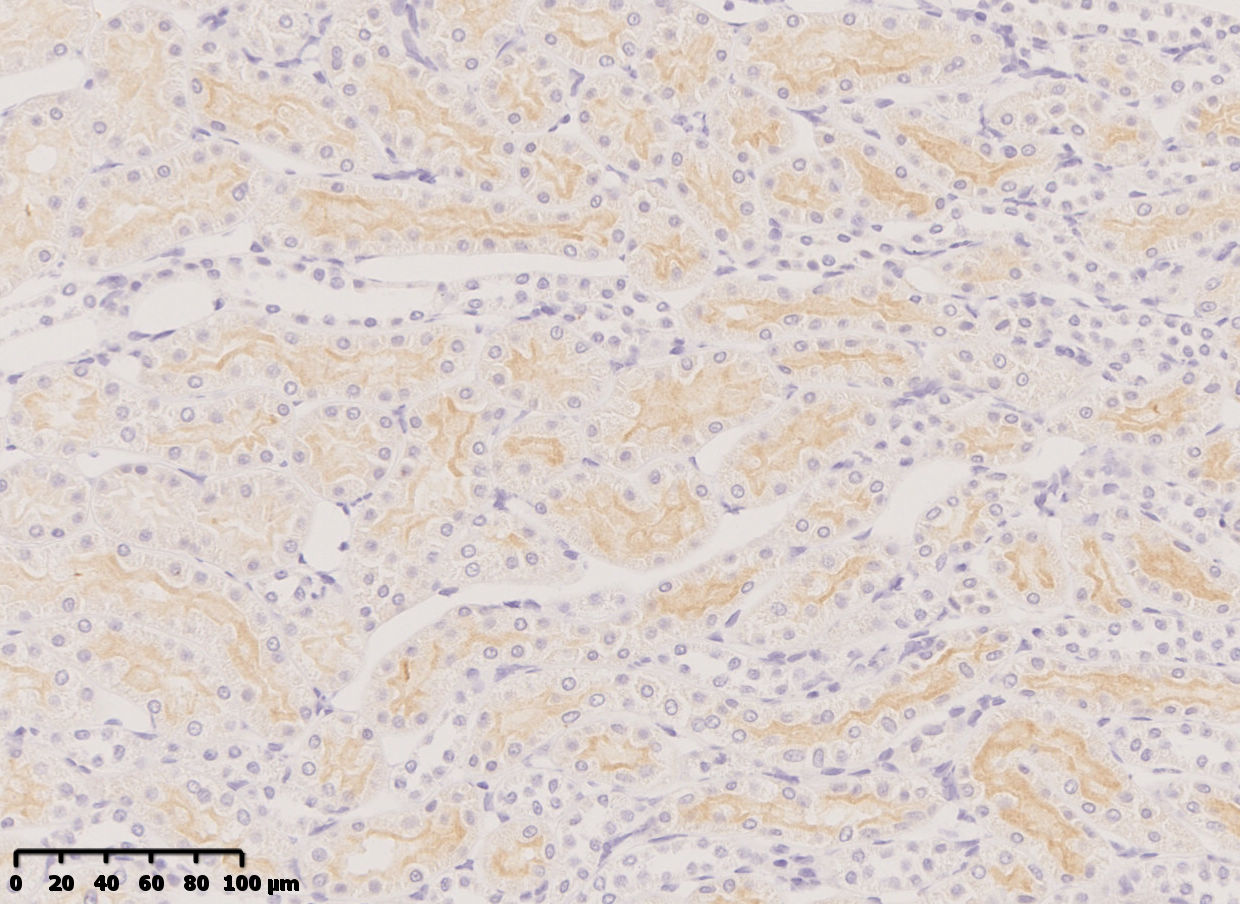

Supplement: Supplementary file 1 [file datasheet1.zip › ╣Γ├▄╢╚╔¿├Φ_final/CD13/CD_OVX-1.jpg]

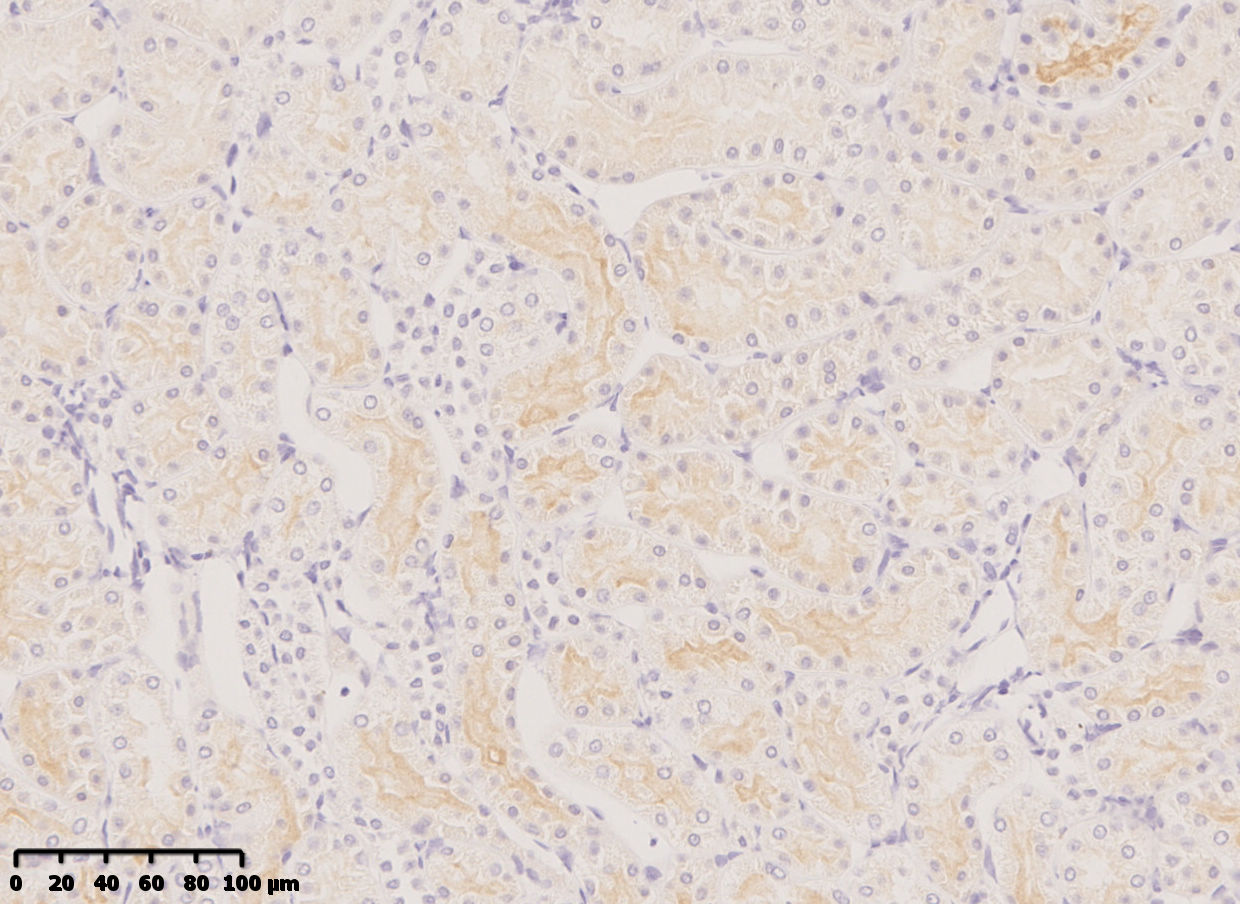

Supplement: Supplementary file 1 [file datasheet1.zip › ╣Γ├▄╢╚╔¿├Φ_final/CD13/CD_OVX-2.jpg]

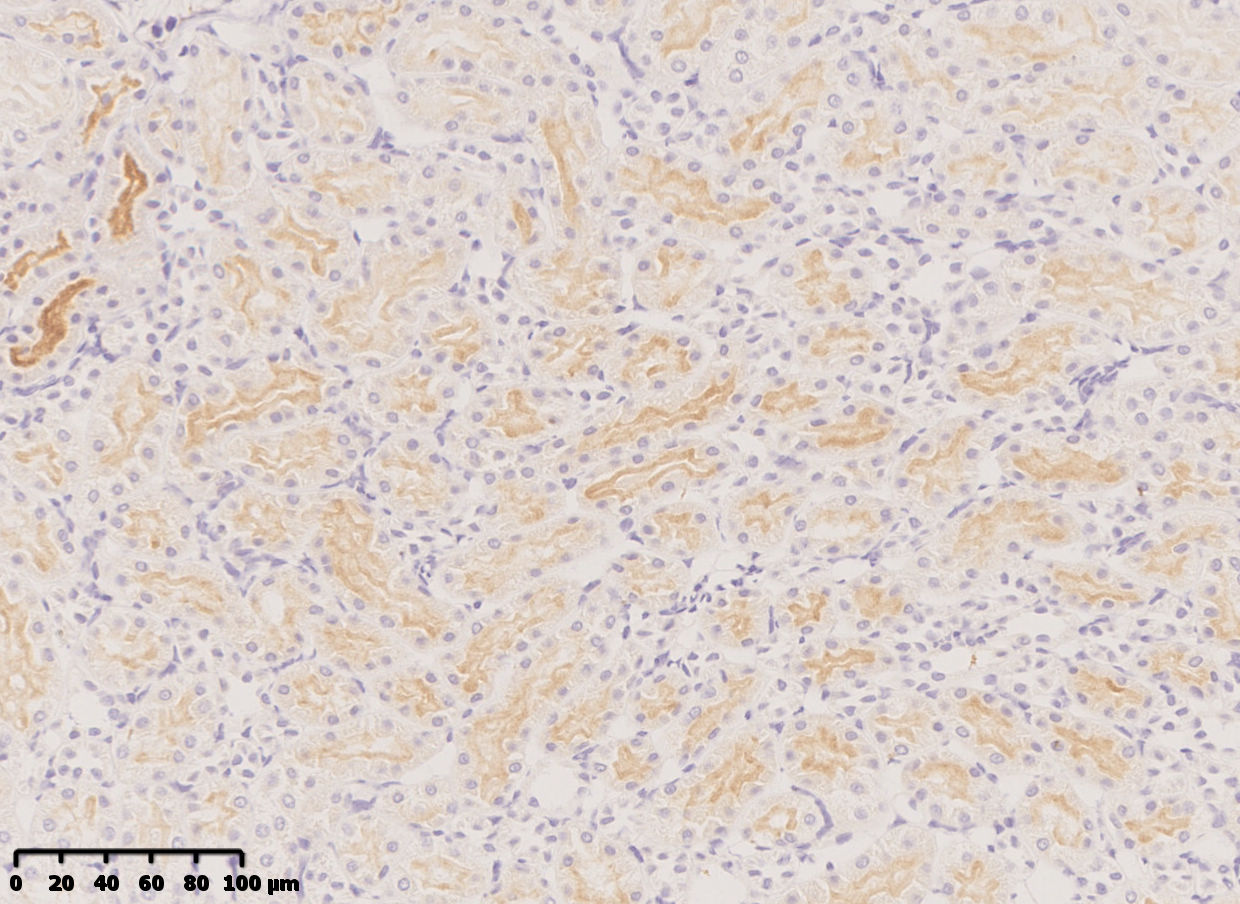

Supplement: Supplementary file 1 [file datasheet1.zip › ╣Γ├▄╢╚╔¿├Φ_final/CD13/CD_OVX-3.jpg]

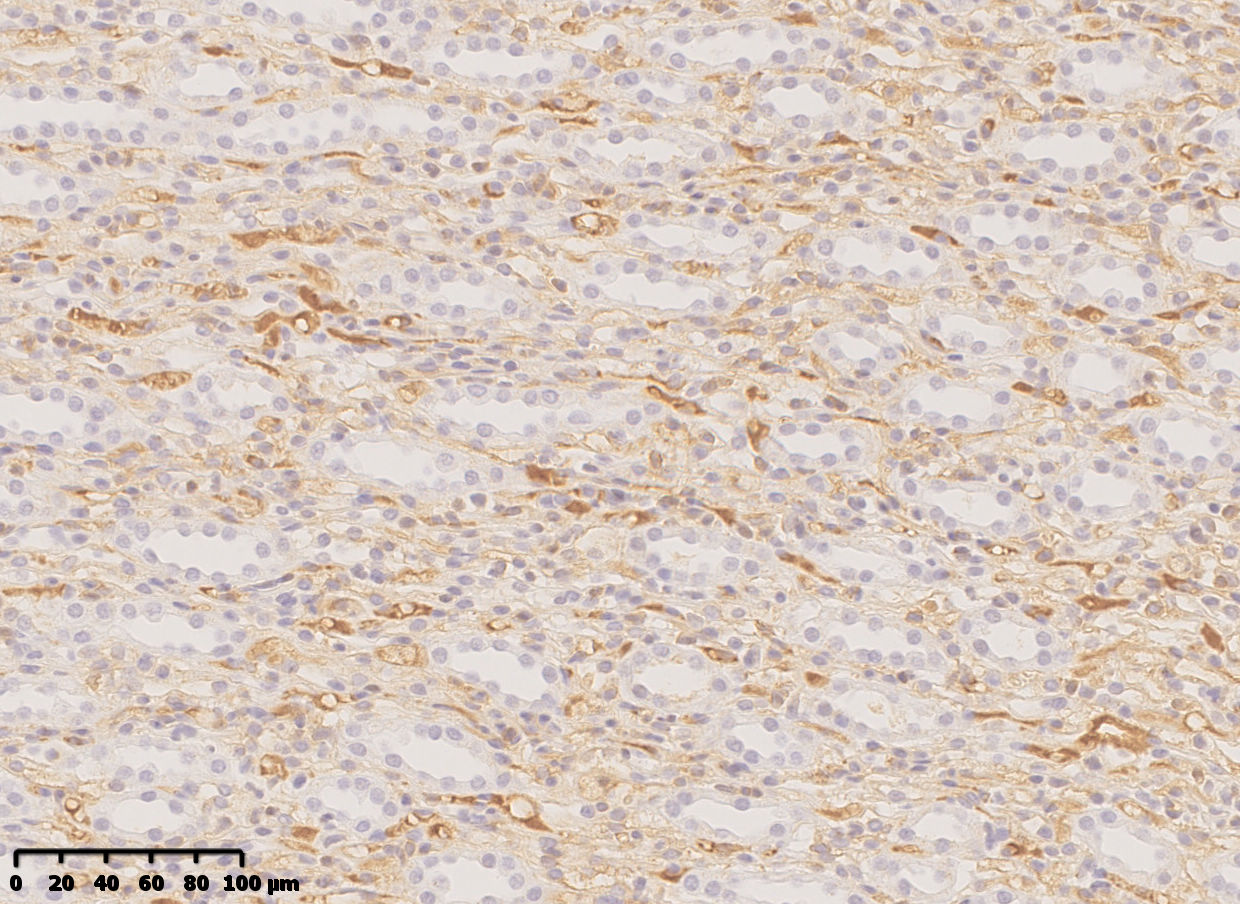

Supplement: Supplementary file 1 [file datasheet1.zip › ╣Γ├▄╢╚╔¿├Φ_final/CD13/CD_con-1.jpg]

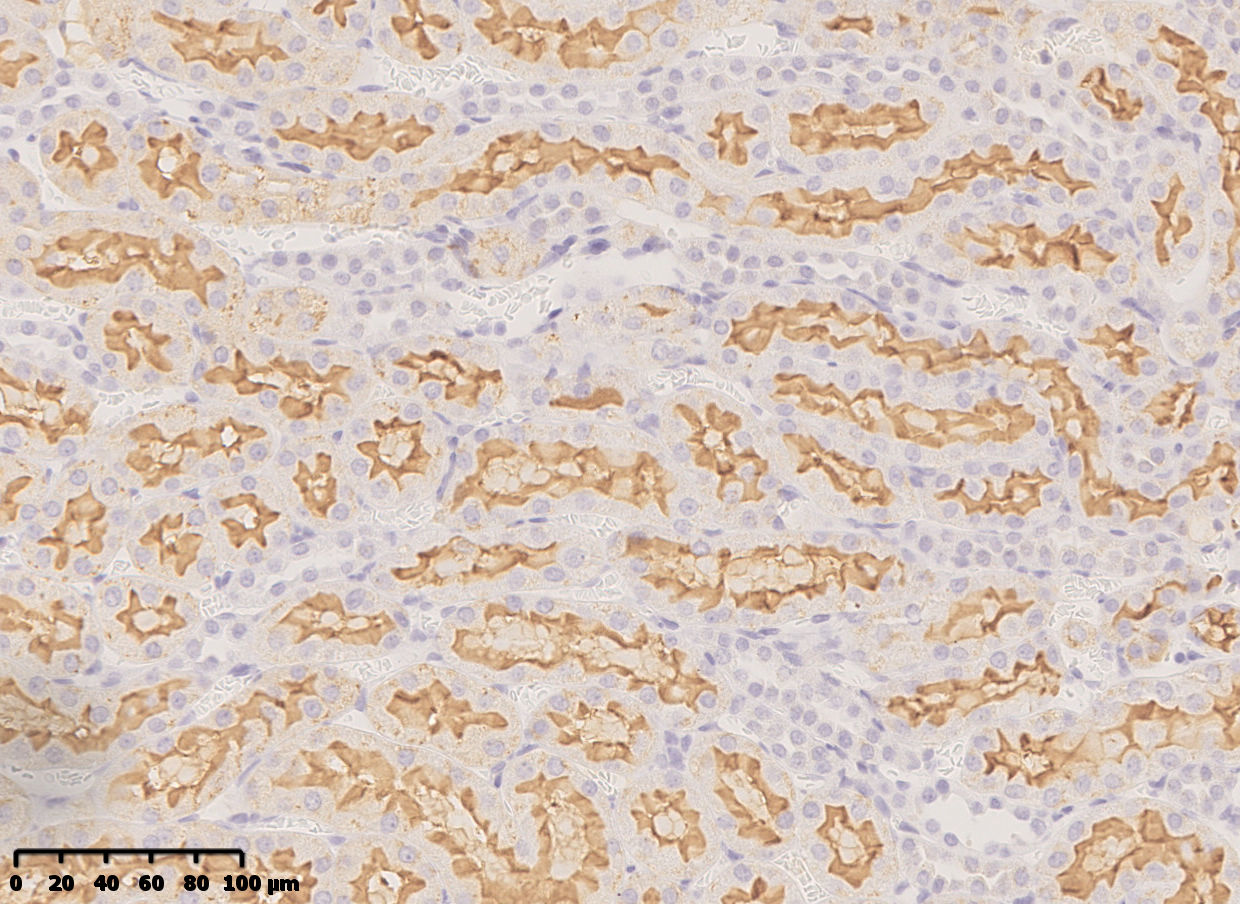

Supplement: Supplementary file 1 [file datasheet1.zip › ╣Γ├▄╢╚╔¿├Φ_final/CD13/CD_con-2.jpg]

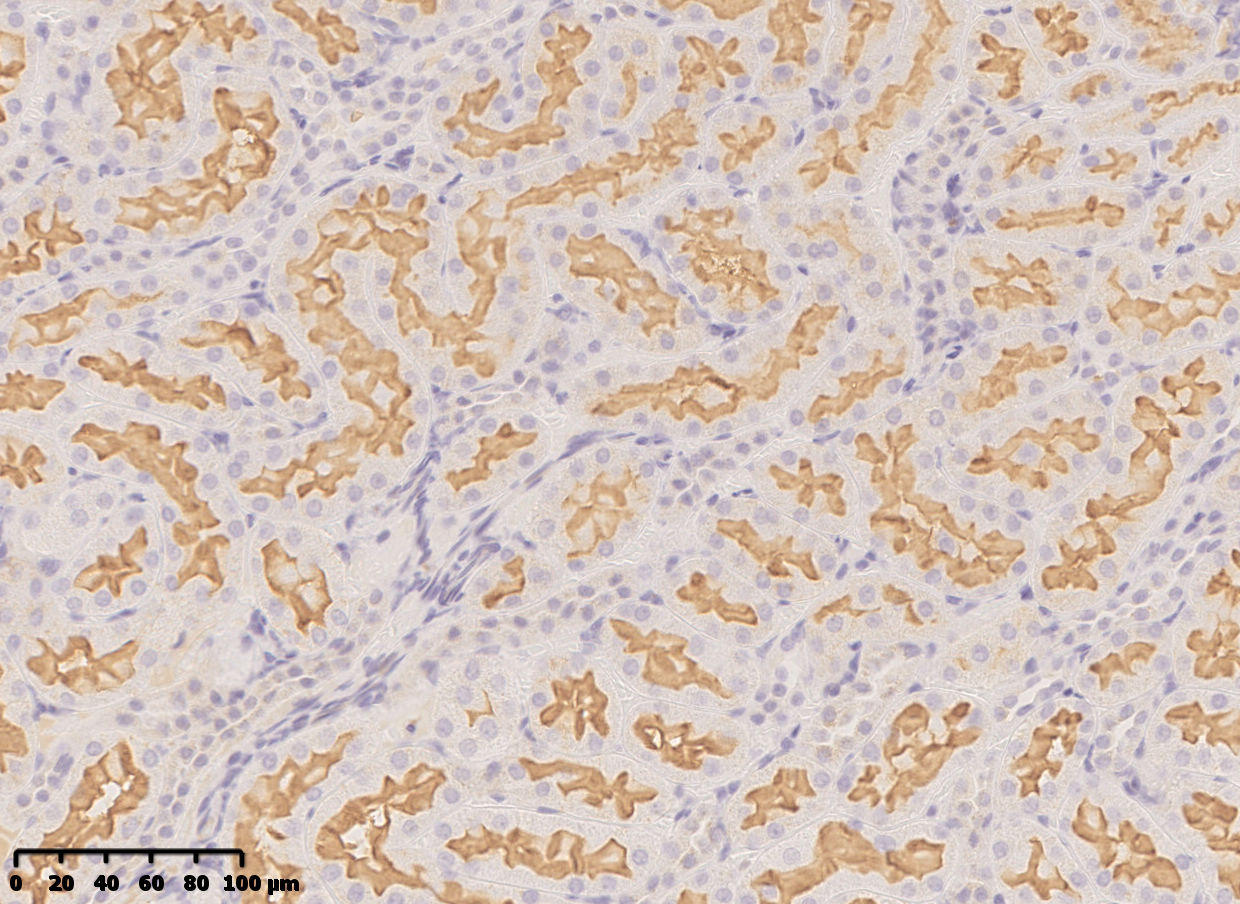

Supplement: Supplementary file 1 [file datasheet1.zip › ╣Γ├▄╢╚╔¿├Φ_final/CD13/CD_con-3.jpg]

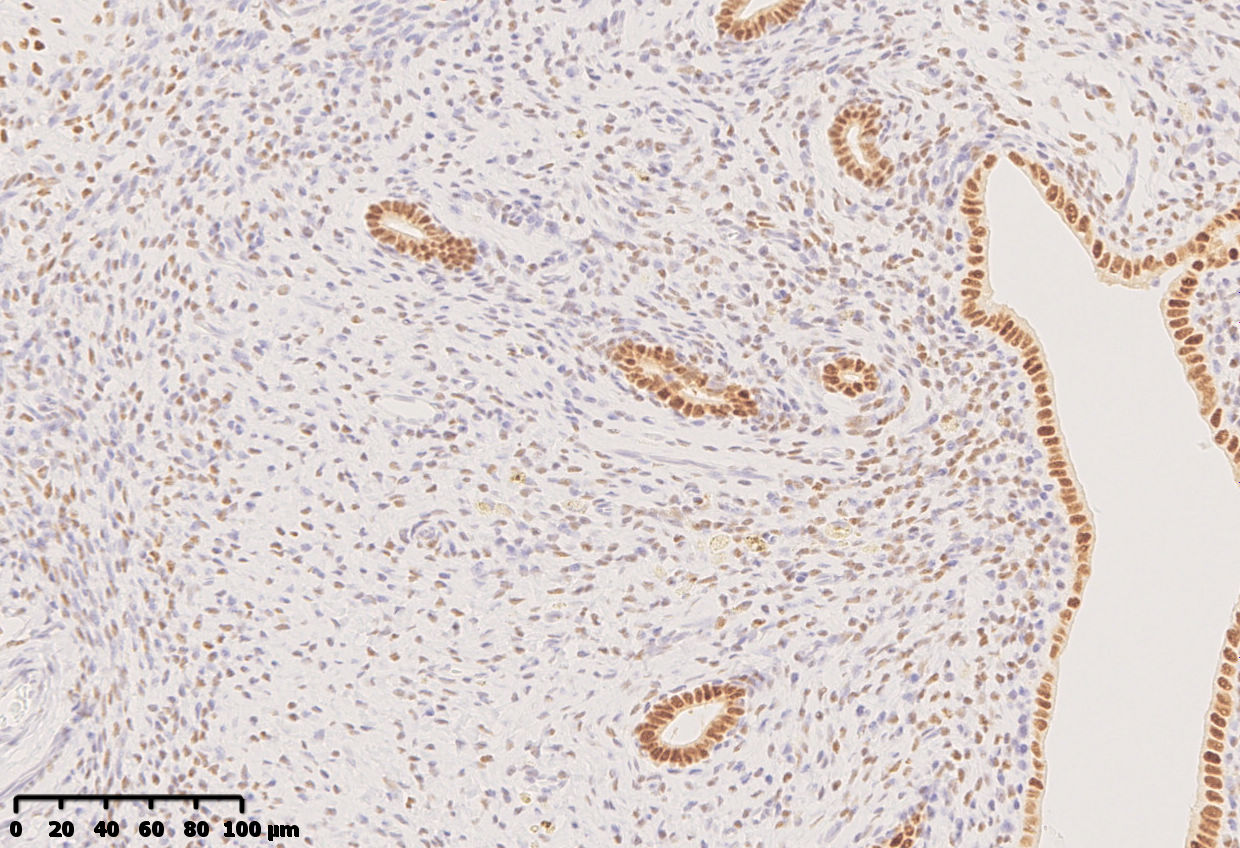

Supplement: Supplementary file 1 [file datasheet1.zip › ╣Γ├▄╢╚╔¿├Φ_final/ERA/ERA_HPH-1.jpg]

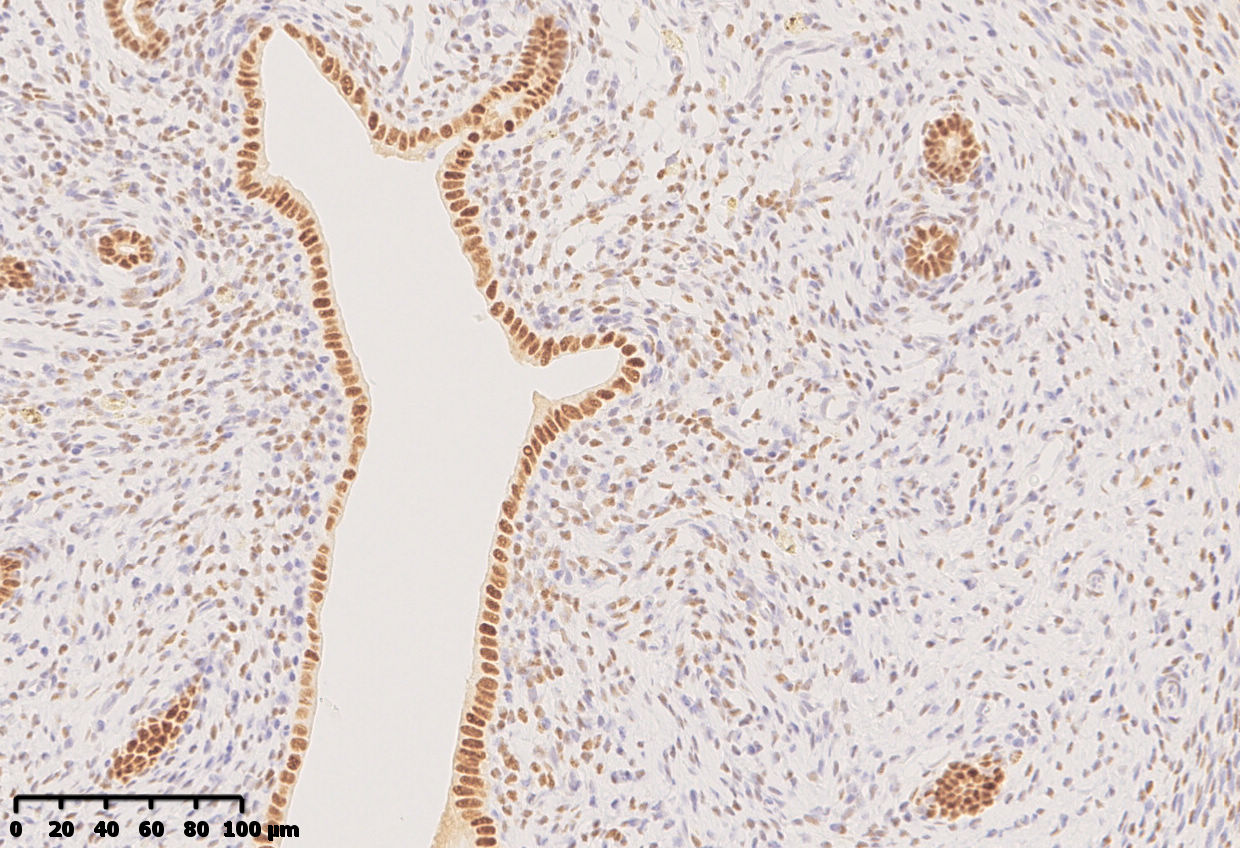

Supplement: Supplementary file 1 [file datasheet1.zip › ╣Γ├▄╢╚╔¿├Φ_final/ERA/ERA_HPH-2.jpg]

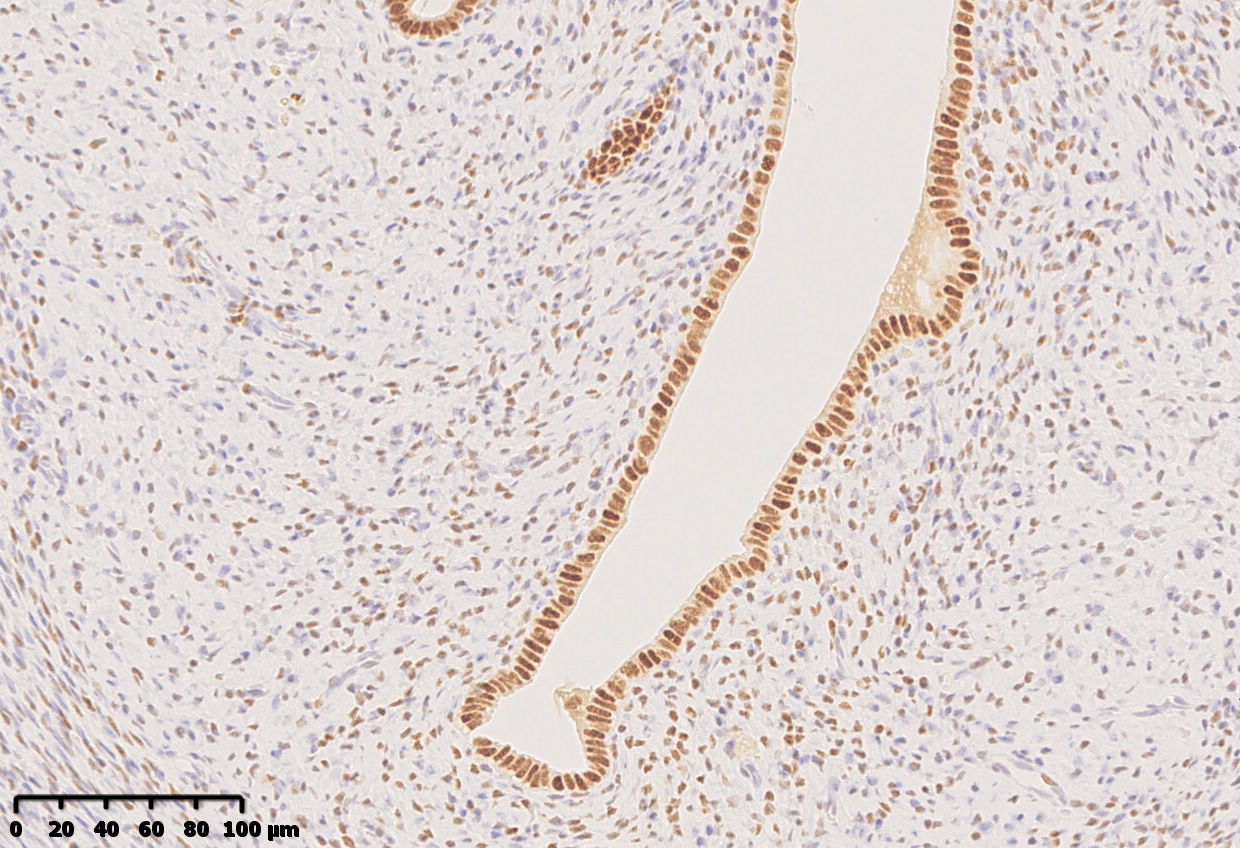

Supplement: Supplementary file 1 [file datasheet1.zip › ╣Γ├▄╢╚╔¿├Φ_final/ERA/ERA_HPH-3.jpg]

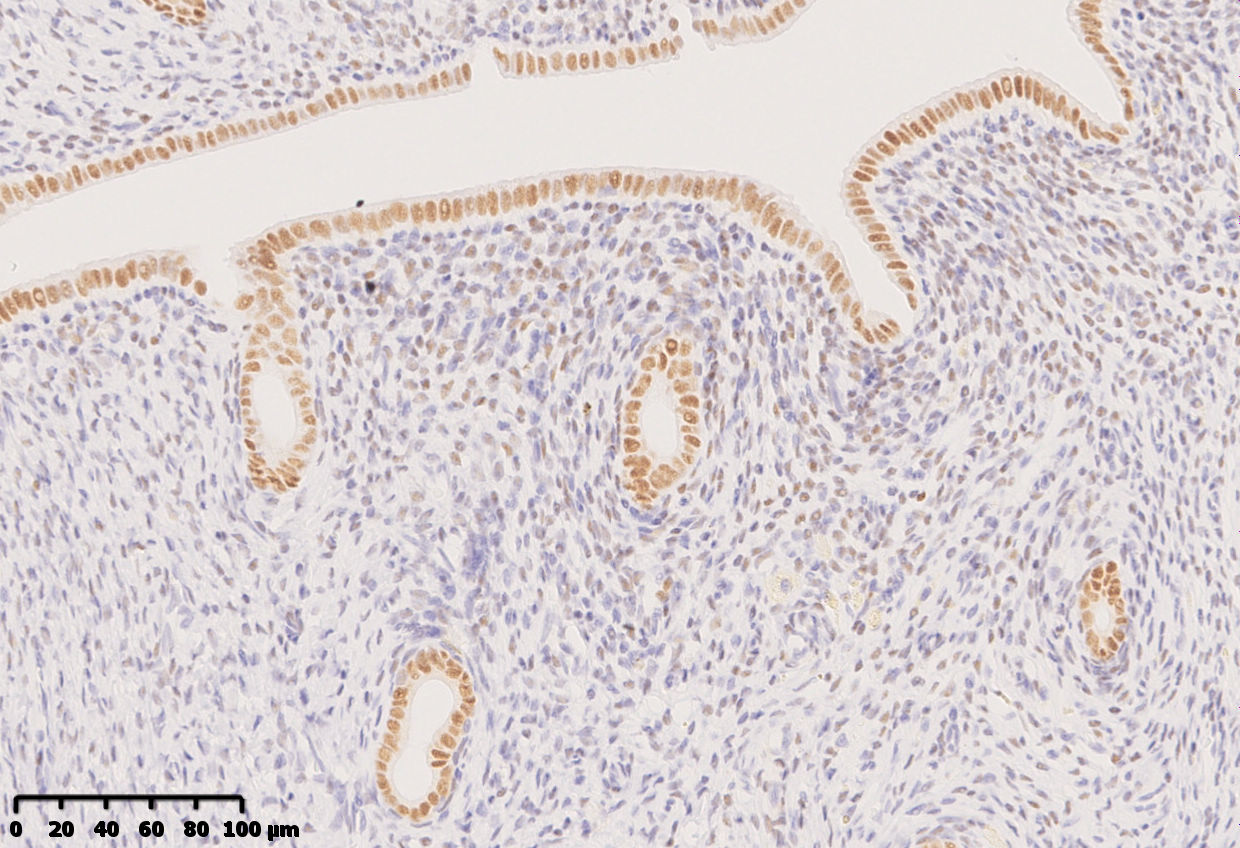

Supplement: Supplementary file 1 [file datasheet1.zip › ╣Γ├▄╢╚╔¿├Φ_final/ERA/ERA_HPL-1.jpg]

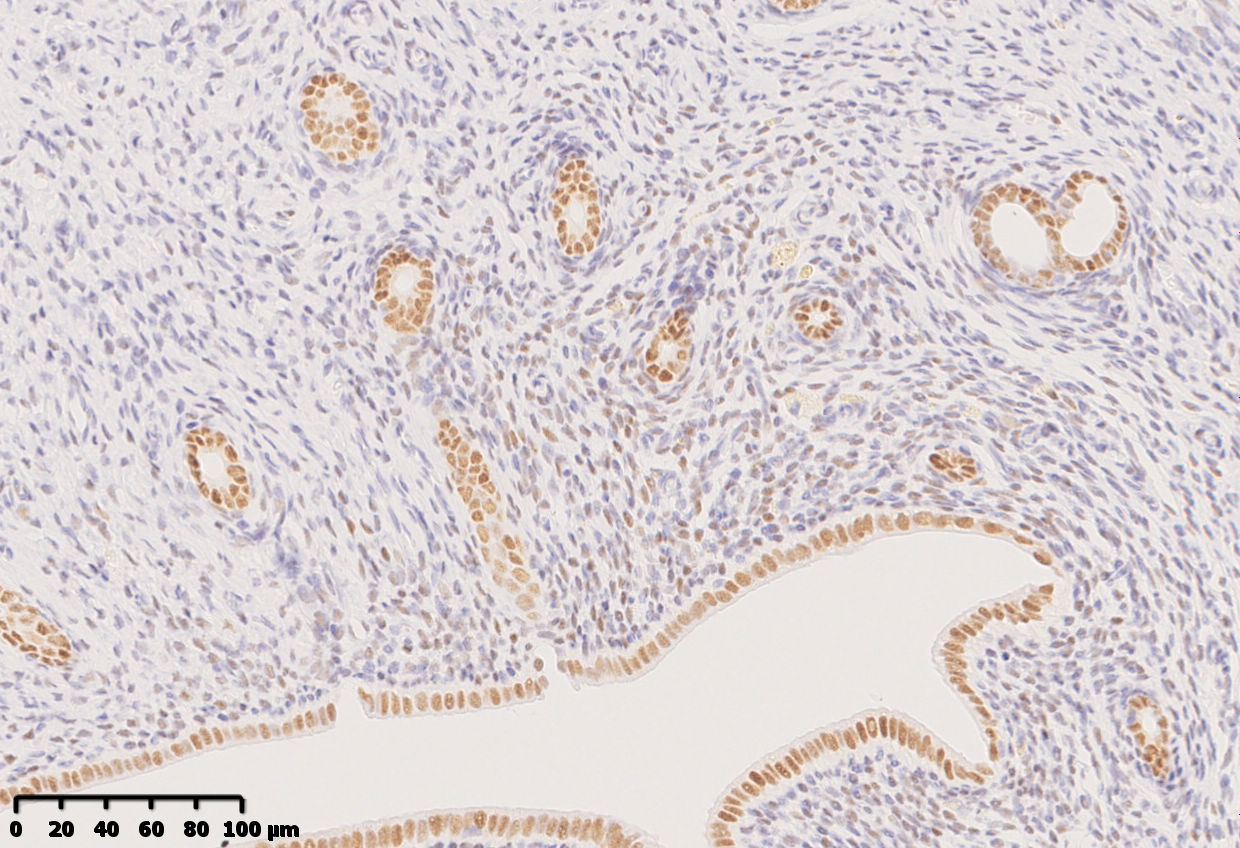

Supplement: Supplementary file 1 [file datasheet1.zip › ╣Γ├▄╢╚╔¿├Φ_final/ERA/ERA_HPL-2.jpg]

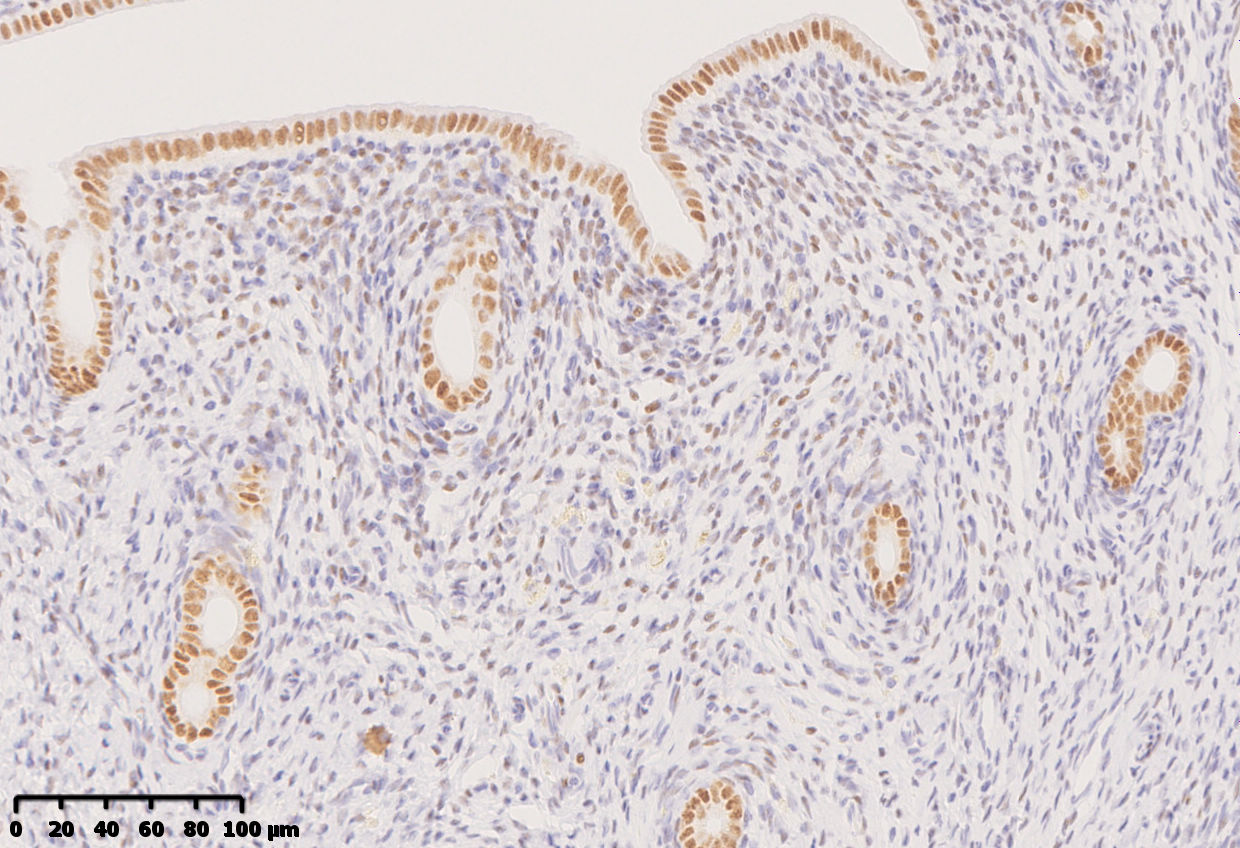

Supplement: Supplementary file 1 [file datasheet1.zip › ╣Γ├▄╢╚╔¿├Φ_final/ERA/ERA_HPL-3.jpg]

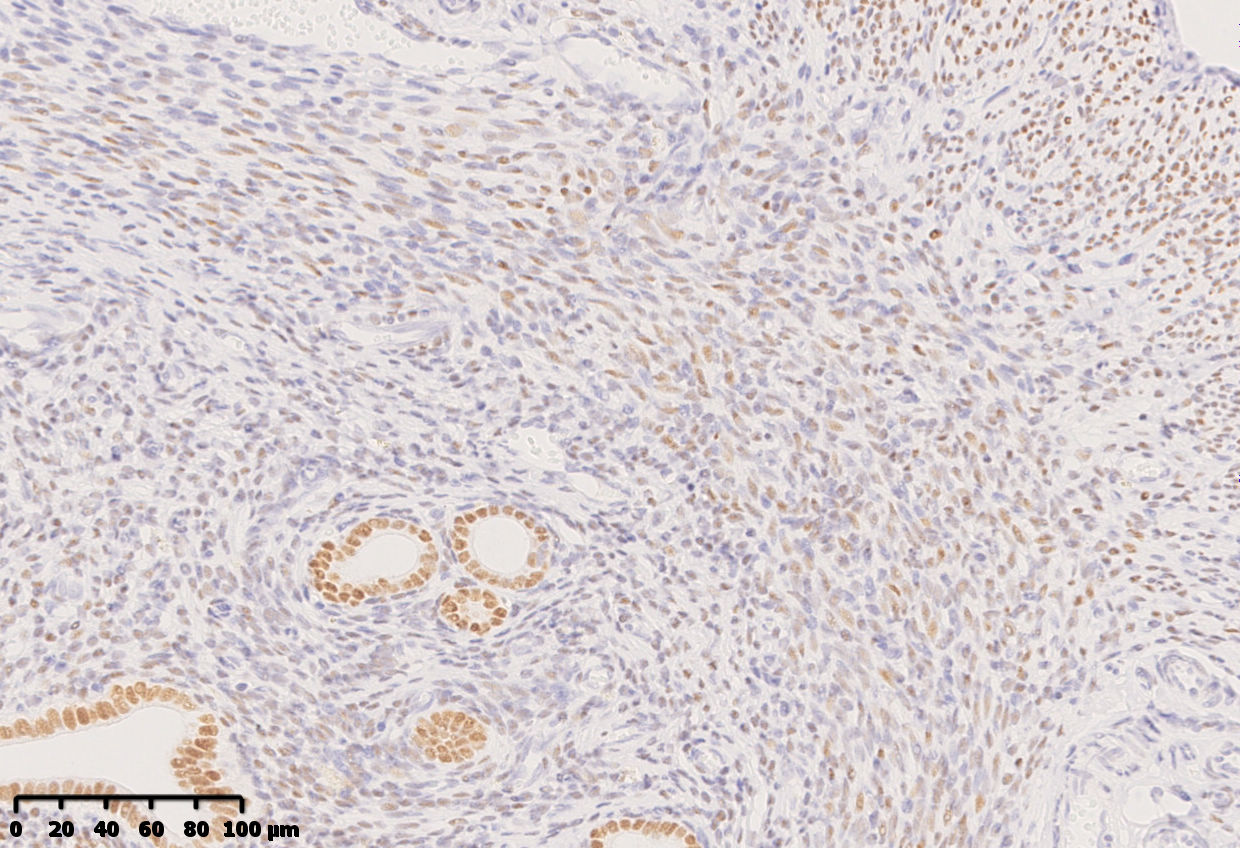

Supplement: Supplementary file 1 [file datasheet1.zip › ╣Γ├▄╢╚╔¿├Φ_final/ERA/ERA_OVX-1.jpg]

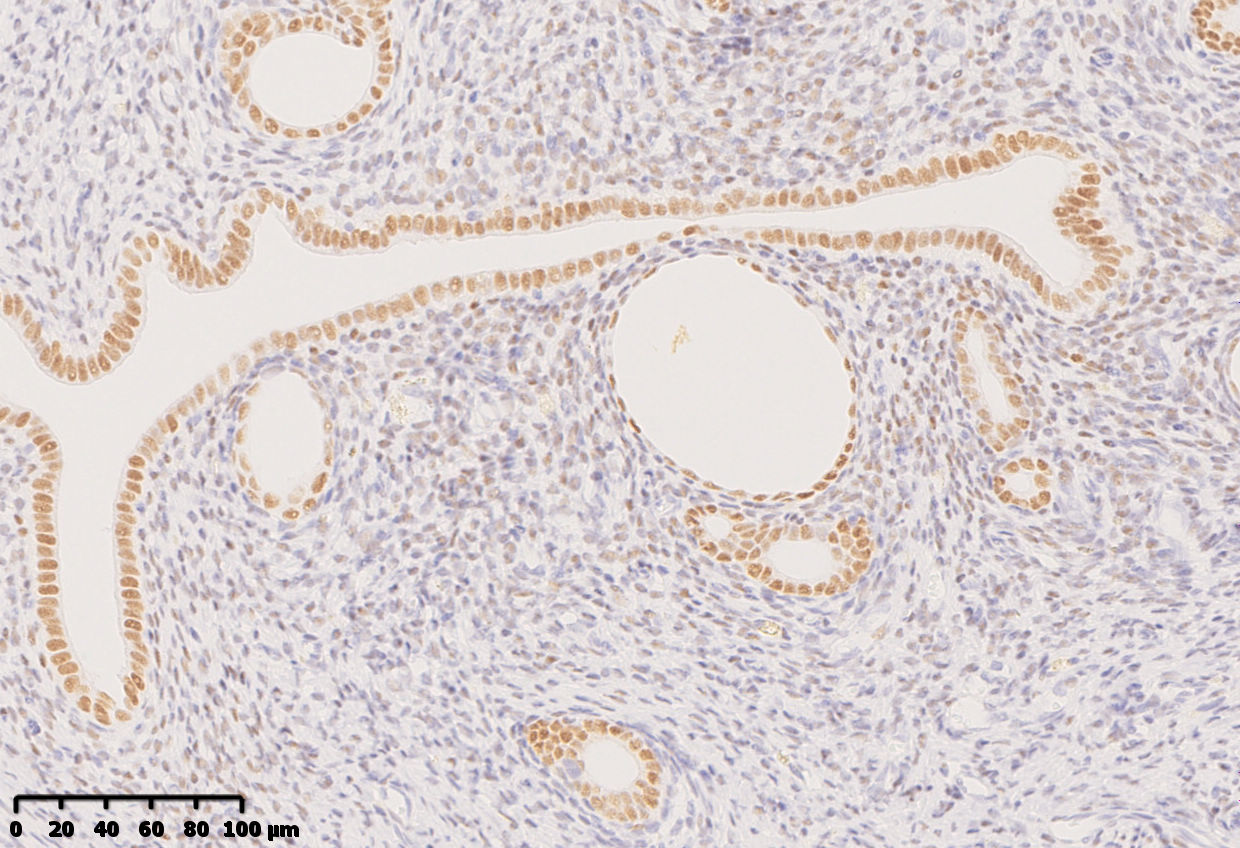

Supplement: Supplementary file 1 [file datasheet1.zip › ╣Γ├▄╢╚╔¿├Φ_final/ERA/ERA_OVX-2.jpg]

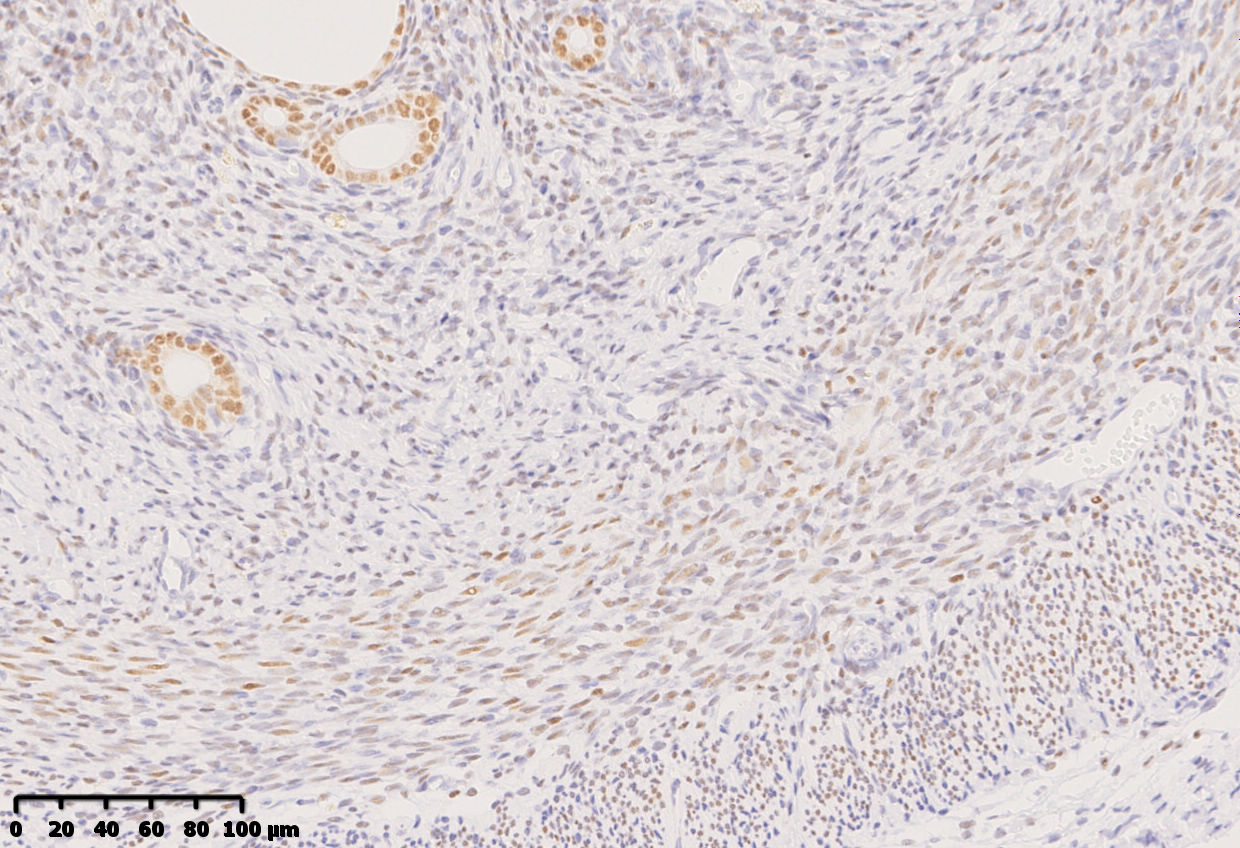

Supplement: Supplementary file 1 [file datasheet1.zip › ╣Γ├▄╢╚╔¿├Φ_final/ERA/ERA_OVX-3.jpg]

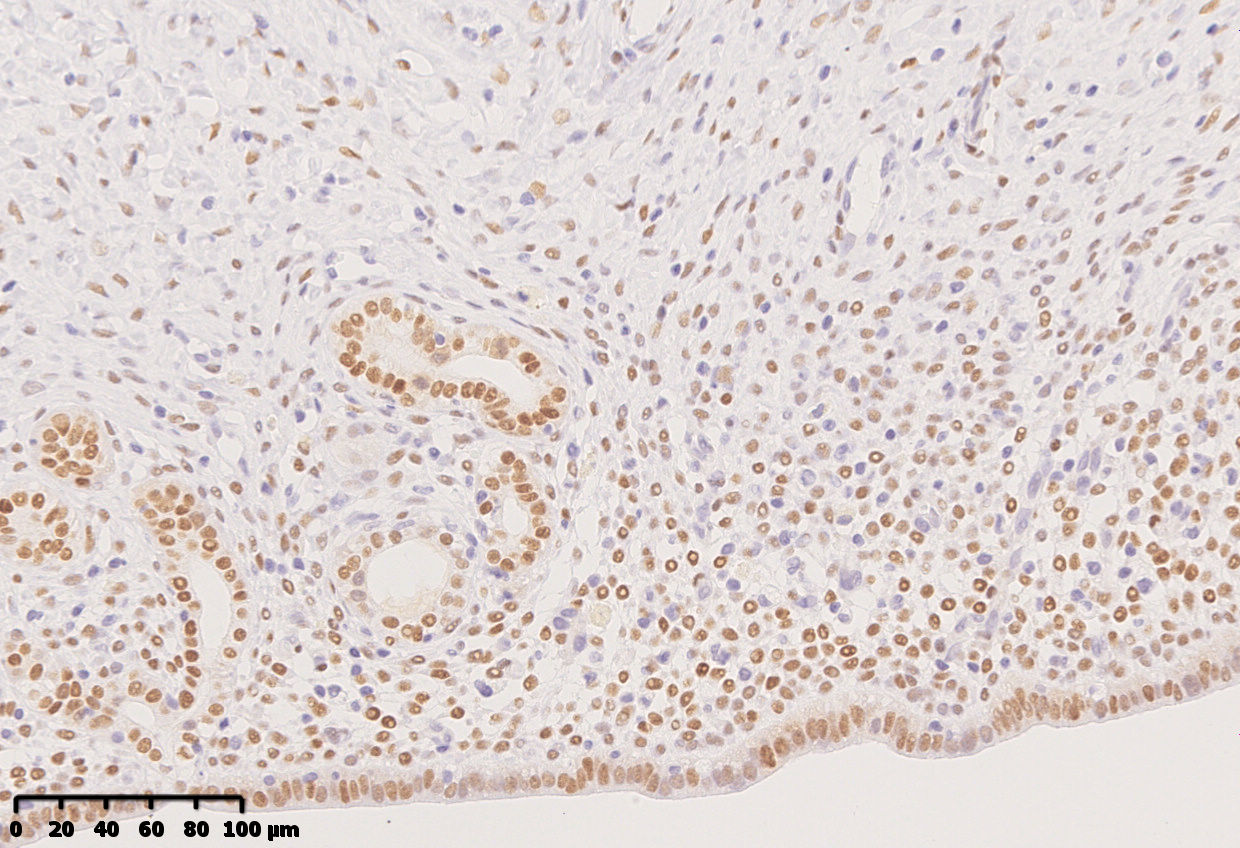

Supplement: Supplementary file 1 [file datasheet1.zip › ╣Γ├▄╢╚╔¿├Φ_final/ERA/ERA_con-1.jpg]

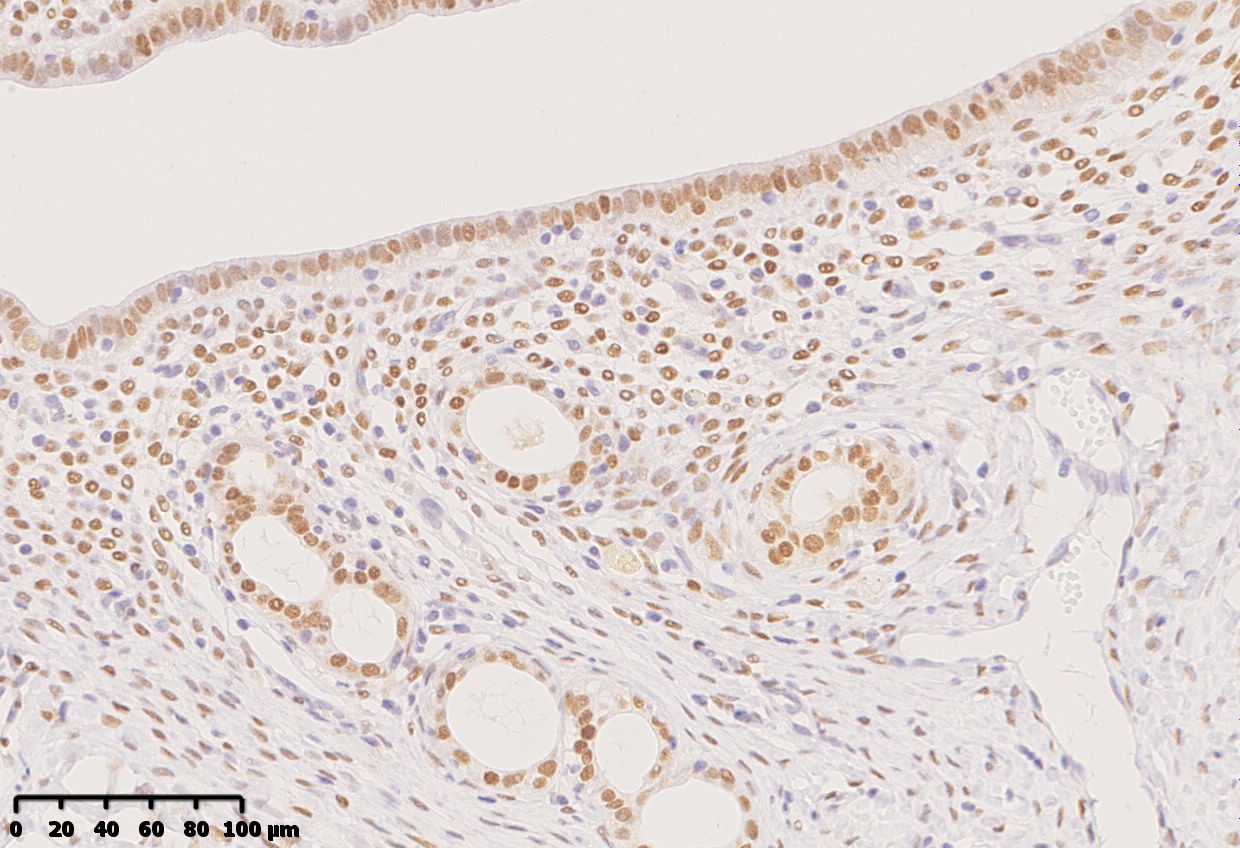

Supplement: Supplementary file 1 [file datasheet1.zip › ╣Γ├▄╢╚╔¿├Φ_final/ERA/ERA_con-2.jpg]

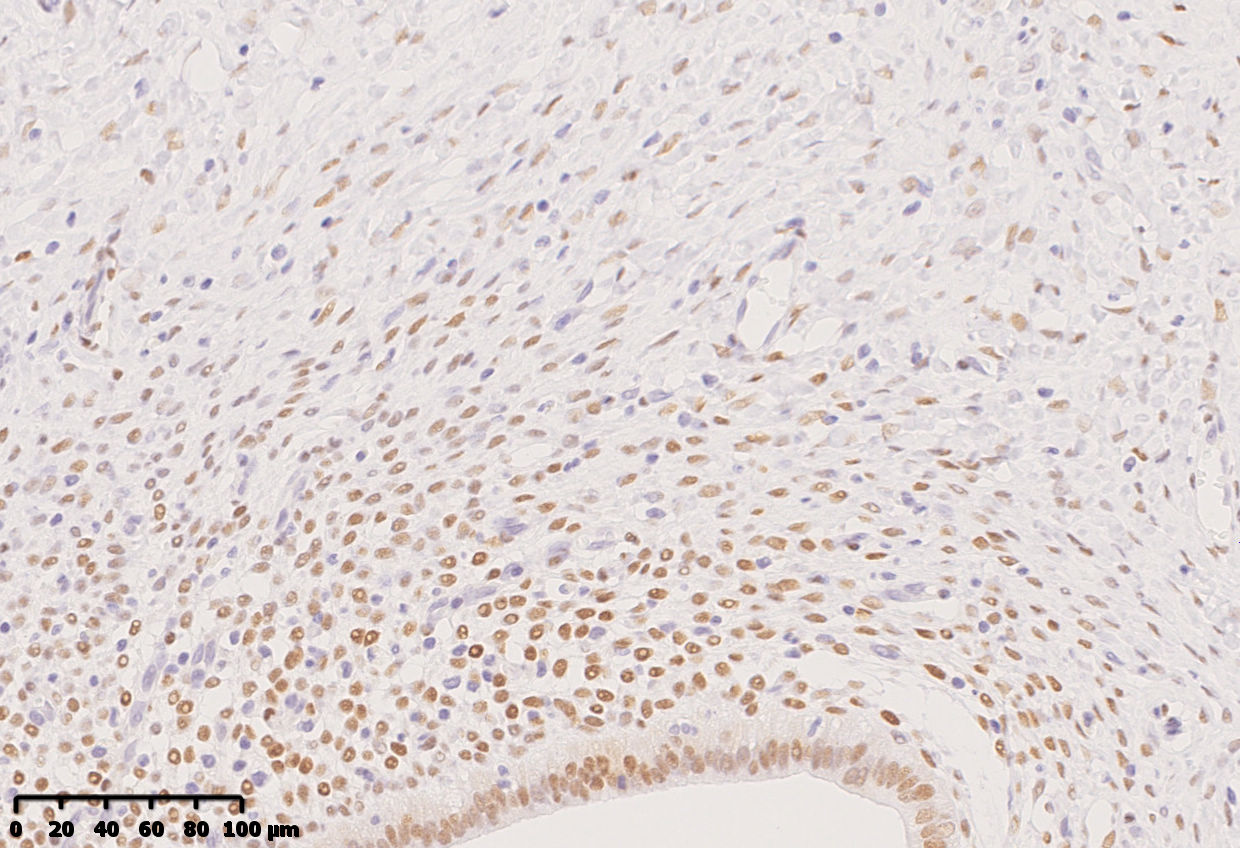

Supplement: Supplementary file 1 [file datasheet1.zip › ╣Γ├▄╢╚╔¿├Φ_final/ERA/ERA_con-3.jpg]

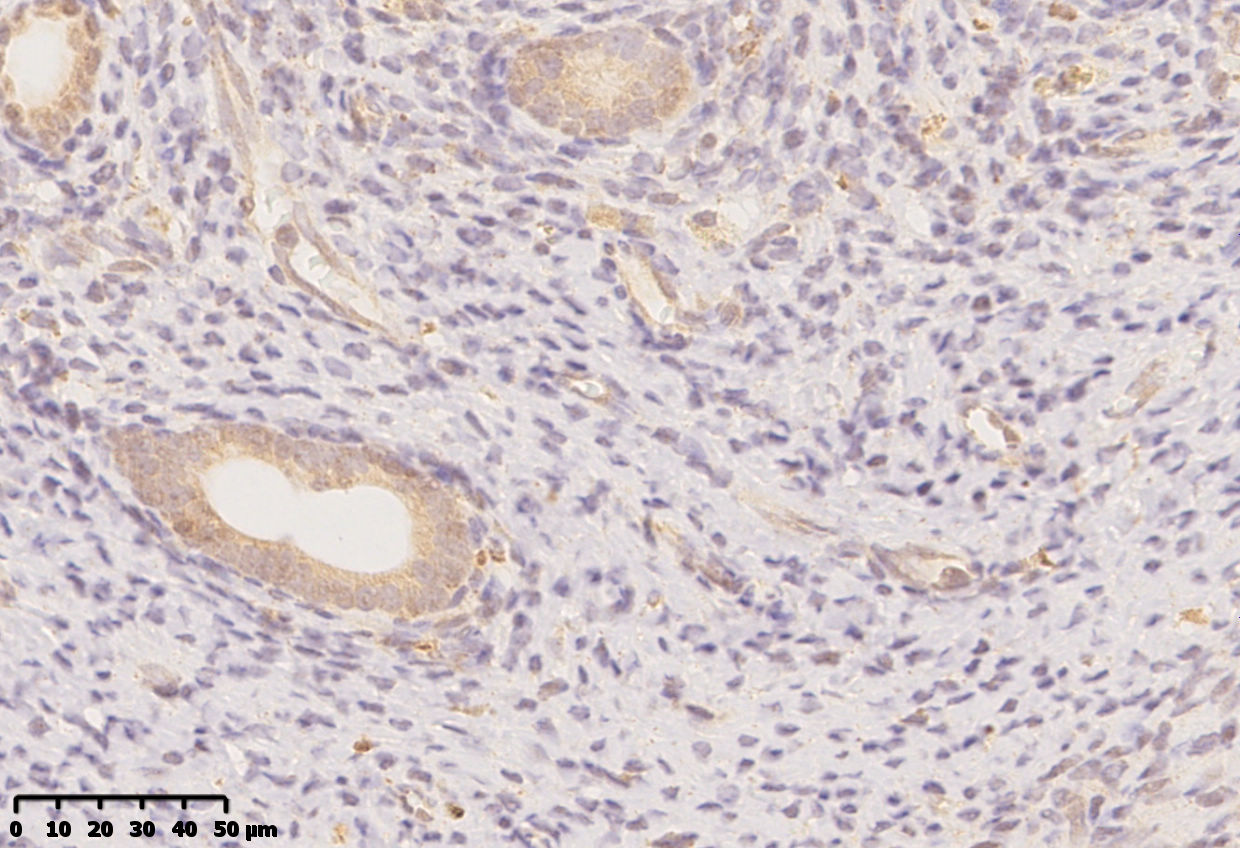

Supplement: Supplementary file 1 [file datasheet1.zip › ╣Γ├▄╢╚╔¿├Φ_final/ERB/ERB_HPH-1_1.jpg]

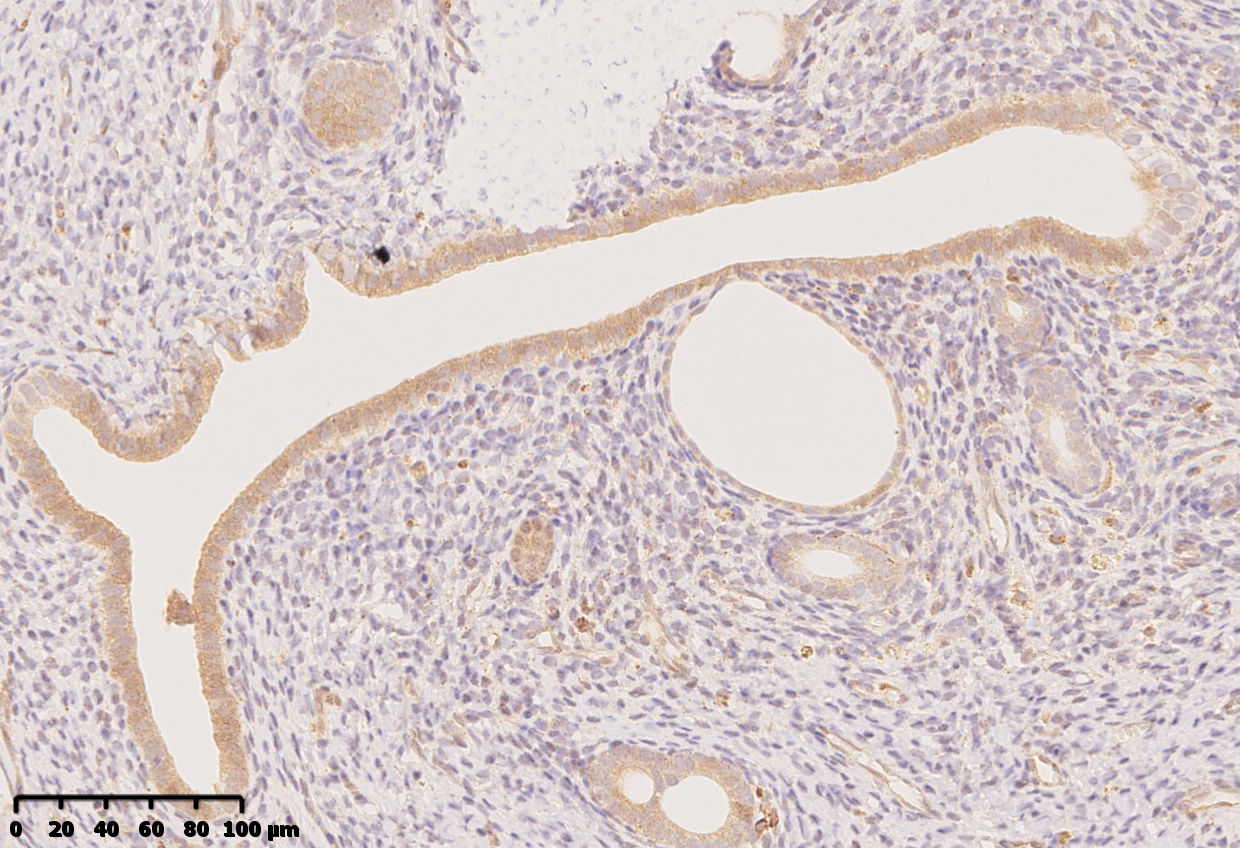

Supplement: Supplementary file 1 [file datasheet1.zip › ╣Γ├▄╢╚╔¿├Φ_final/ERB/ERB_HPH-2.jpg]

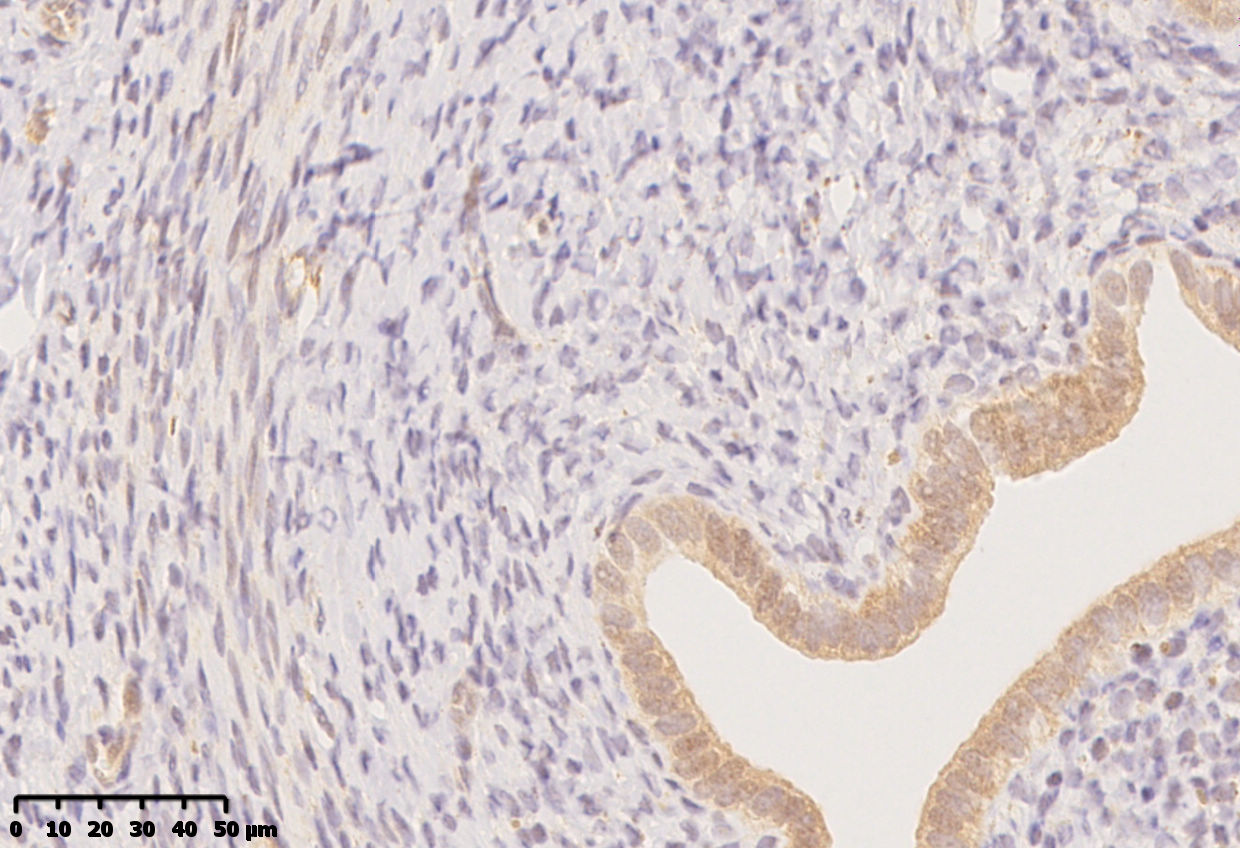

Supplement: Supplementary file 1 [file datasheet1.zip › ╣Γ├▄╢╚╔¿├Φ_final/ERB/ERB_HPH-3.jpg]

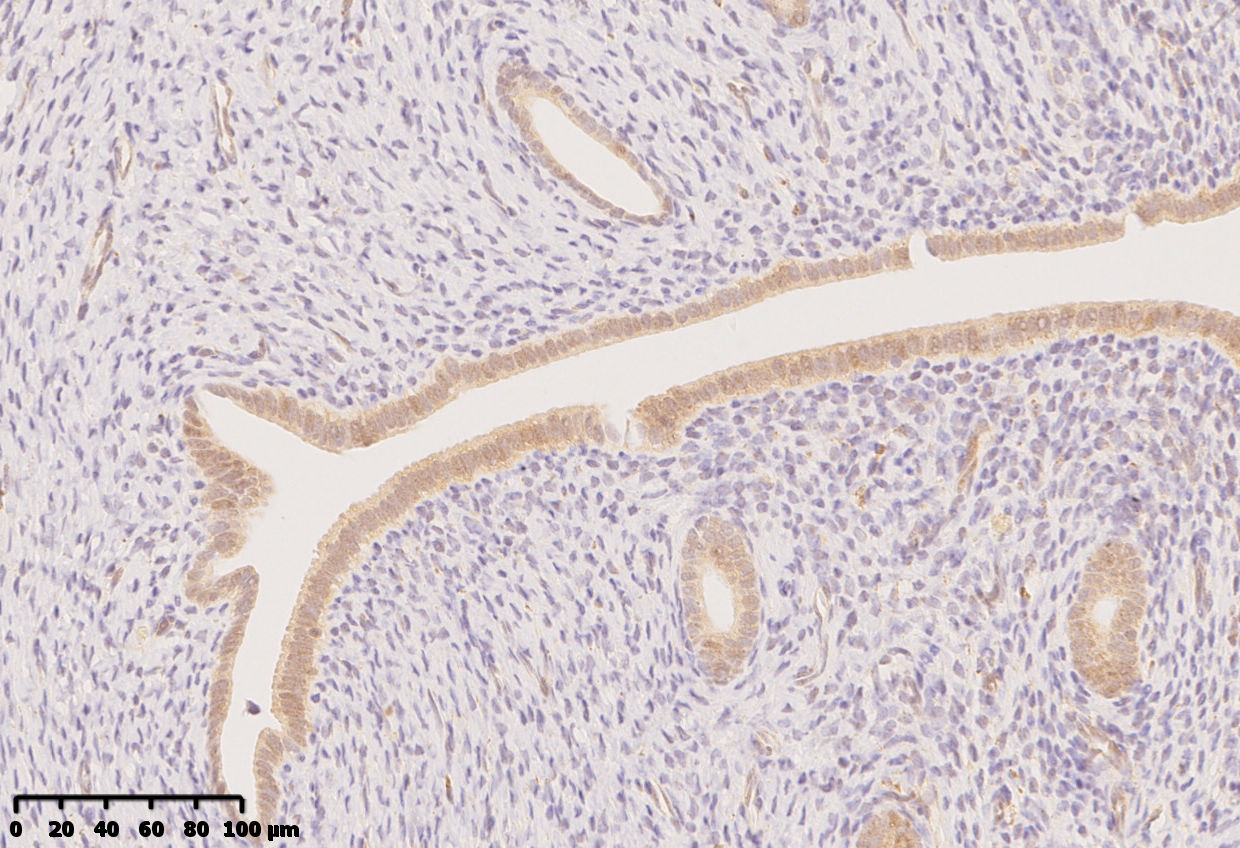

Supplement: Supplementary file 1 [file datasheet1.zip › ╣Γ├▄╢╚╔¿├Φ_final/ERB/ERB_HPL-1.jpg]

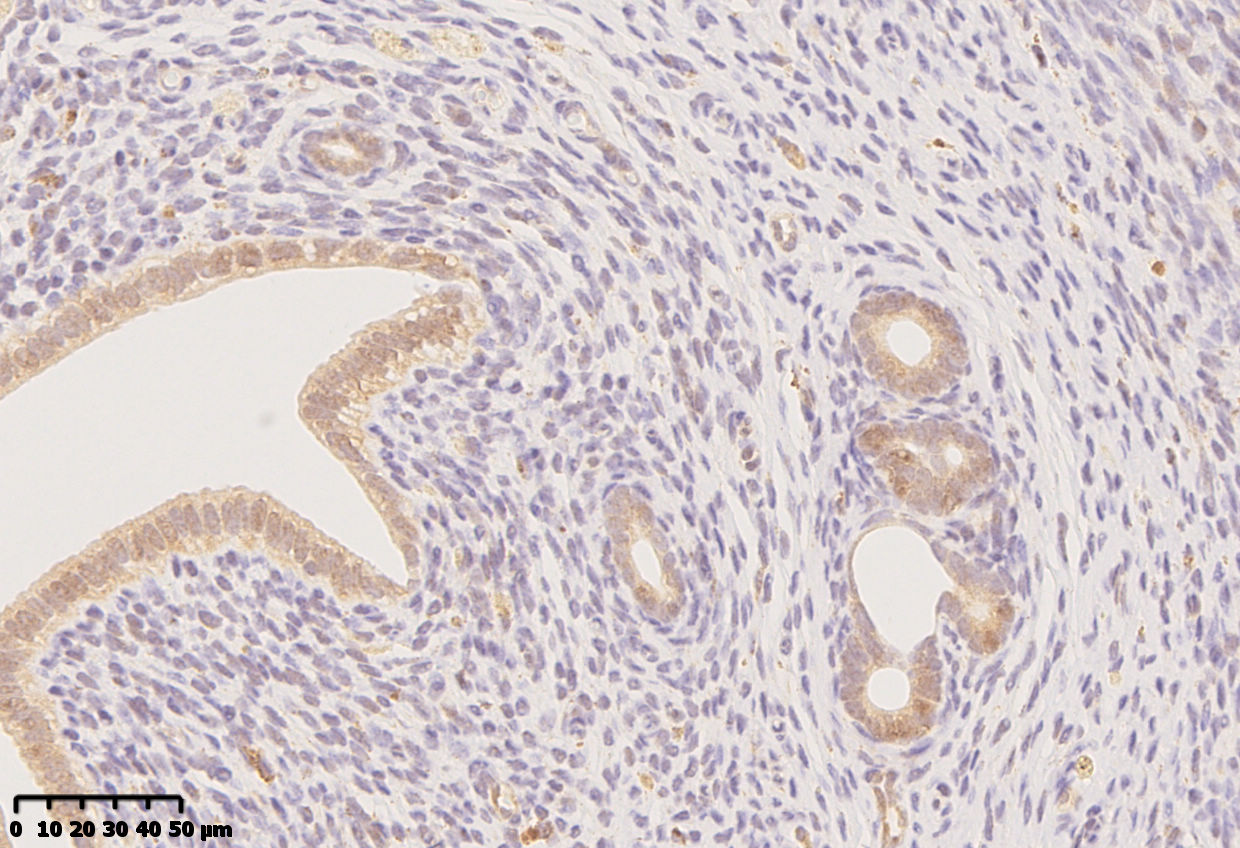

Supplement: Supplementary file 1 [file datasheet1.zip › ╣Γ├▄╢╚╔¿├Φ_final/ERB/ERB_HPL-2.jpg]

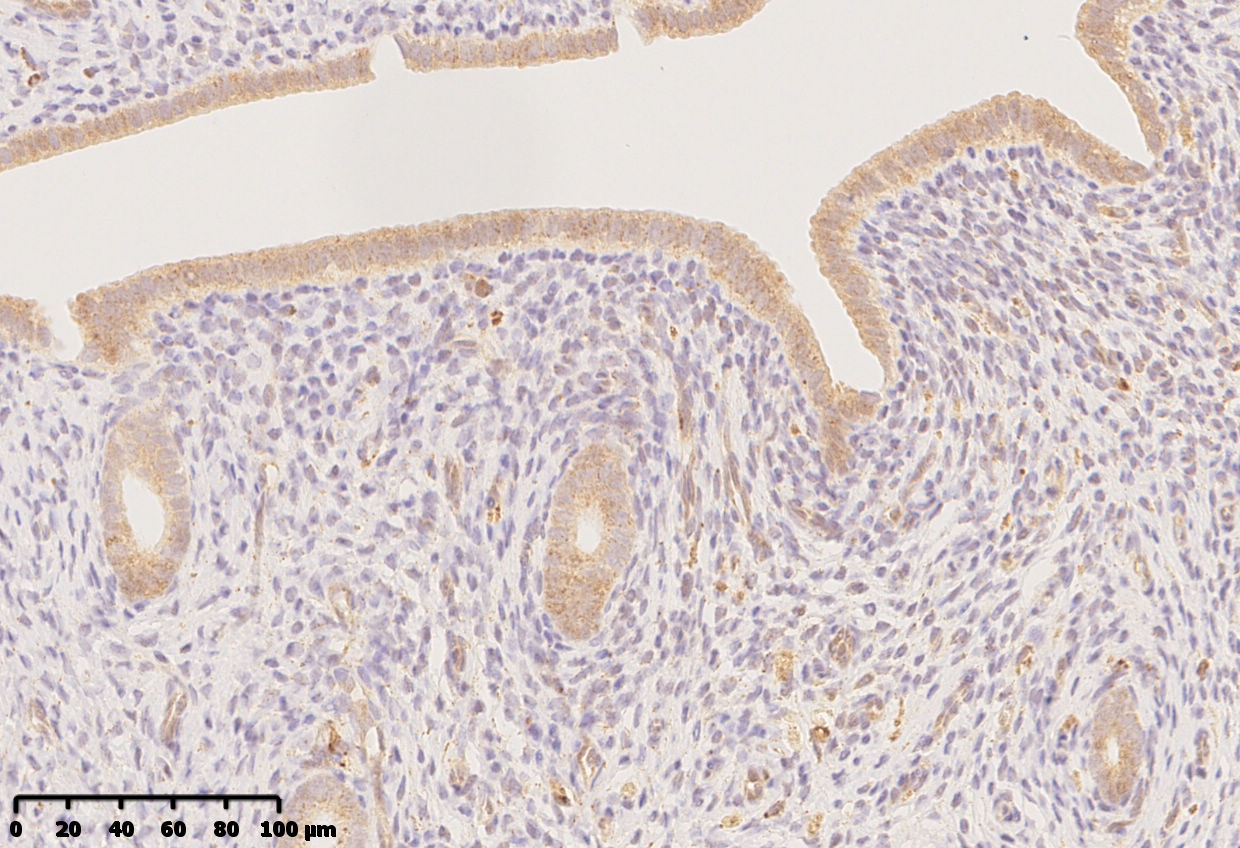

Supplement: Supplementary file 1 [file datasheet1.zip › ╣Γ├▄╢╚╔¿├Φ_final/ERB/ERB_HPL-3.jpg]

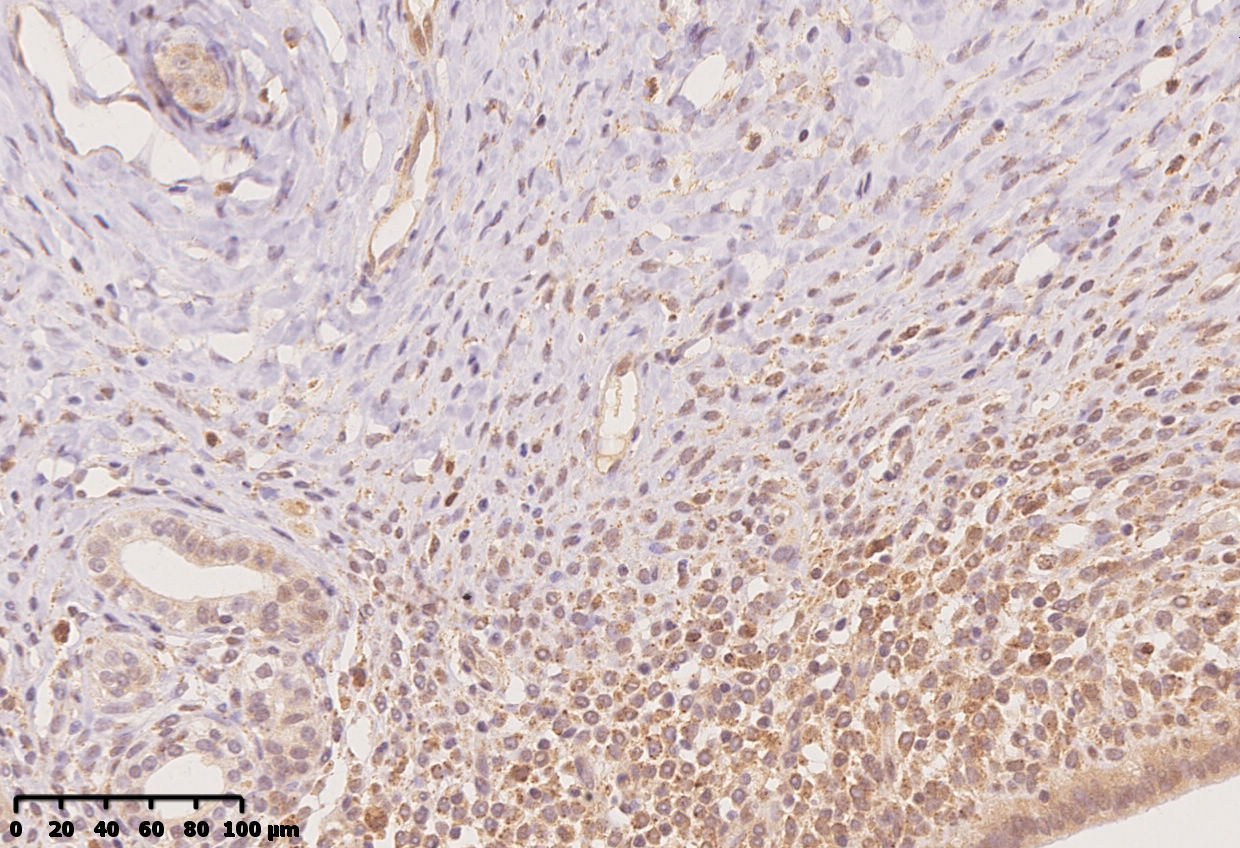

Supplement: Supplementary file 1 [file datasheet1.zip › ╣Γ├▄╢╚╔¿├Φ_final/ERB/ERB_con-1.jpg]

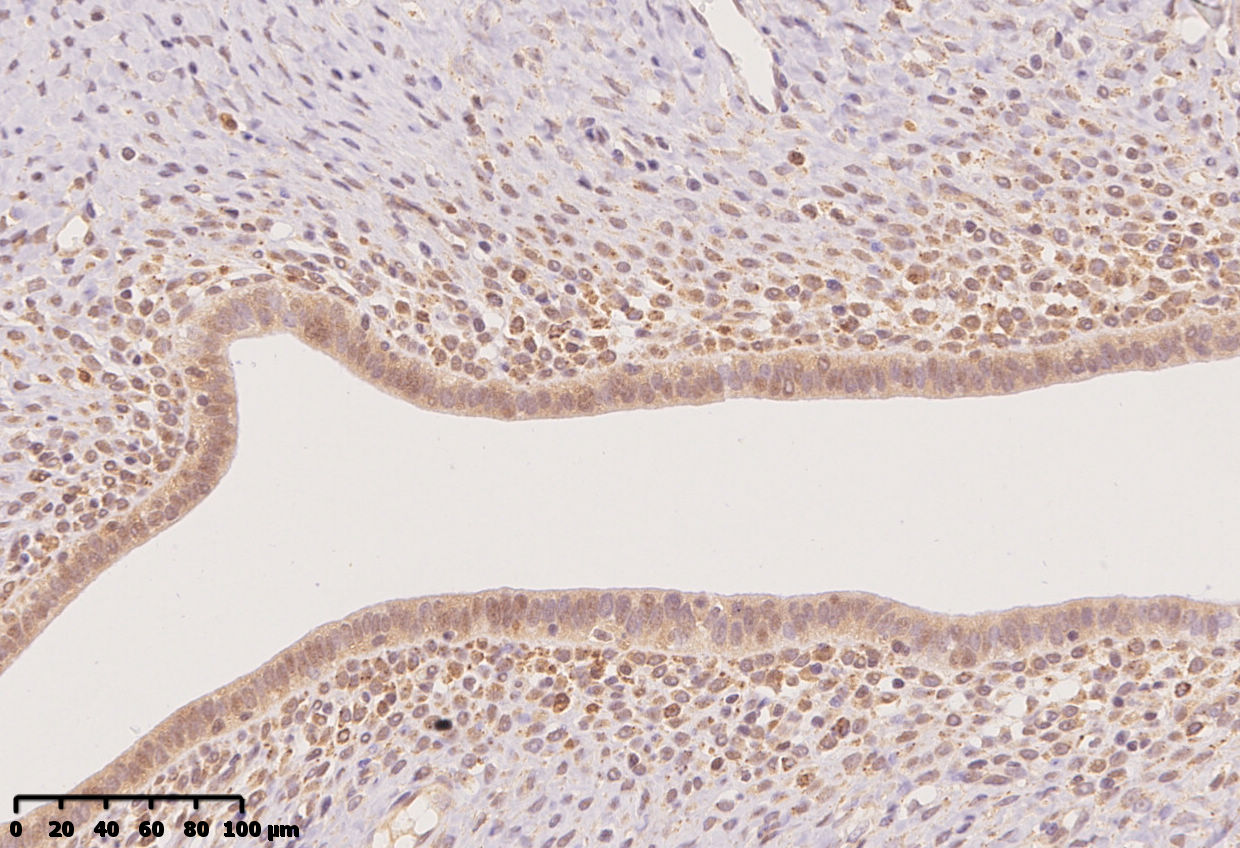

Supplement: Supplementary file 1 [file datasheet1.zip › ╣Γ├▄╢╚╔¿├Φ_final/ERB/ERB_con-2.jpg]

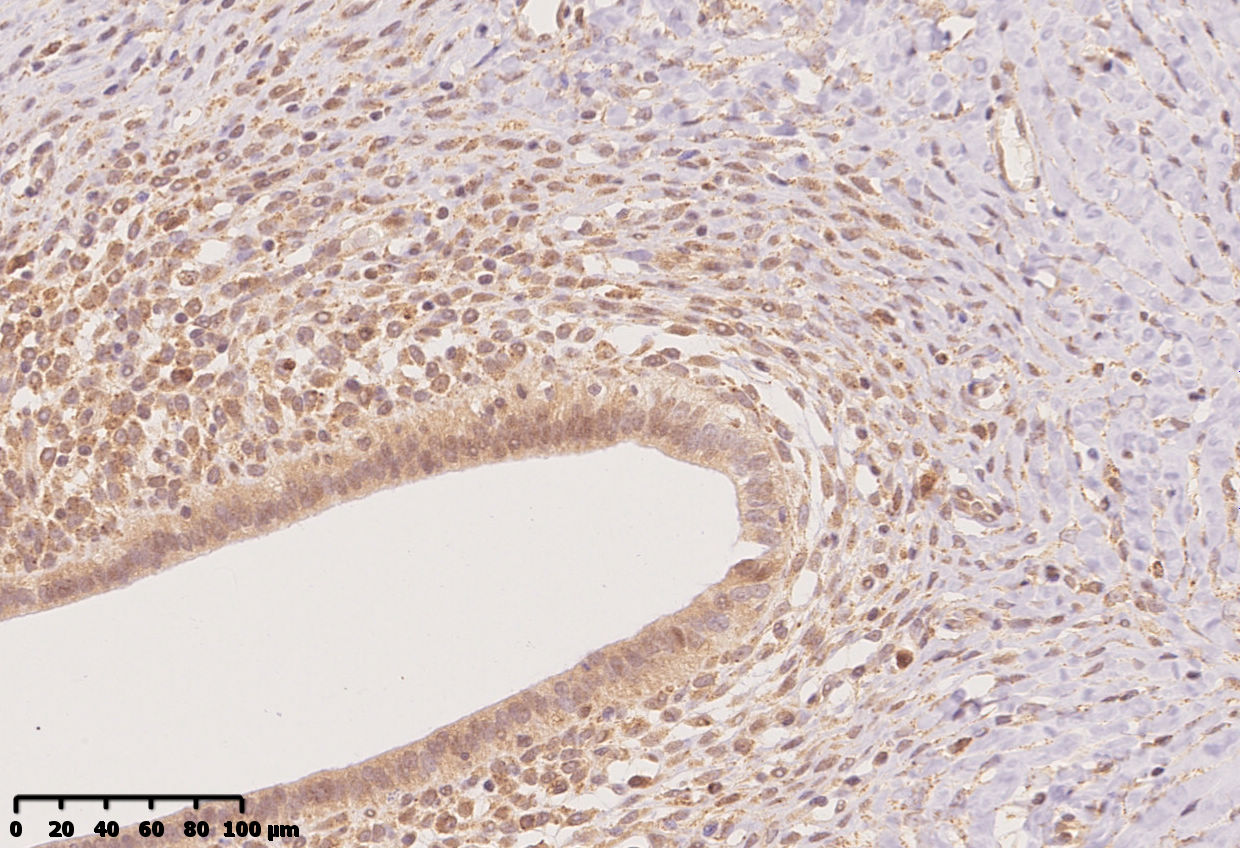

Supplement: Supplementary file 1 [file datasheet1.zip › ╣Γ├▄╢╚╔¿├Φ_final/ERB/ERB_con-3.jpg]

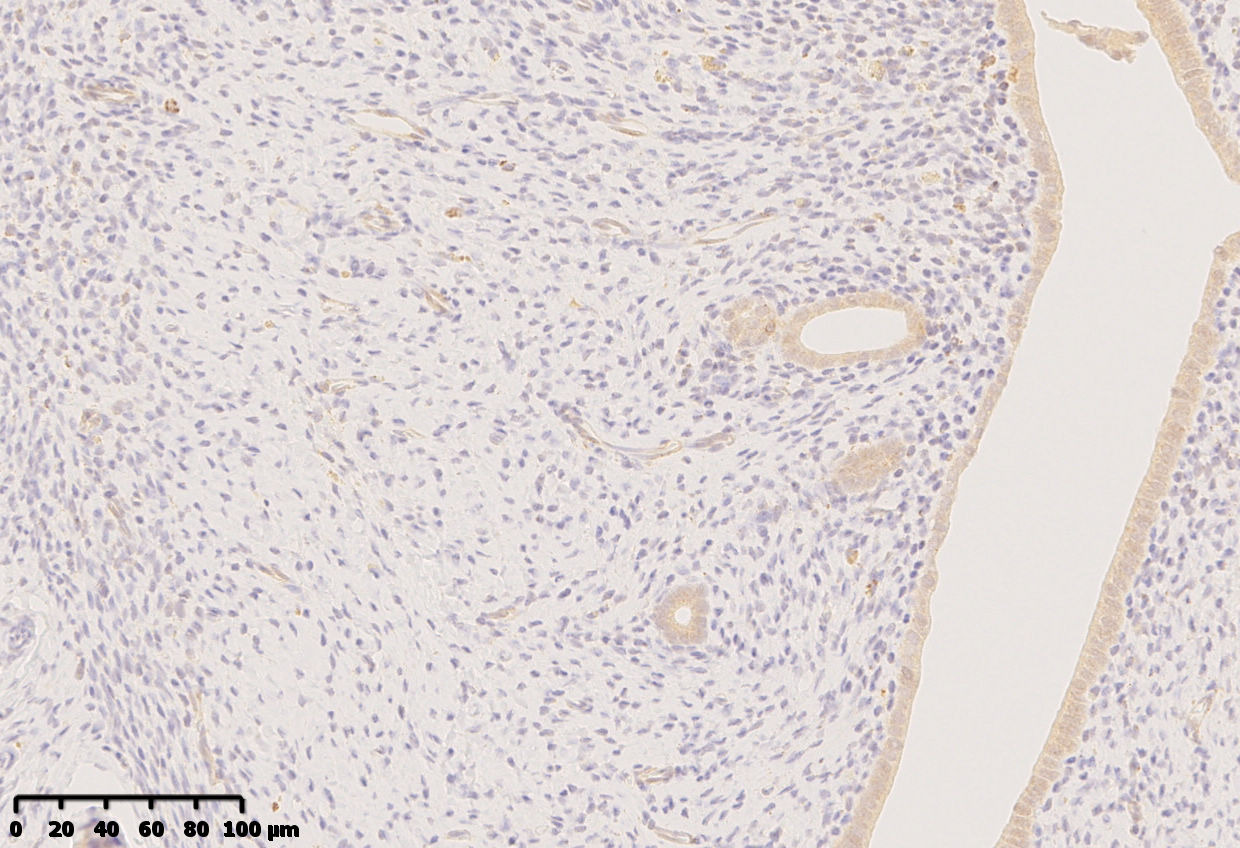

Supplement: Supplementary file 1 [file datasheet1.zip › ╣Γ├▄╢╚╔¿├Φ_final/ERB/ERB_ovx-1.jpg]

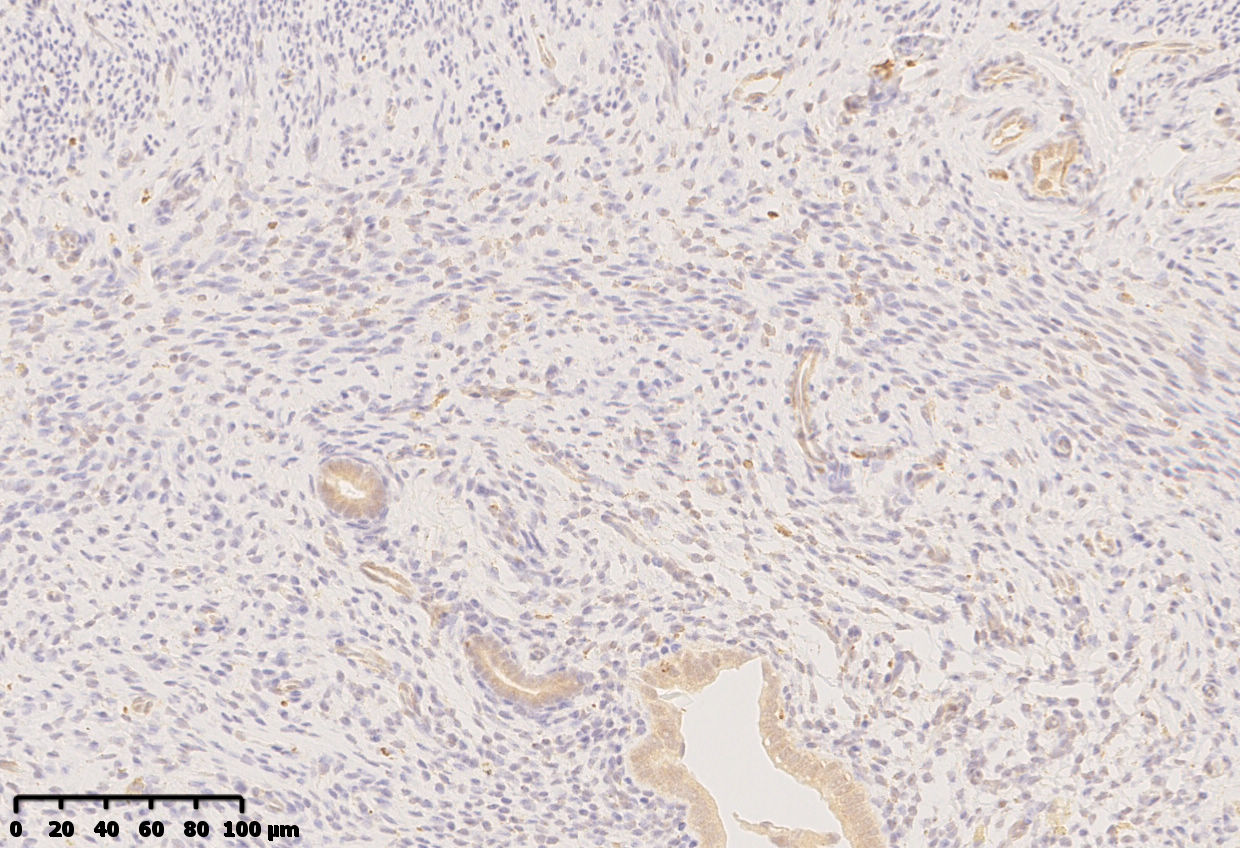

Supplement: Supplementary file 1 [file datasheet1.zip › ╣Γ├▄╢╚╔¿├Φ_final/ERB/ERB_ovx-2.jpg]

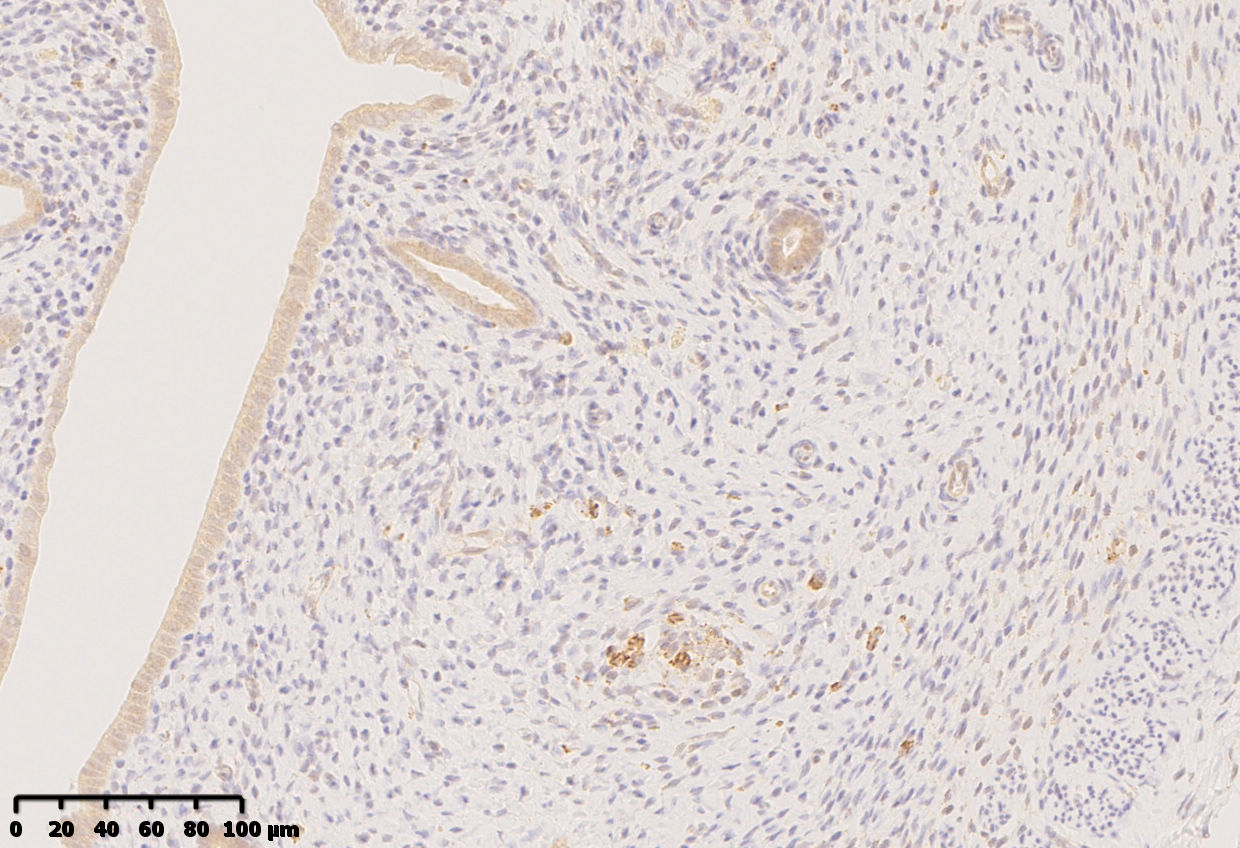

Supplement: Supplementary file 1 [file datasheet1.zip › ╣Γ├▄╢╚╔¿├Φ_final/ERB/ERB_ovx-3.jpg]

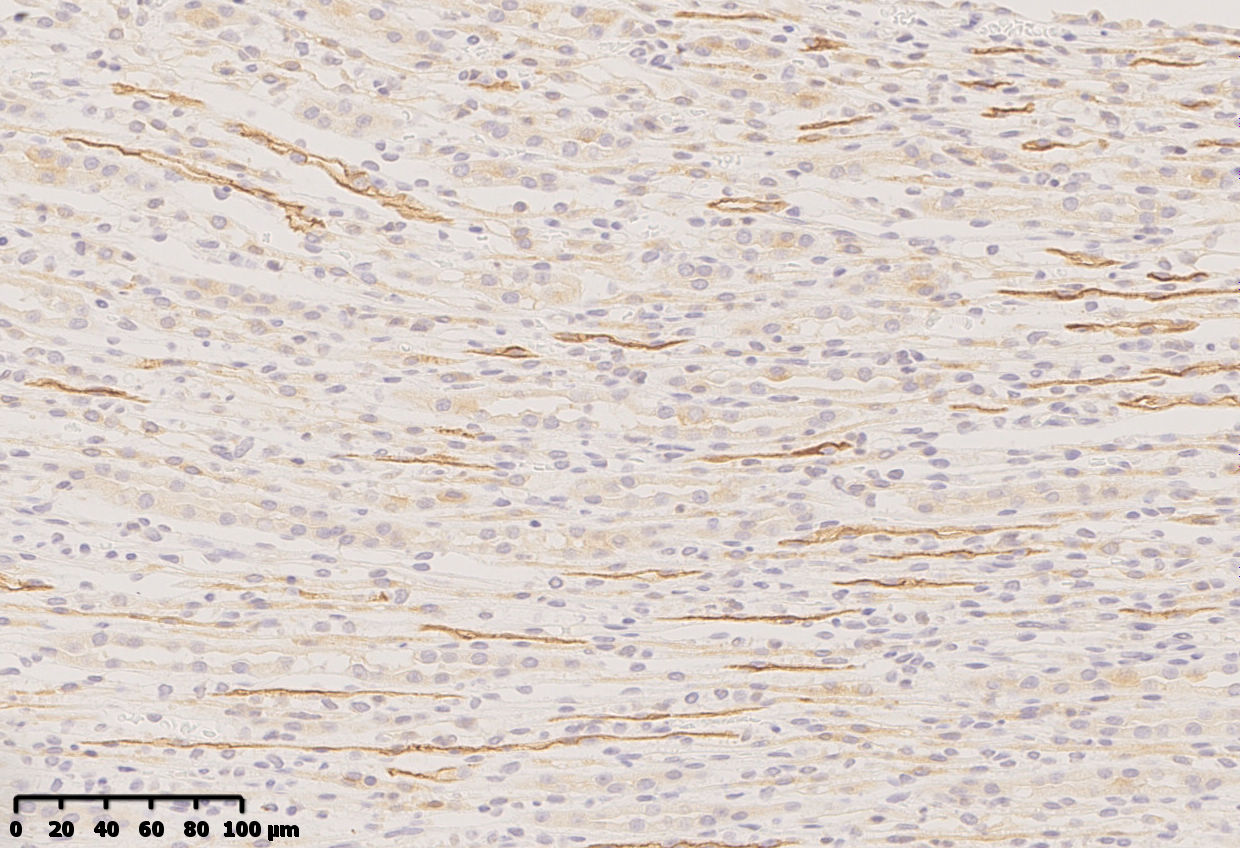

Supplement: Supplementary file 1 [file datasheet1.zip › ╣Γ├▄╢╚╔¿├Φ_final/GGT/ggt_HPH-1.jpg]

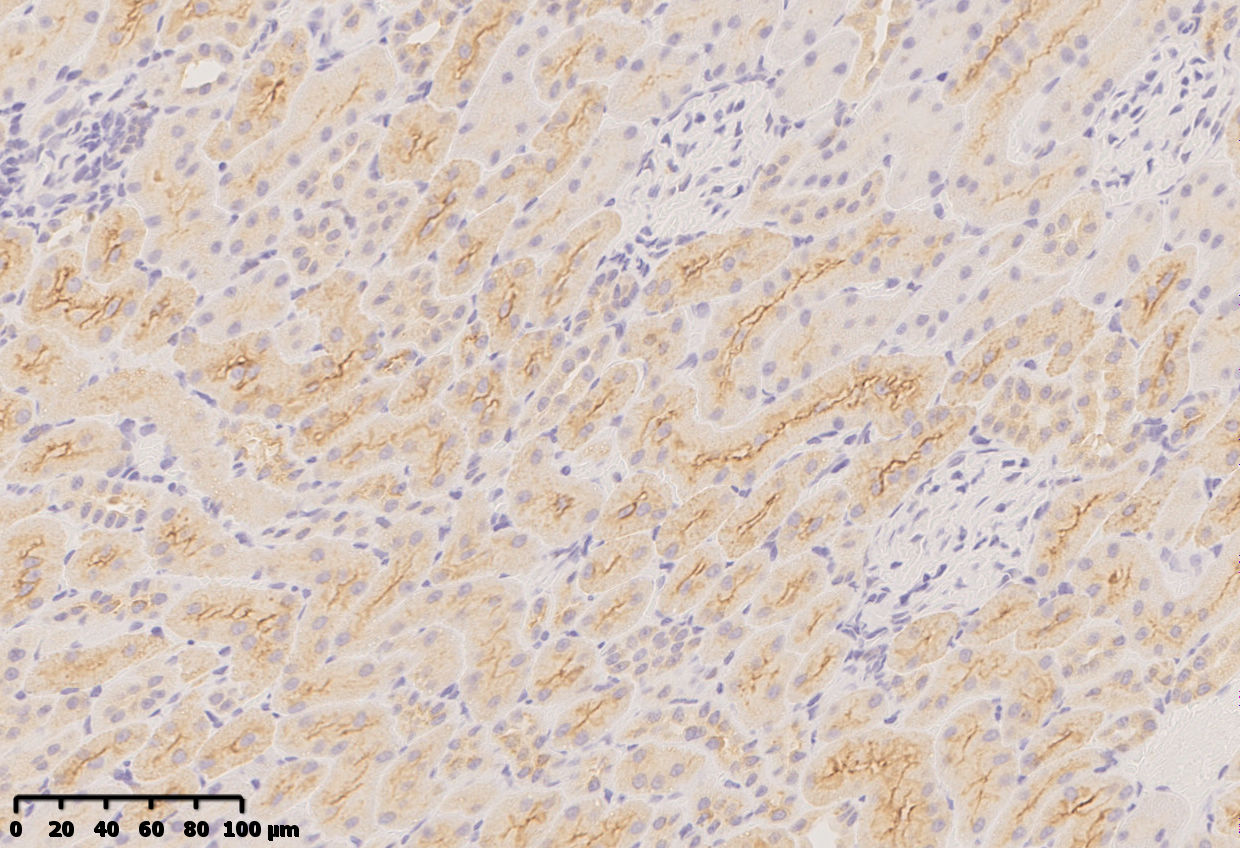

Supplement: Supplementary file 1 [file datasheet1.zip › ╣Γ├▄╢╚╔¿├Φ_final/GGT/ggt_HPH-2.jpg]

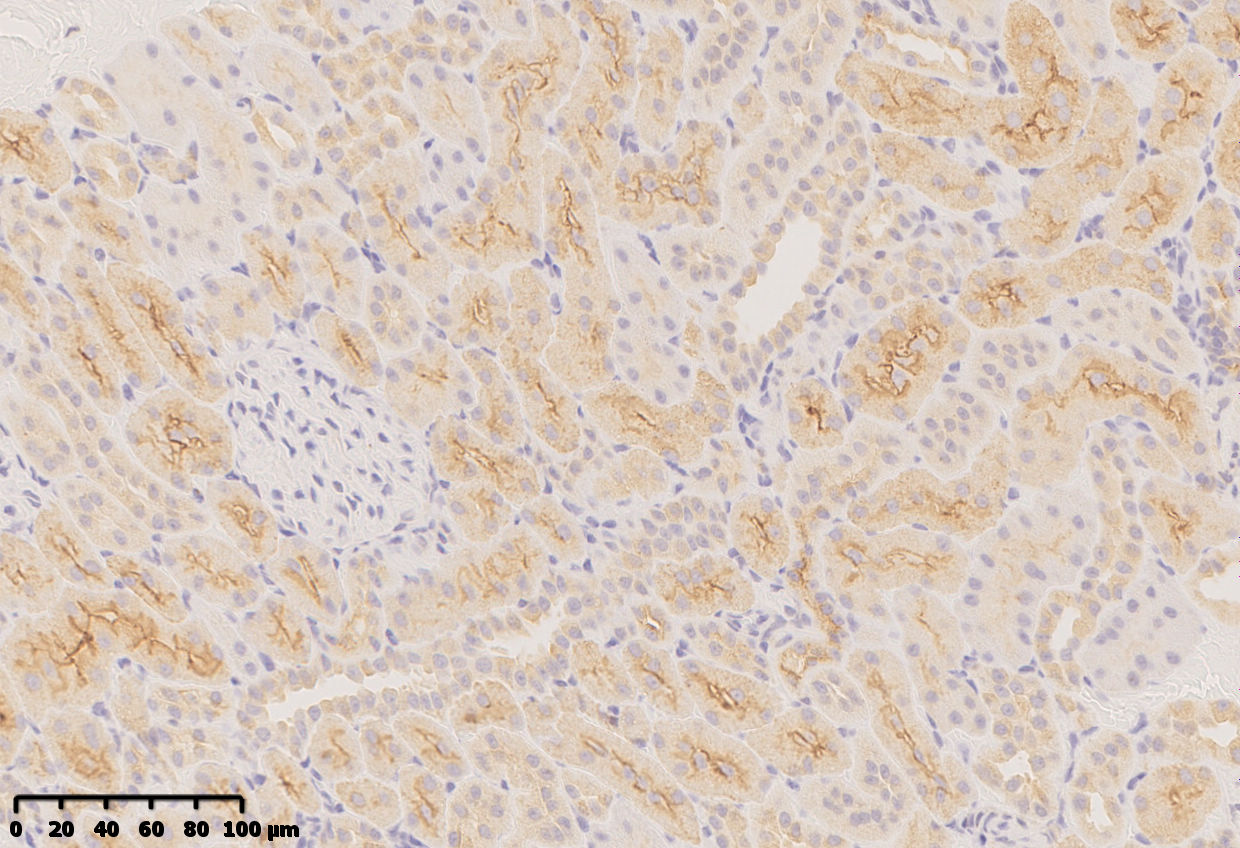

Supplement: Supplementary file 1 [file datasheet1.zip › ╣Γ├▄╢╚╔¿├Φ_final/GGT/ggt_HPH-3.jpg]

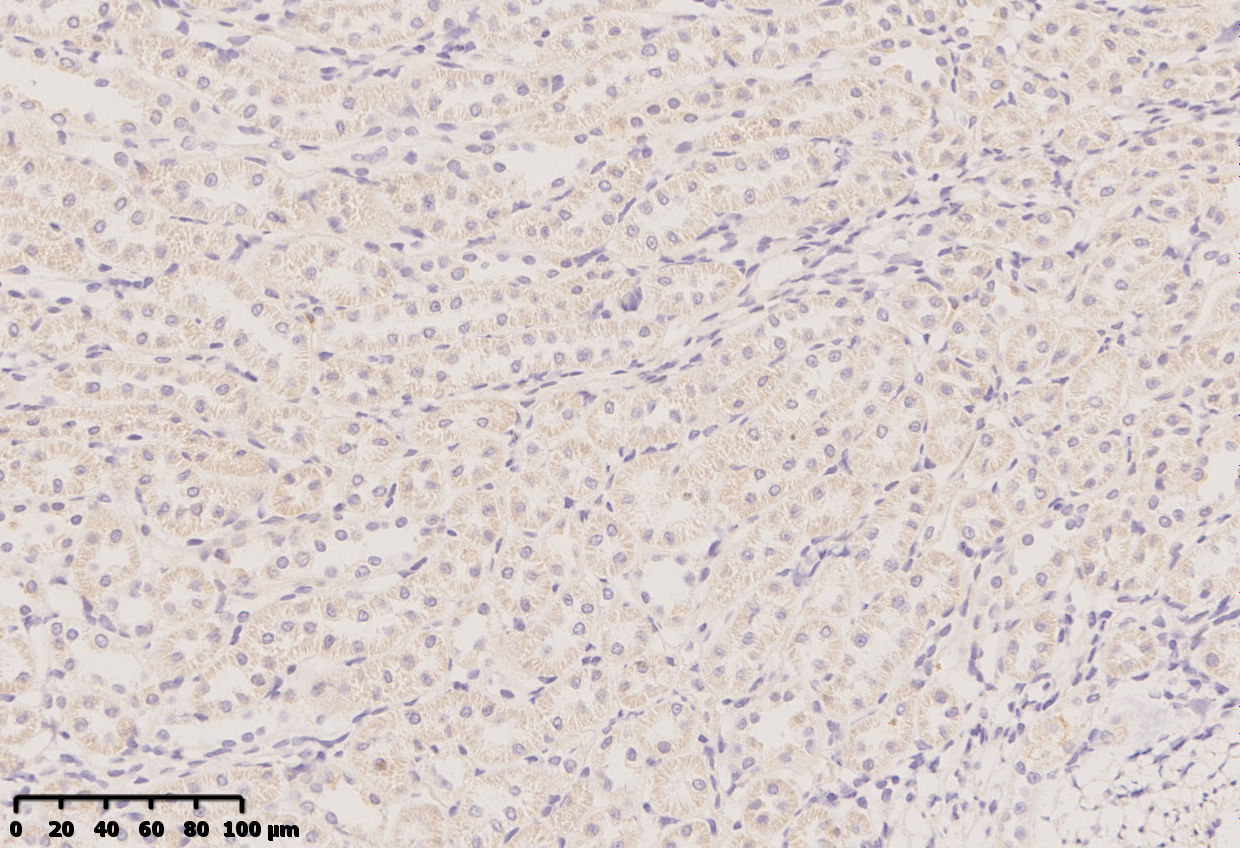

Supplement: Supplementary file 1 [file datasheet1.zip › ╣Γ├▄╢╚╔¿├Φ_final/GGT/ggt_HPL-1.jpg]

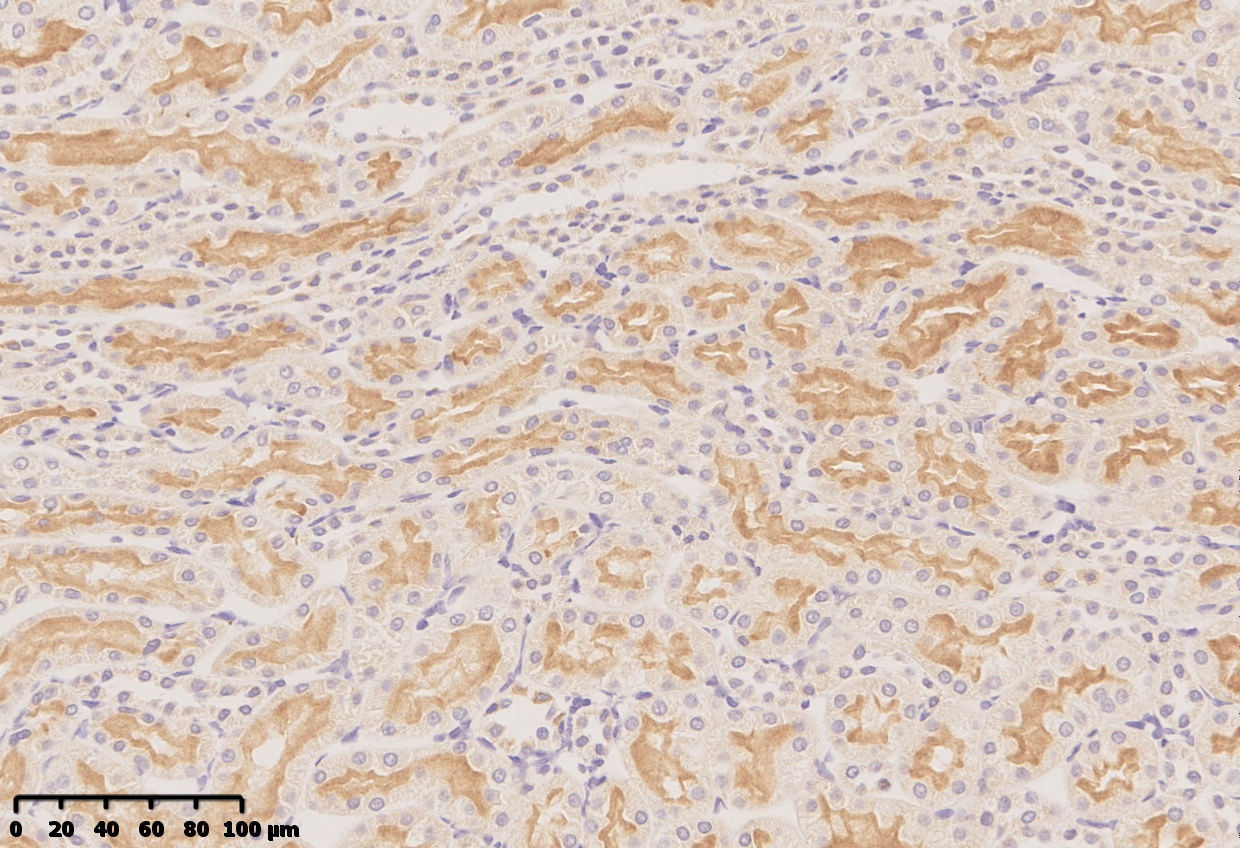

Supplement: Supplementary file 1 [file datasheet1.zip › ╣Γ├▄╢╚╔¿├Φ_final/GGT/ggt_HPL-2.jpg]

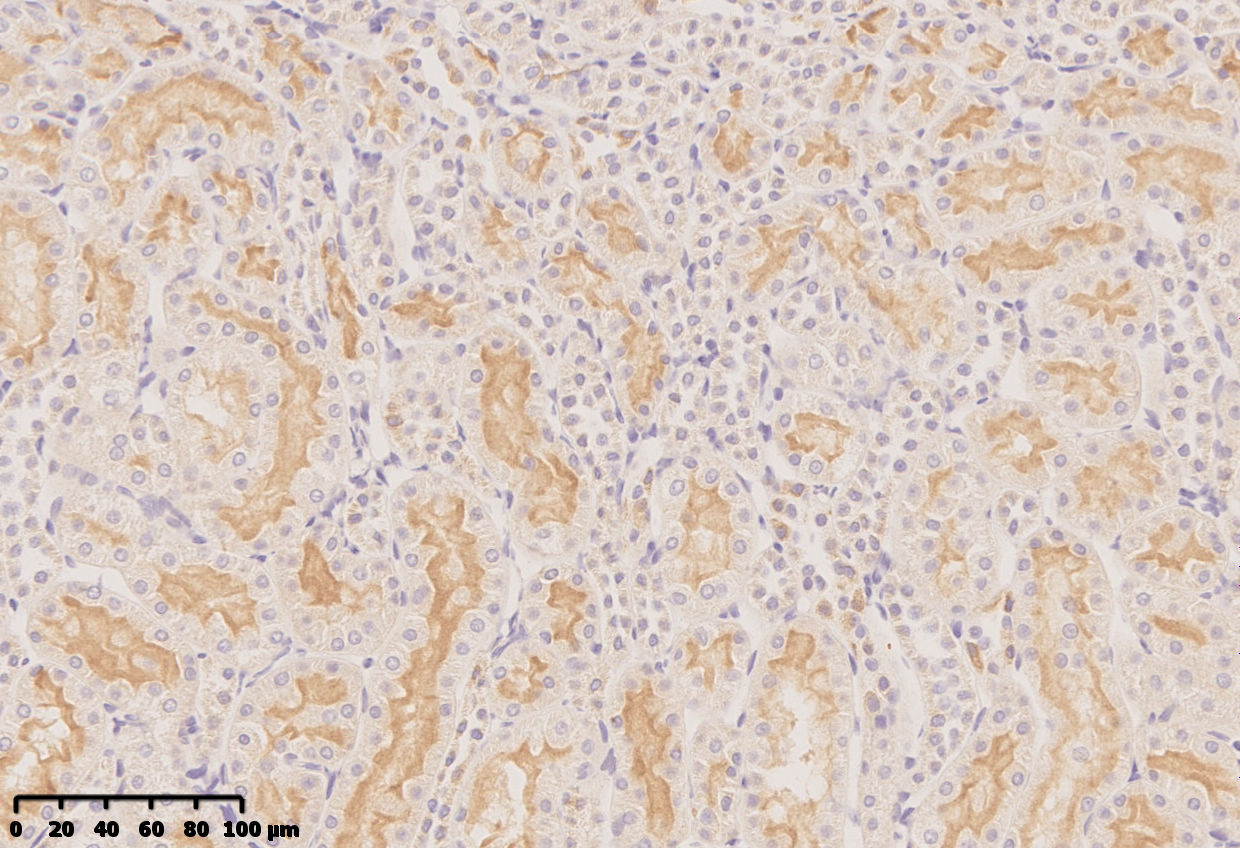

Supplement: Supplementary file 1 [file datasheet1.zip › ╣Γ├▄╢╚╔¿├Φ_final/GGT/ggt_HPL-3.jpg]

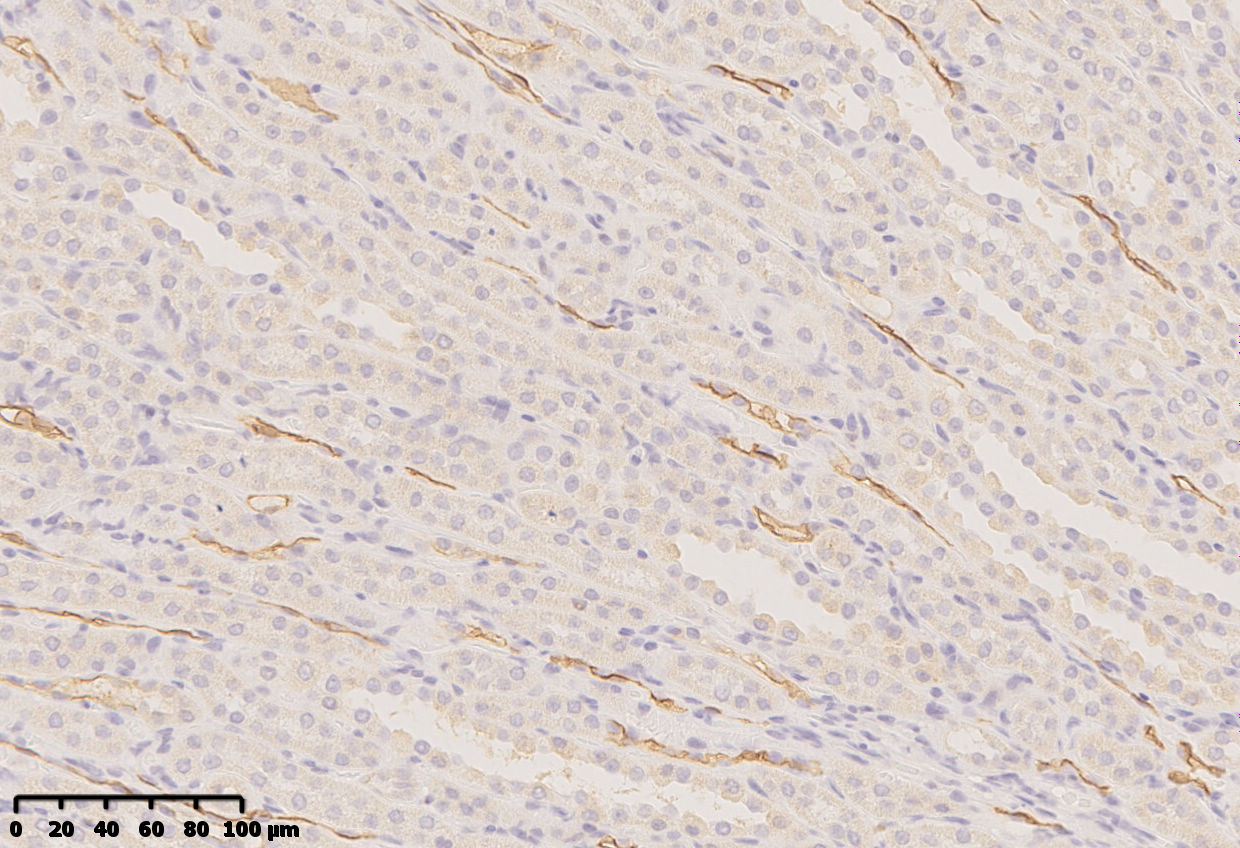

Supplement: Supplementary file 1 [file datasheet1.zip › ╣Γ├▄╢╚╔¿├Φ_final/GGT/ggt_con-1.jpg]

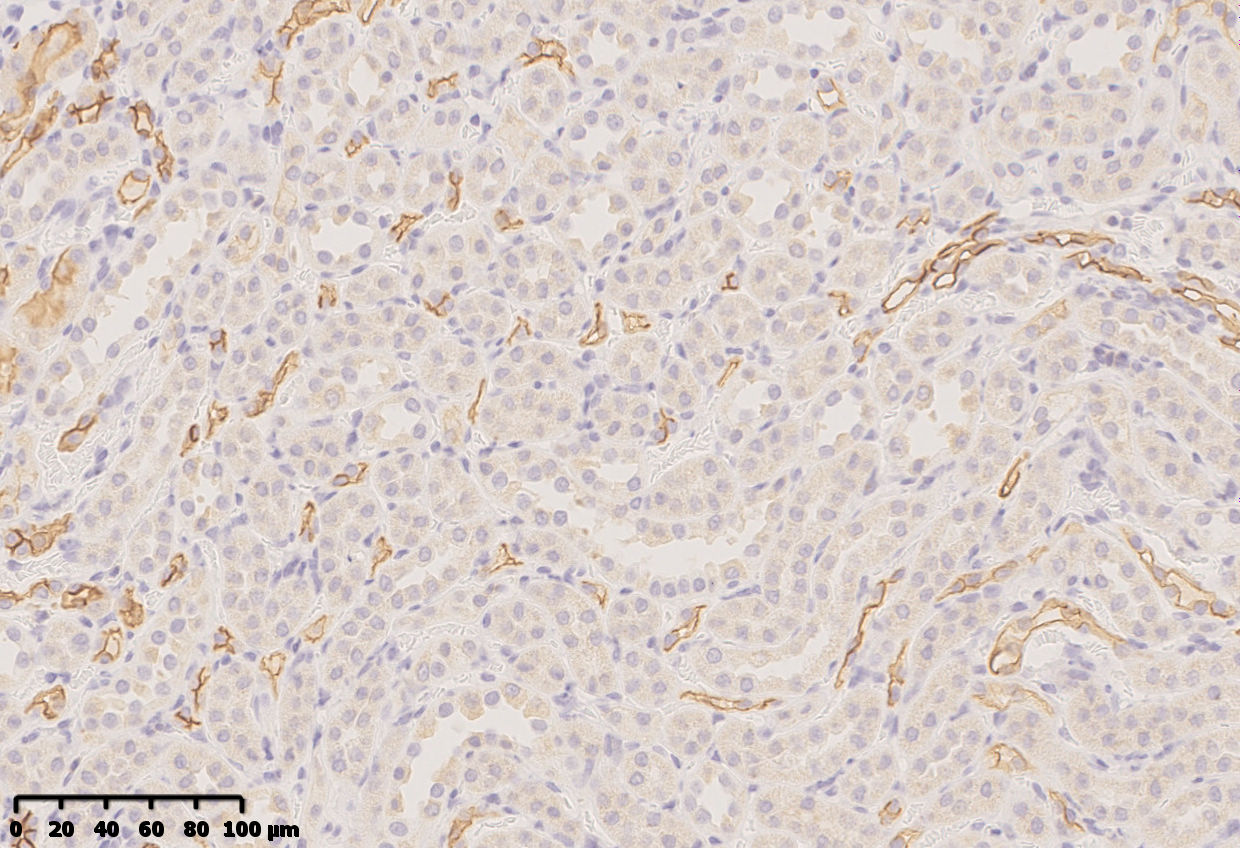

Supplement: Supplementary file 1 [file datasheet1.zip › ╣Γ├▄╢╚╔¿├Φ_final/GGT/ggt_con-2.jpg]

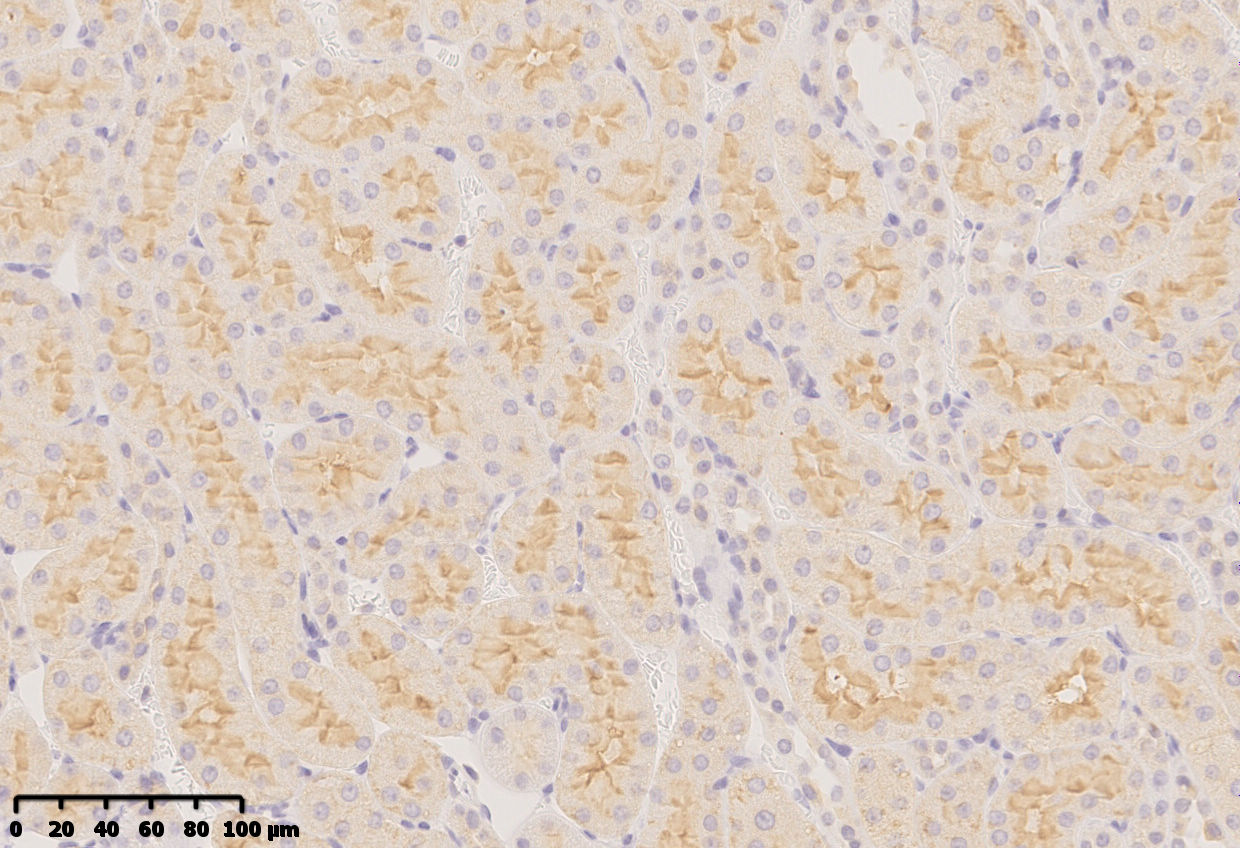

Supplement: Supplementary file 1 [file datasheet1.zip › ╣Γ├▄╢╚╔¿├Φ_final/GGT/ggt_con-3.jpg]

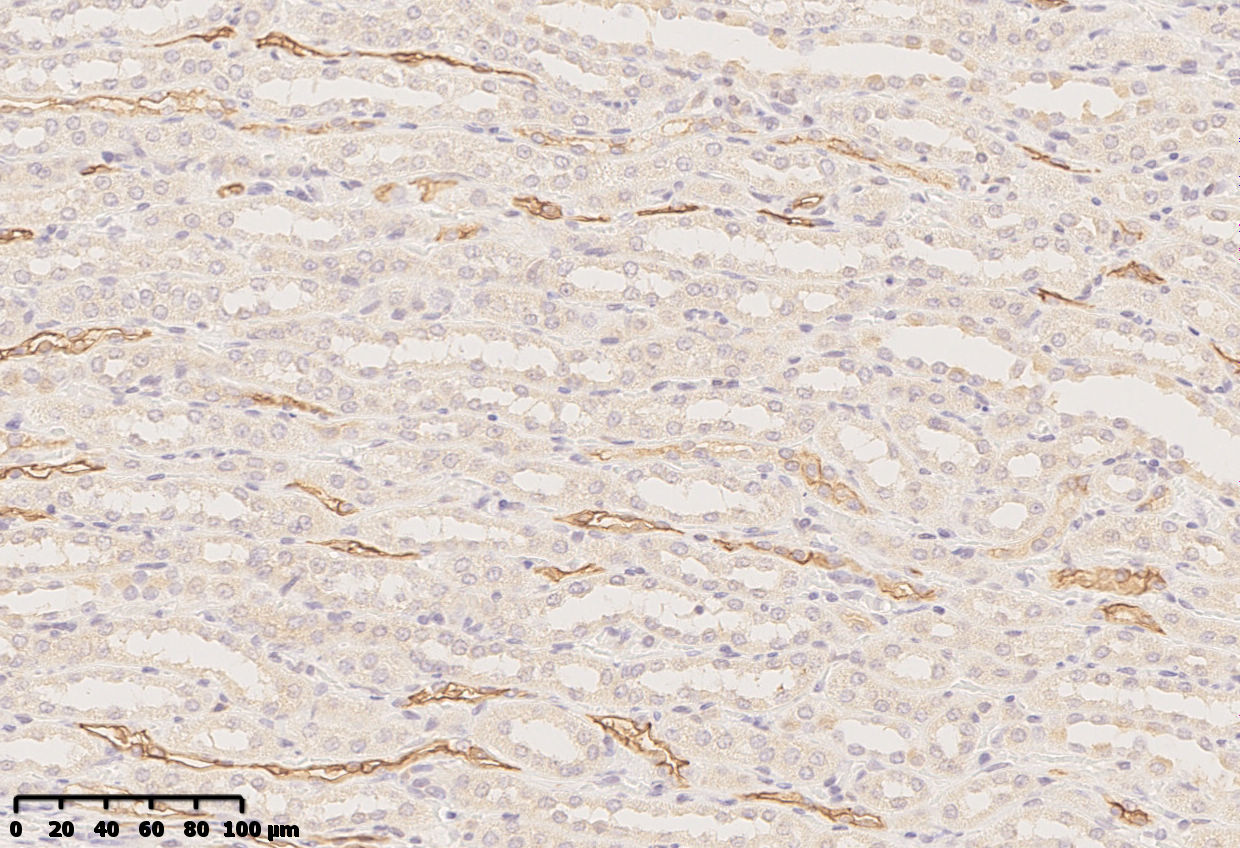

Supplement: Supplementary file 1 [file datasheet1.zip › ╣Γ├▄╢╚╔¿├Φ_final/GGT/ggt_ovx-1.jpg]

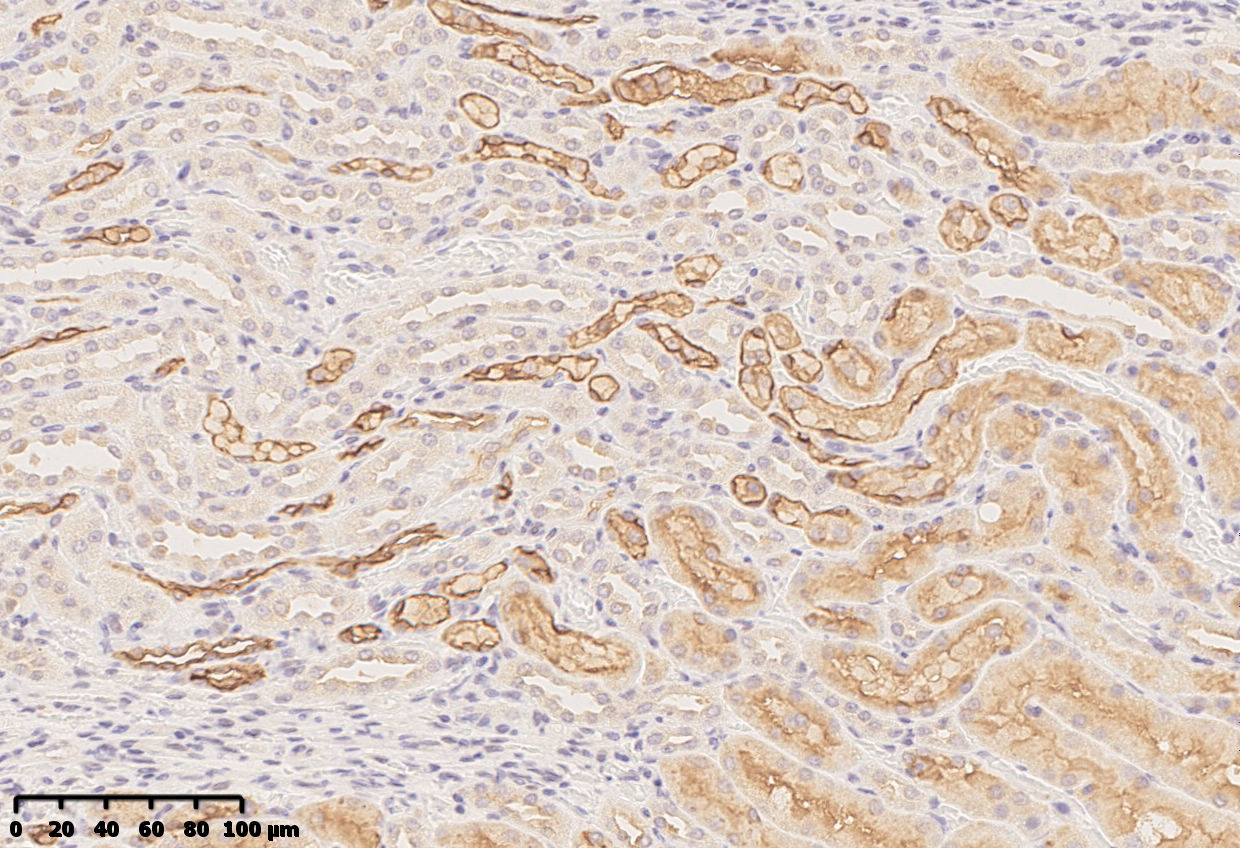

Supplement: Supplementary file 1 [file datasheet1.zip › ╣Γ├▄╢╚╔¿├Φ_final/GGT/ggt_ovx-2.jpg]

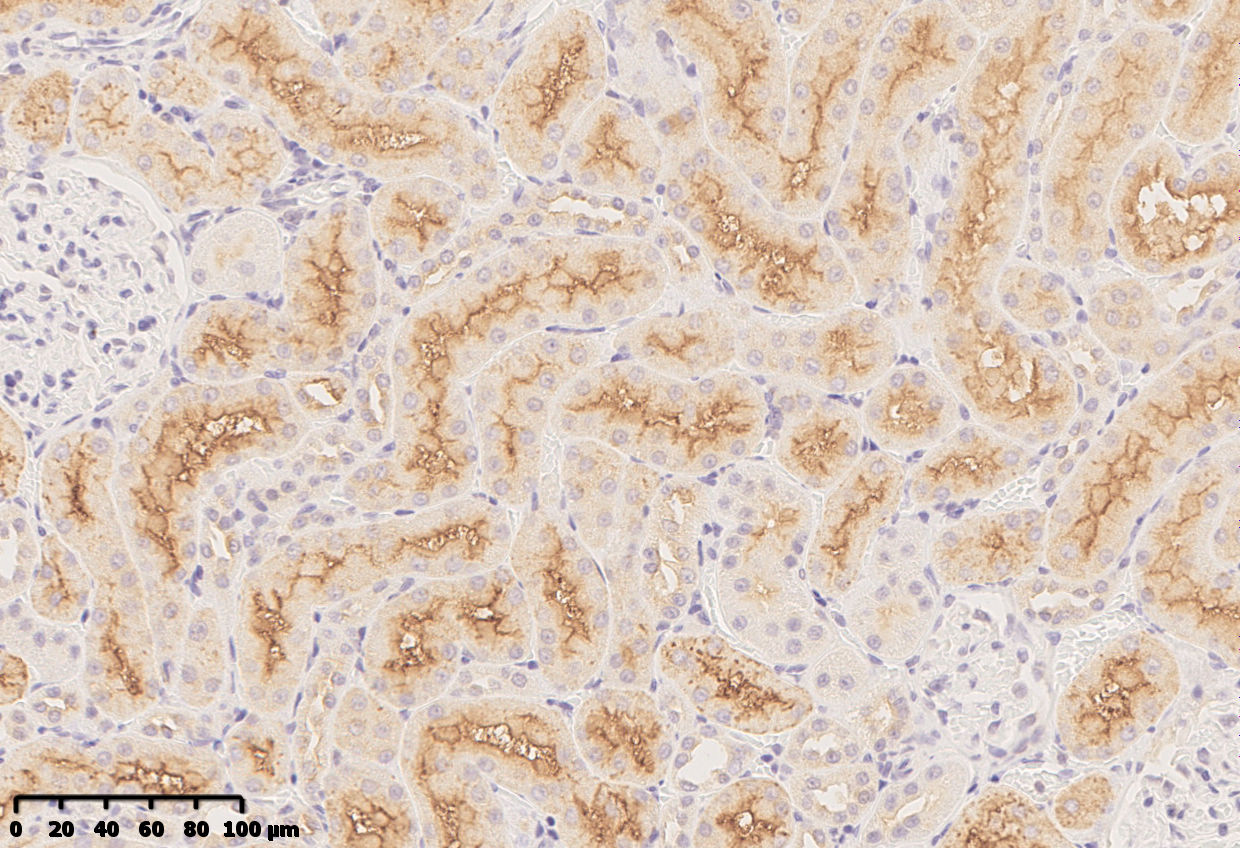

Supplement: Supplementary file 1 [file datasheet1.zip › ╣Γ├▄╢╚╔¿├Φ_final/GGT/ggt_ovx-3.jpg]

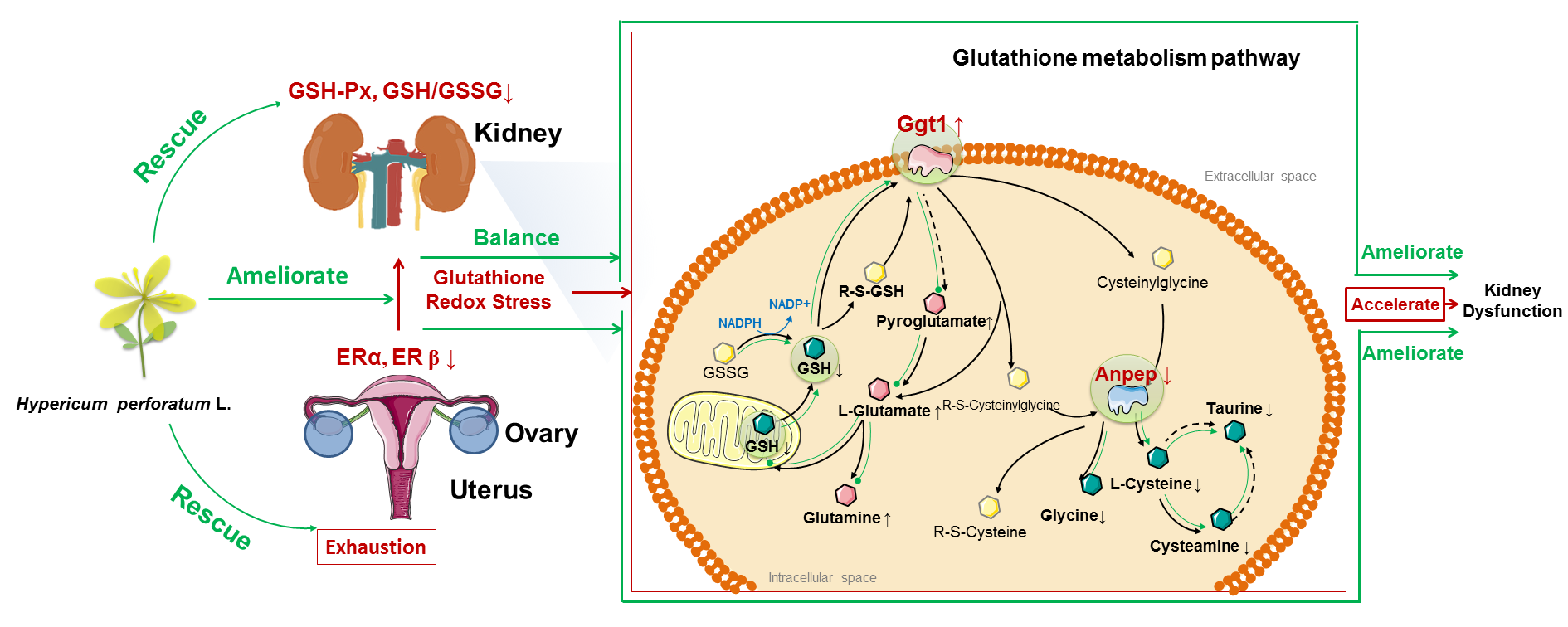

Supplement: Supplementary file 2 [file image1.tif]
