# Supplementary material for: Hypericum perforatum L. Regulates Glutathione Redox Stress and Normalizes Ggt1/Anpep Signaling to Alleviate OVX-Induced Kidney Dysfunction
Source: Front Pharmacol. 2021 Apr 26;12:628651. doi: 10.3389/fphar.2021.628651 (PMC8109178; doi:10.3389/fphar.2021.628651)
Supplement: Supplementary file 3 [file datasheet2.docx]

**Supplementary Information**

***Hypericum perforatum* L. regulates** **glutathione redox stress and normalizes Ggt1/Anpep signalling to alleviate OVX-induced kidney dysfunction**

*Yan-Ru Liu^a,+^, Ning-Juan Yang ^a,+^, Lin Chen ^a,+^, Zhi-Shu Tang ^a,*^, Jin-Ao Duan^b,*^, Jing Sun^a^, Zhong-Xing Song**^a^, Jin-Hang Hu^a^, Meng-Li Zhao^a^, Xin-Bo Shi**^a^*

*^a^ Shaanxi Province Key Laboratory of New Drugs and Chinese Medicine Foundation Research,* *Shaanxi Collaborative Innovation Center Medicinal Resources Industrialization,* *Shaanxi University of Chinese Medicine, 712083, Xianyang, P.R. China*

*^b^ Key Laboratory for High Technology Research of TCM Formulae and Jiangsu Collaborative Innovation Center of Chinese Medicinal Resources Industrialization，**Nanjing University of Chinese Medicine，210023，Nanjing, P.R. China*

*^*^Correspongding authors:*

*Zhi-Shu Tang: Shaanxi Collaborative Innovation Center of Chinese Medicinal Resource Industrialization, Shaanxi University of Chinese Medicine, 712083, Xianyang, P.R. China, Tel/Fax: +86 029 38185060, E-mail: tzs6565@163.com*

*Jin-Ao Duan: Key Laboratory for High Technology Research of TCM Formulae and Jiangsu Collaborative Innovation Center of Chinese Medicinal Resources Industrialization, Nanjing University of Chinese Medicine，210023, Nanjing, P.R. China, Tel/Fax: +86 029 38185060, E-mail:* *dja@njutcm.edu.cn.*

*^+^These authors contributed equally to this paper.*

**METHOD SECTION**

**1. Supplementary method I for *Hypericum perforatum* L.(HPL) flavonoids determination**

**LC separations conditions:**

UPLC-PDA analysis was performed on a Waters Acquity UPLC H-Class system (Waters Corporation, Milford, MA, USA). In brief, after a 2-μL injection, samples were separated on an Acquity H-class BEH-C18 column (, 50 × 2.1 mm, 1.7 μm) in 0.2% formic acid-water (A) and acetonitrile (B) gradient system at a column temperature of 25°C and a flow rate of 0.2 mL/min. The gradient method was 0~1 min，3% B；1~2 min，3%~15% B；2~4 min，15% B；4~10 min，15%~27% B；10~11 min，27% B；11~13 min，27%~35% B；13~14 min，35% B；14~15 min，35%~3% B. The chromatograms of four batches HPL extracts and mixed standards were acquired as follows:

**
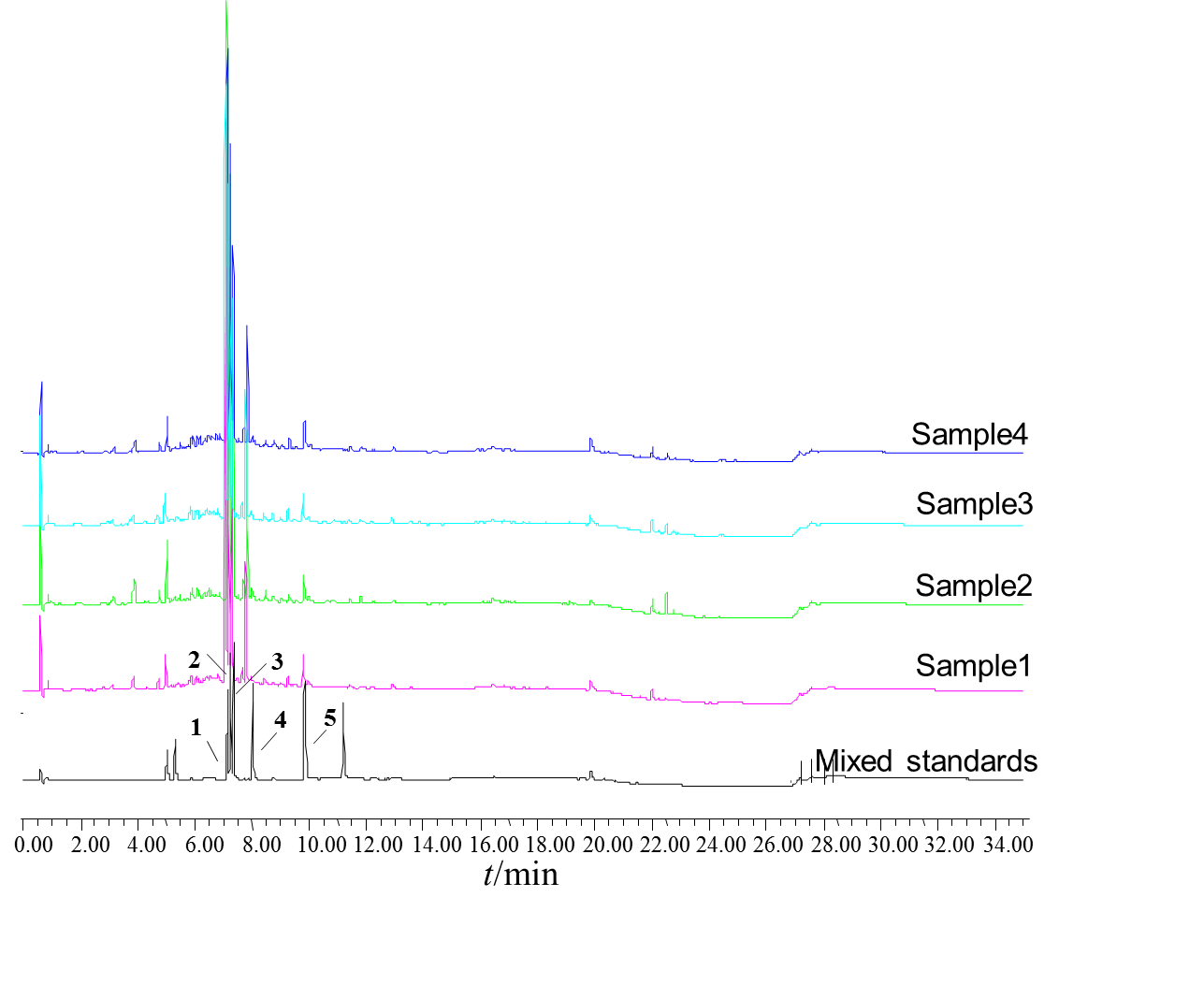
**

**Figure SI** Comparisons Fingerprint chromatograms of four batches HPL extracts and mixed standards

1. rutin（7.216 min）；2. hyperoside（7.346 min）；3. isoquercitin（7.448 min）；

4. quercitrin（8.122 min）；5. quercetin（10.027 min）

**Linearity**

The linearity was validated by a calibration curve calculated based on regression analysis of all commercially available standards for components of HPL extracts. The concentration ranges for the calibration curves for each analyte and the linear regression coefficient (r) were generated using the linearity equation of y= ax + b, where y represents the analyte peak area and x is the concentration of each calibration standard via linear regression analysis.

**Table SI** Linearity, regressions coefficents, limits of detection and quantification of HPL extracts flavonoids.

| Standards | Linear(μg·mL^-1^) | Regression equation | Regressions coefficents, *r* | LOD (μg·mL^-1^) | LOQ (μg·mL^-1^) |
| --- | --- | --- | --- | --- | --- |
| Rutin | 3.83～122.50 | *Y*=7 020*X*-1 541 | 0.999 8 | 0.050 | 0.250 |
| Hyperoside | 4.45～142.50 | *Y*=9 070*X*-2 486 | 0.999 5 | 0.057 | 0.133 |
| Isoquercitin | 5.90～151.25 | *Y*=9 080*X*-3 614 | 0.999 2 | 0.067 | 0.200 |
| Quercitrin | 1.05～21.25 | *Y*=61 200*X*-4 747 | 0.999 1 | 0.020 | 0.667 |
| Quercetin | 1.07～22.29 | *Y*=13 *100X*-10 183 | 0.999 8 | 0.107 | 0.167 |

**Stability, precision, and recovery**

Stability test was performed with 48h. The precision and recovery were assessed using the 5 standard analytes at 100% concentration levels. The RSD percentages of the chromatographic method were calculated at three concentration levels and should not exceed 2%. The precision RSD% of standards solution were between 0.31% and 0.91%, and the stability RSD% was between 0.24% and 1.76%.

Recovery was assessed by adding standard solutions at three concentration levels (80%, 100% and 120%) to real samples before performing the sample preparation procedure described above and to three additional blank samples. The recovery rate (R) was determined as R%=concentration before/concentration after×100%. The mean analytical recovery ranged from 96.49% to 101.45% for all standards.

**Table SII** Recovery tests of HPL extracts flavonoids.

|  | Rutin/% | Hyperoside /% | Isoquercitin /% | Quercitrin /% | Quercetin /% |
| --- | --- | --- | --- | --- | --- |
| 80% | 95.56 | 102.2 | 99.00 | 101.10 | 98.80 |
| 100% | 96.75 | 102.3 | 98.12 | 99.12 | 97.19 |
| 120% | 97.16 | 99.87 | 98.74 | 98.80 | 98.18 |
| mean | 96.49 | 101.45 | 98.62 | 99.67 | 98.05 |
| RSD/% | 0.13 | 1.3 | 0.45 | 1.24 | 1.24 |

S

**Sample determination**

The extracts were treated as described in CHP 2020, chromatogram analyses were conducted, and the main components were determined. The method was applied to the analysis of 5 main compounds (n=3, mean%±SEM): Rutin (%):1.09%±0.010, Hyperoside (%):0.65%±0.007, Isoquercitin (%):0.45%±0.004, Quercitrin (%):0.04%±0.001 , Quercetin (%):0.07% ±0.002.

**2. Table SIII** Targeted proteins MRM for Q1-Q3, DP, CE Optimization

| Protein | Peptide | Q1 | Q3 | DP | CE | ion type | lable |
| --- | --- | --- | --- | --- | --- | --- | --- |
| Gamma-glutamyltranspeptidase 1 (GGT1) | VLQEGETVTMPK | 666.347402 | 1119.53505 | 79.7 | 32.8 | +2y10 | light |
|  |  | 666.347402 | 991.476473 | 79.7 | 32.8 | +2y9 | light |
|  |  | 666.347402 | 862.43388 | 79.7 | 32.8 | +2y8 | light |
|  |  | 666.347402 | 676.369823 | 79.7 | 32.8 | +2y6 | light |
|  |  | 666.347402 | 476.253731 | 79.7 | 32.8 | +2y4 | light |
|  |  | 666.347402 | 341.218332 | 79.7 | 32.8 | +2b3 | light |
|  |  | 670.354502 | 1127.54925 | 79.7 | 32.8 | +2y10 | heavy |
|  |  | 670.354502 | 999.490672 | 79.7 | 32.8 | +2y9 | heavy |
|  |  | 670.354502 | 870.448079 | 79.7 | 32.8 | +2y8 | heavy |
|  |  | 670.354502 | 684.384022 | 79.7 | 32.8 | +2y6 | heavy |
|  |  | 670.354502 | 484.26793 | 79.7 | 32.8 | +2y4 | heavy |
|  |  | 670.354502 | 341.218332 | 79.7 | 32.8 | +2b3 | heavy |
| Alanyl (Membrane) aminopeptidase (ANPEP, CD13) | ALGDTPAPNIDTTELVER | 637.993455 | 962.478916 | 77.6 | 32.3 | +3y8 | light |
|  |  | 637.993455 | 847.451973 | 77.6 | 32.3 | +3y7 | light |
|  |  | 637.993455 | 645.356616 | 77.6 | 32.3 | +3y5 | light |
|  |  | 637.993455 | 516.314023 | 77.6 | 32.3 | +3y4 | light |
|  |  | 637.993455 | 403.229959 | 77.6 | 32.3 | +3y3 | light |
|  |  | 641.329544 | 972.487185 | 77.6 | 32.3 | +3y8 | heavy |
|  |  | 641.329544 | 857.460242 | 77.6 | 32.3 | +3y7 | heavy |
|  |  | 641.329544 | 655.364885 | 77.6 | 32.3 | +3y5 | heavy |
|  |  | 641.329544 | 526.322292 | 77.6 | 32.3 | +3y4 | heavy |
|  |  | 641.329544 | 413.238228 | 77.6 | 32.3 | +3y3 | heavy |

*Q1 is the peptide precursor ion, and Q3 is the peptide product ion, DP is the declustering potential, and CE is the collision energy.

**Light: target peptide; Heavy: standards.

**3. Supplementary method II for LC-MS metabolite determination**

**LC conditions**

The LC/MS/MS system consisted of a Agilent 1260 LC instrument connected to a AB sciex 4500 triple quadrupole mass spectrometer via ESI interface. The mobile phase consisted of acetonitrile (A) and water containing 0.2% formic acid (B). An A Agilent 5 TC-C_18_ (2)-C_18_ column (250 mm×4.6 mm，5µm) was used. Gradient elution program was used: 0~25 min，0.2% ~99.8%B；25～30 min，100% B；30～31 min，99.8%～0.2% B；31～35 min，0.2% B. The flow rate was 600 µL/min. The HPLC effluent was introduced into the mass spectrometer without splitting. The column temperature was set at 30 ◦C, inject volume 5μL.

**MRM conditions**

All quantified MS data were acquired on an AB Sciex Qtrap 4500 hydrid, triple quadrupole, linear ion trap mass spectrometer equipped with an electrospray ionization (ESI) source and Analyst 1.6.2 software (AB SCIEX, Foster City, CA, USA). The optimized MRM information of declustering Potential (DP), collision energy (CE), and cell exit potential (CXP) for metabolite standards was presented in Table S13. The The optimized MS parameters were curtain gas (CUR) 35 psi, temperature (TEM) 450 °C, 40 psi of both gas source 1 and gas source 2, ion spray voltage (IS), 4.5 kV for negative ion (NI) modes, 5.0kV for positive ion (PI) modes. The TIC MRM extracted ion chromatogram is shown in Figure SII.

**Table SIV** LC/MS–MS data for 43 amino acids, small molecular acids and bases.

| No. | Metabolites | Retention time （min） | Precursor ion（Da） | Product ion （Da） | DP | CE | CXP |
| --- | --- | --- | --- | --- | --- | --- | --- |
| 1 | Citrate | 5.24 | 191.0 | 111.1 | -48 | -17 | -7 |
| 2 | Creatine | 1.88 | 132.0 | 90.0 | -38 | -14 | -6 |
| 3 | Creatinine | 1.46 | 112.1 | 41.0 | -23 | -32 | -10 |
| 4 | Hippurate | 9.46 | 178.0 | 77.1 | -60 | -21 | -7 |
| 5 | Taurine | 1.91 | 124.1 | 80.0 | -48 | -28 | -7 |
| 6 | 2-Oxoglutarate | 6.73 | 145.0 | 101.0 | -30 | -13 | -7 |
| 7 | 3- Indole acetate | 12.16 | 174.0 | 130.0 | -50 | -15 | -9 |
| 8 | Oxaloacetate | 7.06 | 131.0 | 87.0 | -46 | -13 | -12 |
| 9 | Pyruvate | 5.55 | 87.0 | 30.0 | -33 | -10 | -12 |
| 10 | Lactate | 4.10 | 89.0 | 43.0 | -56 | -14 | -11 |
| 11 | 3-Hydroxybutyrate | 1.19 | 103.0 | 59.0 | -118 | -13 | -9 |
| 12 | Urate | 4.92 | 167.0 | 124.0 | -55 | -22 | -11 |
| 13 | Allantoin | 2.19 | 157.0 | 97.0 | -43 | -20 | -6 |
| 14 | Guanidoaceticacid | 1.67 | 116.1 | 74.0 | -41 | -12 | -6 |
| 15 | GABA | 1.41 | 102.0 | 84.0 | -53 | -13 | -7 |
| 16 | L-Glutamate | 4.85 | 146.1 | 102.1 | -43 | -17 | -5 |
| 17 | Cytosine | 4.26 | 110.0 | 67.0 | -50 | -16 | -10 |
| 18 | Xanthine | 9.95 | 151.0 | 108.0 | -83 | -21 | -7 |
| 19 | Phenylalanine | 11.43 | 166.0 | 120.0 | 84 | 19 | 9 |
| 20 | L-Serine | 4.69 | 106.0 | 60.0 | 49 | 13 | 10 |
| 21 | β-Alanine | 4.24 | 90.0 | 30.0 | 50 | 11 | 8 |
| 22 | L-Histidine | 3.98 | 156.0 | 110.0 | 30 | 18 | 8 |
| 23 | Glutamine | 4.74 | 147.0 | 84.0 | 49 | 22 | 8 |
| 24 | L-Leucine | 9.82 | 132.0 | 86.0 | 49 | 16 | 13 |
| 25 | L-Arginine | 4.01 | 175.1 | 70.0 | 43 | 22 | 10 |
| 26 | L-Tryptophan | 12.75 | 205.1 | 188.1 | 63 | 14 | 6 |
| 27 | L-Threonine | 4.80 | 120.1 | 74.1 | 45 | 15 | 7 |
| 28 | Glycine | 4.62 | 76.0 | 30.1 | 37 | 21 | 8 |
| 29 | L-Alanine | 4.71 | 90.1 | 44.0 | 43 | 14 | 12 |
| 30 | L-Proline | 5.13 | 116.1 | 70.0 | 50 | 20 | 11 |
| 31 | Phenylacetylglycine | 6.11 | 194.1 | 91.1 | 53 | 28 | 6 |
| 32 | Aspartate | 4.84 | 134.0 | 74.0 | 61 | 19 | 12 |
| 33 | L-Cysteine | 4.61 | 122.0 | 76.0 | 41 | 18 | 12 |
| 34 | N,N- Dimethylglycine | 4.98 | 104.1 | 58.1 | 57 | 18 | 5 |
| 35 | Pyroglutamate | 8.24 | 130.1 | 84.0 | 151 | 17 | 6 |
| 36 | 1-Methylhistidine | 4.02 | 170.1 | 124.1 | 53 | 19 | 9 |
| 37 | Carnitine | 4.34 | 162.1 | 103.1 | 65 | 22 | 7 |
| 38 | TMAO | 4.29 | 76.1 | 59.0 | 85 | 16 | 6 |
| 39 | Urea | 5.24 | 61.0 | 44.0 | 57 | 23 | 13 |
| 40 | Choline | 4.26 | 104.0 | 60.0 | 57 | 23 | 13 |
| 41 | Spermine | 3.10 | 203.2 | 129.1 | 50 | 17 | 4 |
| 42 | Betaine | 5.09 | 118.0 | 59.0 | 69 | 23 | 9 |
| 43 | Araitol | 5.07 | 151.1 | 89.0 | -58 | -16 | -9 |

**Table SV** Linearity, regressions coefficents, limits of quantification (LOQ) of the 43 standards.

| No. | Metabolites | Linear equation  (correlation coefficient, r) | Linear range  (µg·mL^-1^) | LOQ (ng·mL^-1^) |
| --- | --- | --- | --- | --- |
| 1 | Citrate | y = 1.29×10^6^ x -3.77×10^5^  (r = 0.9999) | 3.47-17.35 | 9.91 |
| 2 | Creatine | y = 30.41 x + 3.67×10^5^  (r = 0.9935)  y = 3.42×10^5^ x + 2594.92  (r = 0.9968) | 0.005-51.98 | 2.63 |
| 3 | Creatinine | y = 3.29×10^6^x + 3698.80  (r = 0.9995) | 0.002-0.02 | 0.20 |
| 4 | Hippurate | y = 4.80×10^5^x + 1548.61  (r = 0.9989) | 0.001-0.09 | 0.48 |
| 5 | Taurine | y = 8.58×10^5^x + 5479.70  (r = 0.9996) | 0.002-1.04 | 0.11 |
| 6 | 2-Oxoglutarate | y = 55185.02x + 2792.41  (r = 0.9977)  y = 42965.20x + 1.49×10^6^  (r = 0.9977) | 0.03-67.70 | 338.5 |
| 7 | 3- Indole acetate | y = 1.63×10^6^x + 3446.37  (r = 0.9971) | 7.27-69.97 | 0.70 |
| 8 | Oxaloacetate | y = 19683.55x + 6.77×10^4^  (r = 0.9900) | 2.46-12.30 | 1230.00 |
| 9 | Pyruvate | y = 19831.42x +1.11×10^5^  (r = 0.9969) | 12.61-216.80 | 5475.00 |
| 10 | Lactate | y = 14841.00x +10078  (r = 0.9997) | 0.05-4.52 | 62.00 |
| 11 | 3-Hydroxybutyrate | y =4105.73x +8.10×10^5^  (r = 0.9919) | 19.45-389.00 | 2431.25 |
| 12 | Urate | y =6169.90x +669.23  (r = 0.9987)  y =9616.87x +9822.08  (r = 0.9981) | 0.07-29.6 | 7.40 |
| 13 | Allantoin | y =1.23×10^5^x +4405.00  (r = 0.9940) | 0.01-0.86 | 1.71 |
| 14 | Guanidoaceticacid | y =1.11×10^5^x +198.29  (r = 0.9987) | 0.03-0.61 | 30.5 |
| 15 | GABA | y =1.38×10^6^x +1535.46  (r = 0.9933) | 2.09-20.90 | 1045.00 |
| 16 | L-Glutamate | y = 1.38×10^5^x +1.70×10^6^  (r = 0.9903)  y = 8.88×10^5^x +5.03×10^4^  (r = 0.9966) | 0.03-62.34 | 62.00 |
| 17 | Cytosine | y = 1.00×10^6^x +1.21×10^5^  (r = 0.9987) | 0.01-1.18 | 1.19 |
| 18 | Xanthine | y =3036.07x +324.11  (r = 0.9997) | 1.66-83.00 | 1076.73 |
| 19 | Phenylalanine | y = 1.15×10^5^x +1.70×10^6^  (r = 0.9903)  y = 4.13×10^5^x +9.79×10^6^  (r = 0.9908) | 0.07-74.50 | 2.26 |
| 20 | L-Serine | y = 4.13×10^5^x +5.92×10^4^  (r = 0.9984)  y = 3.34×10^5^x +2.54×10^5^  (r = 0.9985) | 0.08-16.80 | 0.84 |
| 21 | β-Alanine | y = 1.69×10^4^x +2398.04  (r = 0.9918) | 0.52-2.06 | 179.52 |
| 22 | L-Histidine | y = 2.70×10^6^x +1.03×10^5^  (r = 0.9961) | 0.01-1.18 | 1.18 |
| 23 | Glutamine | y = 9.48×10^5^x +5878.63  (r = 0.9978)  y =6.17×10^5^x +9.07×10^5^  (r = 0.9989) | 4.85-48.3 | 2.42 |
| 24 | L-Leucine | y = 5.47×10^6^x -14795.29  (r = 0.9970)  y =-7.08×10^7^x +4.49×10^6^  (r = 0.9973) | 0.01-6.02 | 11.90 |
| 25 | L-Arginine | y =-6.38×10^5^x +1.79×10^6^  (r = 0.9961)  y =-9.68×10^5^x +6.18×10^4^  (r = 0.9985) | 0.02-76.00 | 1.52 |
| 26 | L-Tryptophan | y =-2.63×10^6^x +8.20×10^5^  (r = 0.9968)  y =3.78×10^6^x +6534.17  (r = 0.9991) | 0.001-14.50 | 0.15 |
| 27 | L-Threonine | y =83392.80x +19734.58  (r = 0.9952) | 0.05-0.54 | 27.00 |
| 28 | Glycine | y = 6062.3x + 23183  (r = 0.9974) | 2.47-12.35 | 3.31 |
| 29 | L-Alanine | y =1.45×10^6^x +3.79×10^4^  (r = 0.9907) | 0.02-5.10 | 8.50 |
| 30 | L-Proline | y =2.45×10^6^x +3.96×10^4^  (r = 0.9938)  y =4.06×10^6^x +7.56×10^4^  (r = 0.9990) | 0.07-26.80 | 3.48 |
| 31 | Phenylacetylglycine | y =1.73×10^7^x +11296.62  (r = 0.9995) | 0.0001-0.008 | 0.04 |
| 32 | Aspartate | y =1.05×10^5^x +2.75×10^5^  (r = 0.9980)  y =4.48×10^5^x +5296.68  (r = 0.9998) | 0.03-26.5 | 2.65 |
| 33 | L-Cysteine | y =509.36x +1636.75  (r = 0.9926) | 2.58-6.45 | 129.00 |
| 34 | N,N- Dimethylglycine | y =1.51×10^6^x +17563.15  (r = 0.9999) | 0.02-1.53 | 7.65 |
| 35 | Pyroglutamate | y =1.32×10^5^x +6.09×10^4^  (r = 0.9901) | 0.76-1.53 | 218.57 |
| 36 | 1-Methylhistidine | y =2.69×10^6^x +22129.28  (r = 0.9997) | 0.005-0.48 | 1.62 |
| 37 | Carnitine | y =4.18×10^6^x +1.18×10^5^  (r = 0.9997) | 0.004-4.19 | 0.21 |
| 38 | TMAO | y =3.60×10^6^x +7066.44  (r = 0.9999) | 0.002-0.16 | 1.57 |
| 39 | Urea | y =45770.28x +6.90×10^4^  (r = 0.9990) | 0.17-1.68 | 1.67 |
| 40 | Choline | y =75778.92x +1.26×10^6^  (r = 0.99903) | 0.34-68.3 | 9.76 |
| 41 | Spermine | y =1.13×10^6^x -1.15×10^5^  (r = 0.9998) | 0.18-3.55 | 11.83 |
| 42 | Betaine | y =1.09×10^6^x+3.24×10^5^  (r = 0.9998)  y =1.54×10^6^x+2.07×10^5^  (r = 0.9997) | 0.11-11.00 | 36.23 |
| 43 | Araitol | y = 39651x + 3295.4  (r = 0.9999) | 2.23-11.10 | 79.75 |

**
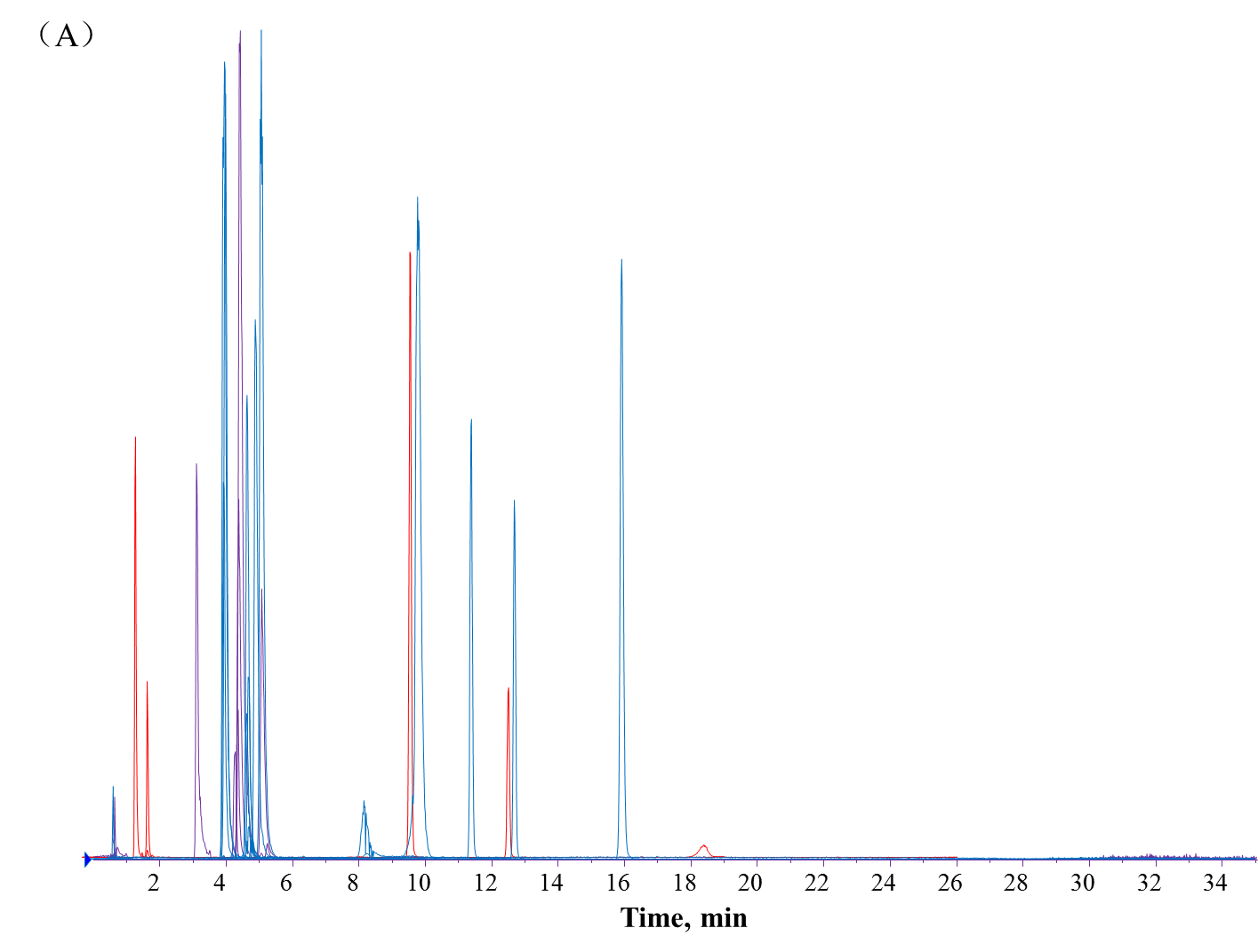
**

**
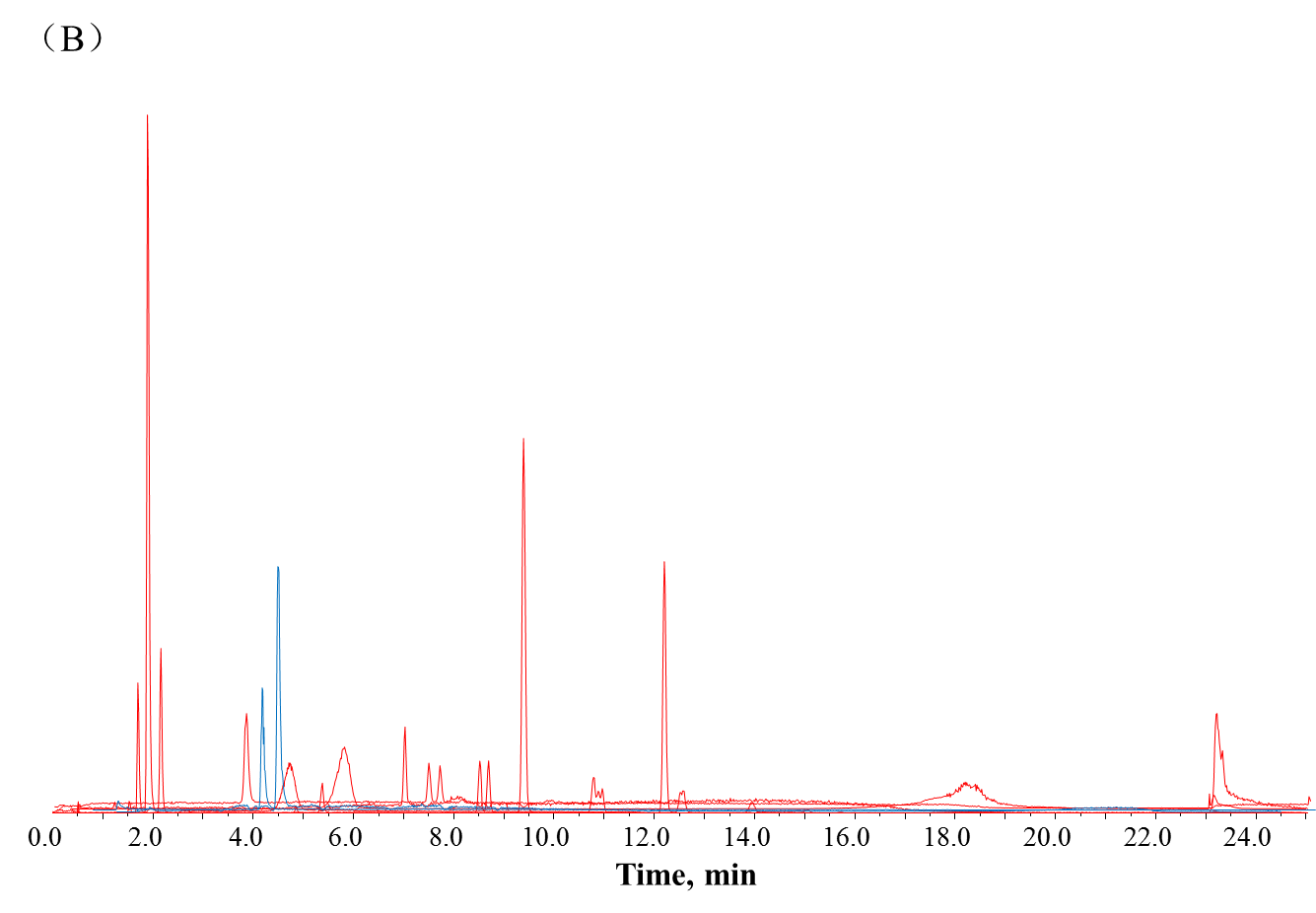
**

**Figure SII** Extracted ion chromatograms of a mixture of 55 standards under positive ion (A) and negative ion (B) mode.

* MRM chromatograms in blue presents amino acids；MRM chromatograms in red presents organic acids and base metabolites.

**RESULTS SECTION**

**Table S1** TOP 22 proteins from Gene Ontology (GO) category analysis.

| **No** | **ProteinName** | **Gene**  **Name** | **Log2FC**  **(OVX/CON)** | **Control** | **OVX** | **Hyperoside** |
| --- | --- | --- | --- | --- | --- | --- |
| 1 | Alpha-2-HS-glycoprotein | Ahsg | 0.551 | 0.83±0.07 | 1.22±0.08 | 0.97±0.02 |
| 2 | Protein AMBP | Ambp | 1.034 | 0.75±0.10 | 1.54±0.20 | 1.03±0.01 |
| 3 | Alanyl (Membrane) aminopeptidase | Anpep | -0.222 | 1.00±0.00 | 0.86±0.02 | 1.23±0.29 |
| 4 | Beta-2-microglobulin | B2m | 0.427 | 0.82±0.08 | 1.11±0.05 | 0.95±0.01 |
| 5 | Complement C3 | C3 | -0.833 | 1.21±0.12 | 0.68±0.12 | 0.89±0.00 |
| 6 | CRAMP (Fragment) | Camp | -0.956 | 1.27±0.08 | 0.66±0.19 | 1.44±0.03 |
| 7 | Cadherin-1 | Cdh1 | 0.589 | 0.87±0.09 | 1.31±0.10 | 1.05±0.00 |
| 8 | Collagen alpha-1(I) chain | Col1a1 | -0.724 | 1.14±0.07 | 0.69±0.12 | 0.91±0.01 |
| 9 | Lysosomal alpha-glucosidase | Gaa | -0.632 | 1.24±0.11 | 0.80±0.06 | 1.00±0.02 |
| 10 | Gamma-glutamyl hydrolase | Ggh | -0.382 | 0.96±0.01 | 0.74±0.02 | 0.80±0.01 |
| 11 | Gamma-glutamyltranspeptidase 1 | Ggt1 | 0.787 | 1.76±0.07 | 3.04±0.11 | 2.04±0.11 |
| 12 | Ig gamma-2B chain C region | Igh-1a | 1.131 | 0.88±0.03 | 1.93±0.47 | 0.67±0.01 |
| 13 | Lactoperoxidase (Predicted) | Lpo | -0.827 | 1.35±0.17 | 0.76±0.08 | 1.21±0.01 |
| 14 | Myosin-6 | Myh6 | -0.524 | 1.15±0.12 | 0.80±0.12 | 1.54±0.00 |
| 15 | Plasminogen activator, urokinase | Plau | 0.265 | 0.98±0.00 | 1.18±0.05 | 1.03±0.02 |
| 16 | Plasminogen | Plg | 0.425 | 0.95±0.08 | 1.28±0.13 | 0.92±0.01 |
| 17 | Prostaglandin-H2 D-isomerase | Ptgds | 0.484 | 0.93±0.01 | 1.29±0.11 | 1.11±0.01 |
| 18 | Retinoid-inducible serine carboxypeptidase | Scpep1 | 0.502 | 0.94±0.02 | 1.33±0.16 | 1.10±0.00 |
| 19 | Corticosteroid-binding globulin | Serpina6 | 0.591 | 0.93±0.04 | 1.40±0.10 | 0.84±0.01 |
| 20 | Neutral and basic amino acid transport protein rBAT | Slc3a1 | -0.853 | 1.32±0.16 | 0.73±0.14 | 1.25±0.01 |
| 21 | Trefoil factor 1 | Tff1 | 0.564 | 0.98±0.01 | 1.45±0.07 | 1.18±0.02 |

**Table S2** Enrichment list of differentially expressed proteins on cellular component, cellular function, and biochemical process. (cut-off valure 1.5-fold change, p value ˂ 0.05)

| **No.** | **Gene name** | **Abbreviation** | **Cellular Component** | **Molecular Function** | **Biochemical Process**  **(GO ID)** |
| --- | --- | --- | --- | --- | --- |
| 1 | Alpha-2-HS-glycoprotein | Ahsg | extracellular space(GO:0005615),  blood microparticle(GO:0072562),  extracellular matrix(GO:0031012),  protein complex(GO:0043234), | cysteine-type endopeptidase inhibitor activity (GO:0004869),  endopeptidase inhibitor activity (GO:0004866),  receptor signaling protein tyrosine kinase inhibitor activity (GO:0030294),  kinase inhibitor activity (GO:0019210) | negative regulation of endopeptidase activity(GO:0010951),  acute-phase response(GO:0006953),  cellular response to insulin stimulus(GO:0032869),  negative regulation of cell growth(GO:0030308),  negative regulation of protein tyrosine kinase activity(GO:0061099),  cerebral cortex development(GO:0021987),  negative regulation of phosphorylation(GO:0042326),  regulation of inflammatory response(GO:0050727),  protein complex assembly(GO:0006461),  negative regulation of insulin receptor signaling pathway(GO:0046627) |
| 2 | Protein AMBP | Ambp | extracellular space(GO:0005615),  extracellular exosome(GO:0070062),  blood microparticle(GO:0072562),  plasma membrane(GO:0005886),  cell surface (GO:0009986),  intracellular membrane-bounded organelle(GO:0043231), | small molecule binding (GO:0036094), serine-type endopeptidase inhibitor activity (GO:0004867),  IgA binding (GO:0019862),  heme binding (GO:0020037),  protein homodimerization activity (GO:0042803) | negative regulation of endopeptidase activity(GO:0010951),  protein catabolic process(GO:0030163),  protein-chromophore linkage(GO:0018298) |
| 3 | Alanyl (Membrane) aminopeptidase | Anpep | cytoplasm (GO:0005737), plasma membrane  (GO:0005886), | zinc ion binding (GO:0008270),  aminopeptidase activity (GO:0004177),  peptide binding (GO:0042277),  metalloaminopeptidase activity (GO:0070006) | proteolysis (GO:0006508),  cellular aromatic compound metabolic process (GO:0006725),  peptide catabolic process (GO:0043171),  negative regulation of renal sodium excretion  (GO:0035814), |
| 4 | Beta-2-microglobulin | B2m | extracellular space(GO:0005615),  extracellular exosome(GO:0070062),  external side of plasma membrane  (GO:0009897),  focal adhesion(GO:0005925),  Golgi apparatus(GO:0005794),  MHC class I protein complex  (GO:0042612) |  | protein refolding(GO:0042026),  cellular response to lipopolysaccharide(GO:0071222),  positive regulation of T cell cytokine production(GO:0002726),  negative regulation of receptor binding(GO:1900121),  regulation of membrane depolarization(GO:0003254),  antigen processing and presentation of exogenous protein antigen via MHC class Ib, TAP-dependent(GO:0002481),  positive regulation of T cell mediated cytotoxicity(GO:0001916),  positive regulation of receptor-mediated endocytosis(GO:0048260),  T cell differentiation in thymus(GO:0033077) |
| 5 | Complement C3 | C3 | extracellular space(GO:0005615), | endopeptidase inhibitor activity (GO:0004866),  C5L2 anaphylatoxin chemotactic receptor binding (GO:0031715),  lipid binding (GO:0008289),  cofactor binding (GO:0048037) | complement activation, alternative pathway(GO:0006957),  blood coagulation(GO:0007596),  complement activation, classical pathway(GO:0006958),  inflammatory response(GO:0006954),  complement activation(GO:0006956),  tolerance induction(GO:0002507),  positive regulation of ERK1 and ERK2 cascade (GO:0070374),  regulation of triglyceride biosynthetic process (GO:0010866),  response to estradiol l(GO:0032355),  positive regulation of developmental growth (GO:0048639),  response to glucocorticoid (GO:0051384),  positive regulation of protein phosphorylation (GO:0001934),  response to estrogen (GO:0043627),  positive regulation of glucose transport (GO:0010828),  positive regulation of lipid storage (GO:0010884),  chemotaxis (GO:0006935),  response to progesterone (GO:0032570),  positive regulation of G-protein coupled receptor protein signaling pathway (GO:0045745),  fatty acid metabolic process (GO:0006631),  response to magnesium ion (GO:0032026), |
| 6 | Cathelicidin antimicrobial peptide | Camp | extracellular space(GO:0005615),  cytoplasm(GO:0005737),  cell projection(GO:0042995) | cysteine-type endopeptidase inhibitor activity (GO:0004869) | positive regulation of cell proliferation (GO:0008284),  cellular response to lipopolysaccharide (GO:0071222),  cellular response to peptidoglycan (GO:0071224),  positive regulation of angiogenesis (GO:0045766),  positive regulation of protein phosphorylation (GO:0001934),  cellular response to tumor necrosis factor (GO:0071356),  cellular response to interleukin-1(GO:0071347),  cellular response to interleukin-6(GO:0071354), |
| 7 | Cadherin-1 | Cdh1 | integral component of membrane  (GO:0016021),  extracellular exosome(GO:0070062),  plasma membrane(GO:0005886),  cytoplasm(GO:0005737),  focal adhesion(GO:0005925),  lateral plasma membrane(GO:0016328),  aggresome(GO:0016235),  perinuclear region of cytoplasm (GO:0048471),  flotillin complex(GO:0016600),  cytoplasmic side of plasma membrane  (GO:0009898),  catenin complex(GO:0016342),  apical junction complex(GO:0043296),  endosome(GO:0005768),  cell-cell adherens junction(GO:0005913),  trans-Golgi network(GO:0005802),  lamellipodium(GO:0030027),  cortical actin cytoskeleton(GO:0030864) | calcium ion binding (GO:0005509) | positive regulation of transcription, DNA-templated(GO:0045893),  neuron projection development(GO:0031175),  homophilic cell adhesion via plasma membrane adhesion molecules(GO:0007156),  cellular response to indole-3-methanol(GO:0071681),  response to organic substance(GO:0010033),  single organismal cell-cell adhesion(GO:0016337),  positive regulation of transcription factor import into nucleus(GO:0042993),  negative regulation of cell-cell adhesion(GO:0022408),  synapse assembly(GO:0007416),  establishment of protein localization to plasma membrane(GO:0090002),  pituitary gland development(GO:0021983) |
| 8 | Collagen alpha-1(I) chain | Col1a1 | extracellular space (GO:0005615),  extracellular region (GO:0005576),  collagen type I trimer (GO:0005584),  Golgi apparatus (GO:0005794),  endoplasmic reticulum (GO:0005783),  secretory granule (GO:0030141) | metal ion binding (GO:0046872),  extracellular matrix structural constituent (GO:0005201) | protein transport (GO:0015031),  positive regulation of transcription, DNA-templated (GO:0045893),  positive regulation of cell migration (GO:0030355),  response to corticosteroid(GO:0031960),  response to estradiol (GO:0032355),  collagen biosynthetic process (GO:0032964),  response to cAMP (GO:0051591),  response to mechanical stimulus (GO:0009612),  response to peptide hormone (GO:0043434),  cellular response to tumor necrosis factor (GO:0071356),  osteoblast differentiation (GO:0001649),  bone trabecula formation (GO:0060346),  cellular response to mechanical stimulus (GO:00712600),  skin morphogenesis (GO:0043589),  negative regulation of cell-substrate adhesion (GO:0010812),  cartilage development involved in endochondral bone morphogenesis (GO:0060351),  cellular response to transforming growth factor beta stimulus (GO:0071560),  Ossification (GO:0001503),  response to hydrogen peroxide (GO:0042542),  response to steroid hormone (GO:0048545),  positive regulation of canonical Wnt signaling pathway (GO:0090263),  cellular response to amino acid stimulus (GO:0071230),  protein heterotrimerization (GO:0070208),  response to hyperoxia (GO:0055093),  cellular response to fibroblast growth factor stimulus (GO:0044344),  collagen fibril organization (GO:0030199),  blood vessel development (GO:0001568),  protein localization to nucleus (GO:0034504),  cellular response to epidermal growth factor stimulus (GO:0071364),  endochondral ossification (GO:0001958),  positive regulation of epithelial to mesenchymal transition (GO:0010718),  embryonic skeletal system development (GO:0048706),  face morphogenesis (GO:0060325),  Response to nutrient (G0:0007584) |
| 9 | Lysosomal alpha-glucosidase | Gaa | Lysosome (GO:0005764),  lysosomal membrane (GO:0005765) | maltose alpha-glucosidase activity (GO:0032450),  alpha-1,4-glucosidase activity (GO:0004558),  carbohydrate binding (GO:0030246) | glycogen catabolic process (GO:0005980) |
| 10 | Gamma-glutamyl hydrolase | Ggh | extracellular space (GO:0005615),  extracellular exosome (GO:0070062),  nucleus (GO:0005634),  lysosome (GO:0005764),  cytosol (GO:0005829),  melanosome(GO:0042470) | gamma-glutamyl-peptidase activity (GO:0034722) | Proteolysis (GO:0006508),  response to ethanol (GO:0045471),  response to insulin (GO:0032868),  glutamine metabolic process (GO:0006541) |
| 11 | Gamma-glutamyltranspeptidase 1 | Ggt1 | extracellular space (GO:0005615),  plasma membrane (GO:0005886),  integral component of plasma membrane  (GO:0005887) | glutathione hydrolase activity (GO:0036374),  gamma-glutamyltransferase activity (GO:0003840) | Aging (GO:0007568),  peptide modification (GO:0031179),  glutathione catabolic process (GO:0006751),  response to lipopolysaccharide (GO:0032496),  response to estradiol (GO:0032355),  glutamate metabolic process (GO:0006536),  glutathione biosynthetic process (GO:0006750),  cellular response to oxidative stress (GO:0034599),  response to tumor necrosis factor (GO:0034612) |
| 12 | Ig gamma-2B chain C region | Igh-1a | blood microparticle (GO:0072562),  external side of plasma membrane  (GO:0009897),  immunoglobulin complex, circulating  (GO:0042571) | antigen binding (GO:0003823),  immunoglobulin receptor binding (GO:0034987) | complement activation, classical pathway (GO:0006958),  positive regulation of B cell activation (GO:0050871),  B cell receptor signaling pathway (GO:0050853),  innate immune response (GO:0045087) |
| 13 | Lactoperoxidase (Predicted) | Lpo | extracellular space (GO:0005615),  extracellular exosome (GO:0070062),  cytoplasm(GO:0005737),  basolateral plasma membrane (GO:0016323) | thiocyanate peroxidase activity (GO:0036393),  heme binding (GO:0020037) | response to oxidative stress (GO:0006979) |
| 14 | Myosin-6 | Myh6 | Nucleoplasm (GO:0005654),  focal adhesion (GO:0005925),  myosin complex (GO:0016459),  stress fiber (GO:0001725),  Z disc (GO:0030018) | ATP binding (GO:0005524),  actin-dependent ATPase activity (GO:0030898),  motor activity (GO:0003774) | ATP metabolic process (GO:0046034),  regulation of heart rate (GO:0002027),  regulation of heart growth (GO:0060420),  adult heart development (GO:0007512),  ventricular cardiac muscle tissue morphogenesis (GO:0055010),  atrial cardiac muscle tissue morphogenesis (GO:0055009),  regulation of ATPase activity (GO:0043462),  cardiac muscle fiber development (GO:0048739),  BMP signaling pathway (GO:0030509),  canonical Wnt signaling pathway (GO:0060070),  cardiac muscle contraction (GO:0060048),  regulation of blood pressure (GO:0008217),  actin filament-based movement (GO:0030048) |
| 16 | Plasminogen activator, urokinase | Plau | extracellular space (GO:0005615),  extracellular exosome (GO:0070062),  focal adhesion (GO:0005925),  cell surface (GO:0009986) | serine-type endopeptidase activity (GO:0004252),  kinase activity (GO:0016301) | Fibrinolysis (GO:0042730),  regulation of cell proliferation (GO:0042127),  response to hypoxia (GO:0001666),  regulation of receptor activity (GO:0010469),  regulation of cell adhesion mediated by integrin (GO:0033628) |
| 17 | Plasminogen | Plg | plasma membrane (GO:0005886),  extracellular region (GO:0005576),  intracellular membrane-bounded organelle (GO:0043231),  extrinsic component of plasma membrane (GO:0019897) | serine-type endopeptidase activity (GO:0004252),  endopeptidase activity (GO:0004175) | Fibrinolysis (GO:0042730),  tissue remodeling (GO:0048771),  blood coagulation (GO:0007596),  proteolysis involved in cellular protein catabolic process (GO:0051603),  labyrinthine layer blood vessel development (GO:0060716),  trophoblast giant cell differentiation (GO:0060707) |
| 18 | Prostaglandin-H2 D-isomerase | Ptgds | extracellular space (GO:0005615),  extracellular exosome (GO:0070062),  extracellular region (GO:0005576),  Golgi apparatus (GO:0005794),  perinuclear region of cytoplasm,  (GO:0048471),  rough endoplasmic reticulum,  (GO:0005791),  nuclear envelope (GO:0005635),  nuclear membrane (GO:0031965) | transporter activity (GO:0005215),  prostaglandin-D synthase activity (GO:0004667),  retinoid binding (GO:0005501),  fatty acid binding (GO:0005504) | response to glucocorticoid (GO:0051384),  transport (GO:0006810),  prostaglandin biosynthetic process (GO:0001516) |
| 19 | Retinoid-inducible serine carboxypeptidase | Scpep1 | extracellular exosome (GO:0070062),  cytosol (GO:0005829) | serine-type carboxypeptidase activity (GO:0004185) | proteolysis involved in cellular protein catabolic process (GO:0051603),  positive regulation of vasodilation (GO:0045909),  negative regulation of blood pressure (GO:0045776) |
| 20 | Corticosteroid-binding globulin | Serpina6 | extracellular space (GO:0005615),  extracellular exosome (GO:0070062) | serine-type endopeptidase inhibitor activity (GO:0004867),  steroid binding (GO:0005496) | negative regulation of endopeptidase activity (GO:0010951),  glucocorticoid metabolic process (GO:0008211),  transport (GO:0006810) |
| 21 | Neutral and basic amino acid transport protein rBAT | Slc3a1 | extracellular exosome (GO:0070062),  plasma membrane (GO:0005886),  integral component of plasma membrane  (GO:0005887),  mitochondrial inner membrane  (GO:0005743),  vacuolar membrane (GO:0005774)  brush border membrane (GO:0031526) | cation binding (GO:0043169),  protein heterodimerization activity (GO:0046982),  catalytic activity (GO:0003824) | carbohydrate metabolic process (GO:0005975),  amino acid transport (GO:0006865) |
| 22 | Trefoil factor 1 | Tff1 | extracellular space (GO:0005615),  cytoplasm (GO:0005737) |  | negative regulation of cell proliferation (GO:0008285),  cell differentiation (GO:0030154),  response to peptide hormone (GO:0043434),  response to immobilization stress (GO:0035902) |

**Table S3** Enrichment analysis for molecular functions (MF) and biological process (BP).

| ONTOLOGY | ID | Description | GeneRatio | BgRatio | pvalue | p.adjust | qvalue | geneID | Count |
| --- | --- | --- | --- | --- | --- | --- | --- | --- | --- |
| MF | GO:0008238 | exopeptidase activity | '4/20 | 97/19711 | 2.51E-06 | 0.002081 | 0.001251 | Ggh/Scpep1/Ggt1/Anpep | 4 |
| MF | GO:0004866 | endopeptidase inhibitor activity | '4/20 | 191/19711 | 3.67E-05 | 0.008551 | 0.005142 | Ahsg/Ambp/C3/Serpina6 | 4 |
| MF | GO:0061135 | endopeptidase regulator activity | '4/20 | 198/19711 | 4.22E-05 | 0.008551 | 0.005142 | Ahsg/Ambp/C3/Serpina6 | 4 |
| MF | GO:0030414 | peptidase inhibitor activity | '4/20 | 207/19711 | 5.02E-05 | 0.008551 | 0.005142 | Ahsg/Ambp/C3/Serpina6 | 4 |
| MF | GO:0061134 | peptidase regulator activity | '4/20 | 235/19711 | 8.21E-05 | 0.011332 | 0.006814 | Ahsg/Ambp/C3/Serpina6 | 4 |
| MF | GO:0004857 | enzyme inhibitor activity | '4/20 | 400/19711 | 0.000626 | 0.027631 | 0.016615 | Ahsg/Ambp/C3/Serpina6 | 4 |
| MF | GO:0008236 | serine-type peptidase activity | '3/20 | 228/19711 | 0.001506 | 0.032806 | 0.019727 | Plau/Plg/Scpep1 | 3 |
| MF | GO:0017171 | serine hydrolase activity | '3/20 | 233/19711 | 0.001602 | 0.033462 | 0.020121 | Plau/Plg/Scpep1 | 3 |
| BP | GO:0042060 | wound healing | '5/20 | 421/19711 | 5.16E-05 | 0.008551 | 0.005142 | C3/Col1a1/Plau/Plg/Tff1 | 5 |
| BP | GO:0010951 | negative regulation of endopeptidase activity | '4/20 | 264/19711 | 0.000129 | 0.015224 | 0.009154 | Ahsg/Ambp/C3/Serpina6 | 4 |
| BP | GO:0046716 | muscle cell cellular homeostasis | '2/20 | 19/19711 | 0.000166 | 0.015258 | 0.009175 | Gaa/Plg | 2 |
| BP | GO:0010466 | negative regulation of peptidase activity | '4/20 | 282/19711 | 0.000166 | 0.015258 | 0.009175 | Ahsg/Ambp/C3/Serpina6 | 4 |
| BP | GO:0042730 | fibrinolysis | '2/20 | 21/19711 | 0.000203 | 0.016812 | 0.010109 | Plau/Plg | 2 |
| BP | GO:0043171 | peptide catabolic process | '2/20 | 30/19711 | 0.000418 | 0.027631 | 0.016615 | Ggt1/Anpep | 2 |
| BP | GO:0060249 | anatomical structure homeostasis | '4/20 | 362/19711 | 0.000429 | 0.027631 | 0.016615 | Ahsg/Gaa/Plg/Tff1 | 4 |
| BP | GO:0002026 | regulation of the force of heart contraction | '2/20 | 31/19711 | 0.000447 | 0.027631 | 0.016615 | Gaa/Myh6 | 2 |
| BP | GO:0045861 | negative regulation of proteolysis | '4/20 | 381/19711 | 0.000521 | 0.027631 | 0.016615 | Ahsg/Ambp/C3/Serpina6 | 4 |
| BP | GO:0045807 | positive regulation of endocytosis | '3/20 | 160/19711 | 0.000541 | 0.027631 | 0.016615 | Ahsg/B2m/C3 | 3 |
| BP | GO:0007596 | blood coagulation | '3/20 | 163/19711 | 0.000571 | 0.027631 | 0.016615 | C3/Plau/Plg | 3 |
| BP | GO:0007599 | hemostasis | '3/20 | 165/19711 | 0.000591 | 0.027631 | 0.016615 | C3/Plau/Plg | 3 |
| BP | GO:0050817 | coagulation | '3/20 | 169/19711 | 0.000634 | 0.027631 | 0.016615 | C3/Plau/Plg | 3 |
| BP | GO:0052548 | regulation of endopeptidase activity | '4/20 | 424/19711 | 0.000778 | 0.030639 | 0.018424 | Ahsg/Ambp/C3/Serpina6 | 4 |
| BP | GO:0046456 | icosanoid biosynthetic process | '2/20 | 42/19711 | 0.000822 | 0.030639 | 0.018424 | Ptgds/Ggt1 | 2 |
| BP | GO:0008217 | regulation of blood pressure | '3/20 | 196/19711 | 0.000974 | 0.030639 | 0.018424 | Myh6/Scpep1/Anpep | 3 |
| BP | GO:0010810 | regulation of cell-substrate adhesion | '3/20 | 197/19711 | 0.000989 | 0.030639 | 0.018424 | Col1a1/Plau/Plg | 3 |
| BP | GO:0052547 | regulation of peptidase activity | '4/20 | 456/19711 | 0.00102 | 0.030639 | 0.018424 | Ahsg/Ambp/C3/Serpina6 | 4 |
| BP | GO:0051346 | negative regulation of hydrolase activity | '4/20 | 460/19711 | 0.001054 | 0.030639 | 0.018424 | Ahsg/Ambp/C3/Serpina6 | 4 |
| BP | GO:0030195 | negative regulation of blood coagulation | '2/20 | 49/19711 | 0.001118 | 0.030639 | 0.018424 | Plau/Plg | 2 |
| BP | GO:1900047 | negative regulation of hemostasis | '2/20 | 49/19711 | 0.001118 | 0.030639 | 0.018424 | Plau/Plg | 2 |
| BP | GO:0001525 | angiogenesis | '4/20 | 469/19711 | 0.001133 | 0.030639 | 0.018424 | C3/Camp/Plau/Anpep | 4 |
| BP | GO:0002237 | response to molecule of bacterial origin | '4/20 | 469/19711 | 0.001133 | 0.030639 | 0.018424 | B2m/Camp/Plau/Ggt1 | 4 |
| BP | GO:0008015 | blood circulation | '4/20 | 475/19711 | 0.001187 | 0.030639 | 0.018424 | Gaa/Myh6/Scpep1/Anpep | 4 |
| BP | GO:0010038 | response to metal ion | '4/20 | 476/19711 | 0.001196 | 0.030639 | 0.018424 | B2m/C3/Ggh/Tff1 | 4 |
| BP | GO:0050819 | negative regulation of coagulation | '2/20 | 51/19711 | 0.00121 | 0.030639 | 0.018424 | Plau/Plg | 2 |
| BP | GO:0003013 | circulatory system process | '4/20 | 480/19711 | 0.001234 | 0.030639 | 0.018424 | Gaa/Myh6/Scpep1/Anpep | 4 |
| BP | GO:0055093 | response to hyperoxia | '2/20 | 52/19711 | 0.001258 | 0.030639 | 0.018424 | Col1a1/Plau | 2 |
| BP | GO:0010039 | response to iron ion | '2/20 | 53/19711 | 0.001307 | 0.030912 | 0.018588 | B2m/Tff1 | 2 |
| BP | GO:1901570 | fatty acid derivative biosynthetic process | '2/20 | 54/19711 | 0.001356 | 0.03119 | 0.018755 | Ptgds/Ggt1 | 2 |
| BP | GO:0010812 | negative regulation of cell-substrate adhesion | '2/20 | 56/19711 | 0.001458 | 0.032619 | 0.019614 | Col1a1/Plg | 2 |
| BP | GO:0036296 | response to increased oxygen levels | '2/20 | 59/19711 | 0.001617 | 0.033462 | 0.020121 | Col1a1/Plau | 2 |
| BP | GO:0031638 | zymogen activation | '2/20 | 60/19711 | 0.001671 | 0.033751 | 0.020295 | Plau/Ggt1 | 2 |
| BP | GO:0050766 | positive regulation of phagocytosis | '2/20 | 61/19711 | 0.001727 | 0.034043 | 0.020471 | Ahsg/C3 | 2 |
| BP | GO:0032355 | response to estradiol | '3/20 | 246/19711 | 0.001871 | 0.035618 | 0.021418 | C3/Col1a1/Ggt1 | 3 |
| BP | GO:0034612 | response to tumor necrosis factor | '3/20 | 247/19711 | 0.001893 | 0.035618 | 0.021418 | Camp/Col1a1/Ggt1 | 3 |
| BP | GO:0016485 | protein processing | '3/20 | 256/19711 | 0.002096 | 0.037708 | 0.022675 | C3/Plau/Ggt1 | 3 |
| BP | GO:0030100 | regulation of endocytosis | '3/20 | 257/19711 | 0.002119 | 0.037708 | 0.022675 | Ahsg/B2m/C3 | 3 |
| BP | GO:0061045 | negative regulation of wound healing | '2/20 | 68/19711 | 0.00214 | 0.037708 | 0.022675 | Plau/Plg | 2 |
| BP | GO:0051851 | modification by host of symbiont morphology or physiology | '2/20 | 73/19711 | 0.002462 | 0.042467 | 0.025536 | Camp/Plg | 2 |
| BP | GO:0030193 | regulation of blood coagulation | '2/20 | 78/19711 | 0.002805 | 0.045533 | 0.02738 | Plau/Plg | 2 |
| BP | GO:0051702 | interaction with symbiont | '2/20 | 78/19711 | 0.002805 | 0.045533 | 0.02738 | Camp/Plg | 2 |
| BP | GO:1900046 | regulation of hemostasis | '2/20 | 78/19711 | 0.002805 | 0.045533 | 0.02738 | Plau/Plg | 2 |
| BP | GO:0042246 | tissue regeneration | '2/20 | 80/19711 | 0.002948 | 0.046936 | 0.028223 | Plau/Plg | 2 |
| BP | GO:0031099 | regeneration | '3/20 | 291/19711 | 0.003014 | 0.047085 | 0.028314 | Ahsg/Plau/Plg | 3 |
| BP | GO:0050818 | regulation of coagulation | '2/20 | 82/19711 | 0.003094 | 0.047442 | 0.028528 | Plau/Plg | 2 |
| BP | GO:1903035 | negative regulation of response to wounding | '2/20 | 84/19711 | 0.003244 | 0.048835 | 0.029365 | Plau/Plg | 2 |

**Table S4 Gene functions by GENMANIA analysis.**

| **Symbol** | **Score** | **Functions** | **Links** |
| --- | --- | --- | --- |
| Ggt1 | 0.941681 | exopeptidase activity, peptidase activity, peptidase  activity, acting on L-amino acid peptides, response  to estradiol, response to estrogen, response to  lipopolysaccharide | http://www.ensembl.org/Rattus_norvegicus/geneview?gene=ENSRNOG00000047697 |
| Camp | 0.906338 | cell killing, disruption of cells of other organism, disruption of cells of other organism involved in symbiotic interaction, killing of cells in other organism involved in symbiotic interaction, killing of cells of other organism, regulation of angiogenesis, regulation of vasculature development, response to lipopolysaccharide | http://www.ncbi.nlm.nih.gov/sites/entrez?db=gene&cmd=search&term=316010 |
| Cdh1 | 0.834549 | epithelial cell proliferation, negative regulation of cell adhesion, regulation of body fluid levels, regulation of cell adhesion | http://www.ncbi.nlm.nih.gov/sites/entrez?db=gene&cmd=search&term=83502 |
| Lpo | 0.833314 | antioxidant activity, oxidoreductase activity, acting on peroxide as acceptor, peroxidase activity | http://www.ncbi.nlm.nih.gov/sites/entrez?db=gene&cmd=search&term=287610 |
| Scpep1 | 0.803332 | exopeptidase activity, peptidase activity, peptidase activity, acting on L-amino acid peptides | http://www.ncbi.nlm.nih.gov/sites/entrez?db=gene&cmd=search&term=114861 |
| Myh6 | 0.668362 |  | http://www.ncbi.nlm.nih.gov/sites/entrez?db=gene&cmd=search&term=29556 |
| Anpep | 0.644182 | exopeptidase activity, peptidase activity, peptidase activity, acting on L-amino acid peptides | http://www.ncbi.nlm.nih.gov/sites/entrez?db=gene&cmd=search&term=81641 |
| Ahsg | 0.605664 | acute inflammatory response, anatomical structure homeostasis, blood microparticle, enzyme inhibitor activity, organ regeneration, regeneration, tissue homeostasis | http://www.ncbi.nlm.nih.gov/sites/entrez?db=gene&cmd=search&term=25373 |
| Ptgds | 0.603057 | response to corticosteroid | http://www.ncbi.nlm.nih.gov/sites/entrez?db=gene&cmd=search&term=25526 |
| B2m | 0.597292 | anatomical structure homeostasis, cell killing, positive regulation of adaptive immune response, positive regulation of adaptive immune response based on somatic recombination of immune receptors built from immunoglobulin superfamily domains, positive regulation of leukocyte mediated immunity, positive regulation of lymphocyte mediated immunity, regulation of immune effector process, tissue homeostasis | http://www.ncbi.nlm.nih.gov/sites/entrez?db=gene&cmd=search&term=24223 |
| Col1a1 | 0.594872 | negative regulation of cell adhesion, regulation of cell adhesion, response to corticosteroid, response to estradiol, response to estrogen, response to nutrient, wound healing | http://www.ncbi.nlm.nih.gov/sites/entrez?db=gene&cmd=search&term=29393 |
| Plau | 0.594817 | blood coagulation, coagulation, epithelial cell proliferation, fibrinolysis, hemostasis, negative regulation of blood coagulation, negative regulation of coagulation, negative regulation of hemostasis, peptidase activity, regeneration, regulation of blood coagulation, regulation of body fluid levels, regulation of cell adhesion, regulation of coagulation, regulation of hemostasis, regulation of wound healing, response to carbohydrate, response to lipopolysaccharide, response to monosaccharide, tissue regeneration, wound healing | http://www.ncbi.nlm.nih.gov/sites/entrez?db=gene&cmd=search&term=25619 |
| Ggh | 0.589884 | exopeptidase activity, peptidase activity, peptidase activity, acting on L-amino acid peptides, response to transition metal nanoparticle | http://www.ncbi.nlm.nih.gov/sites/entrez?db=gene&cmd=search&term=25455 |
| Slc3a1 | 0.584577 |  | http://www.ncbi.nlm.nih.gov/sites/entrez?db=gene&cmd=search&term=29484 |
| Gaa | 0.570352 | anatomical structure homeostasis, maintenance of location, muscle cell cellular homeostasis | http://www.ncbi.nlm.nih.gov/sites/entrez?db=gene&cmd=search&term=367562 |
| C3 | 0.558915 | acute inflammatory response, acylglycerol metabolic process, blood coagulation, blood microparticle, coagulation, glycerolipid metabolic process, hemostasis, humoral immune response mediated by circulating immunoglobulin, maintenance of location, neutral lipid metabolic process, positive regulation of adaptive immune response, positive regulation of adaptive immune response based on somatic recombination of immune receptors built from immunoglobulin superfamily domains, positive regulation of B cell mediated immunity, positive regulation of humoral immune response, positive regulation of immunoglobulin mediated immune response, positive regulation of leukocyte mediated immunity, positive regulation of lymphocyte mediated immunity, regulation of angiogenesis, regulation of body fluid levels, regulation of immune effector process, regulation of vasculature development, response to corticosteroid, response to estradiol, response to estrogen, triglyceride metabolic process, wound healing | http://www.ncbi.nlm.nih.gov/sites/entrez?db=gene&cmd=search&term=24232 |
| Ambp | 0.538564 | blood microparticle, enzyme inhibitor activity, negative regulation of hydrolase activity, serine-type endopeptidase inhibitor activity | http://www.ncbi.nlm.nih.gov/sites/entrez?db=gene&cmd=search&term=25377 |
| Serpina6 | 0.534669 | enzyme inhibitor activity, negative regulation of hydrolase activity, serine-type endopeptidase inhibitor activity, steroid binding, steroid metabolic process | http://www.ncbi.nlm.nih.gov/sites/entrez?db=gene&cmd=search&term=299270 |
| Plg | 0.507846 | anatomical structure homeostasis, blood coagulation, blood microparticle, coagulation, fibrinolysis, hemostasis, muscle cell cellular homeostasis, negative regulation of blood coagulation, negative regulation of cell adhesion, negative regulation of coagulation, negative regulation of hemostasis, peptidase activity, peptidase activity, acting on L-amino acid peptides, positive regulation of blood coagulation, positive regulation of hemostasis, regeneration, regulation of blood coagulation, regulation of body fluid levels, regulation of cell adhesion, regulation of coagulation, regulation of fibrinolysis, regulation of hemostasis, regulation of wound healing, tissue regeneration, wound healing | http://www.ncbi.nlm.nih.gov/sites/entrez?db=gene&cmd=search&term=85253 |
| Tff1 | 0.48653 | anatomical structure homeostasis, digestive system process, response to estradiol, response to estrogen, tissue homeostasis, wound healing | http://www.ncbi.nlm.nih.gov/sites/entrez?db=gene&cmd=search&term=117270 |

**Table S5 Gene functions by GENMANIA analysis (Continued).**

| **Function** | **FDR** | **Genes in network** | **Genes in genome** |
| --- | --- | --- | --- |
| high-density lipoprotein particle | 7.02E-07 | 5 | 15 |
| response to estrogen | 6.31615E-05 | 8 | 248 |
| response to estradiol | 0.000792702 | 6 | 161 |
| exopeptidase activity | 0.001759548 | 4 | 48 |
| response to lipopolysaccharide | 0.009745826 | 6 | 298 |
| peptidase activity | 0.009745826 | 6 | 297 |

**Table S6 Top 10 Gene to Gene relationship** **by GENMANIA analysis.**

| **Gene 1** | **Gene 2** | **Weight** | **Network group** |
| --- | --- | --- | --- |
| Ggt6 | Ggt1 | 0.106063 | Shared protein domains |
| Ggt6 | Ggt1 | 0.5 | Shared protein domains |
| Ggt7 | Ggt1 | 0.106063 | Shared protein domains |
| Ggt7 | Ggt6 | 0.106063 | Shared protein domains |
| Ggt7 | Ggt1 | 0.5 | Shared protein domains |
| Ggt7 | Ggt6 | 0.5 | Shared protein domains |
| Alb | Ahsg | 0.018864 | Co-expression |
| Alb | C3 | 0.017446 | Co-expression |
| Alb | Ambp | 0.016637 | Co-expression |
| Alb | Plg | 0.007836 | Co-expression |
| Alb | C3 | 0.013205 | Co-expression |
| Alb | Ambp | 0.013227 | Co-expression |
| Alb | Serpina6 | 0.014009 | Co-expression |
| Alb | Plg | 0.01262 | Co-expression |
| Alb | C3 | 0.012138 | Co-expression |
| Alb | Ambp | 0.015338 | Co-expression |
| Alb | Serpina6 | 0.013865 | Co-expression |
| Alb | Serpina6 | 0.007034 | Co-expression |
| Alb | Ahsg | 0.017574 | Co-expression |
| Alb | Ambp | 0.020328 | Co-expression |
| Alb | Serpina6 | 0.020056 | Co-expression |
| Alb | Plg | 0.012023 | Co-expression |
| Alb | C3 | 0.016334 | Co-expression |
| Alb | Ambp | 0.012921 | Co-expression |
| Alb | Ahsg | 0.020527 | Co-expression |
| Alb | C3 | 0.025518 | Co-expression |
| Alb | Ambp | 0.016605 | Co-expression |
| Alb | Ahsg | 0.015739 | Co-expression |
| Alb | Ambp | 0.010943 | Co-expression |
| Alb | Serpina6 | 0.010441 | Co-expression |
| Alb | Plg | 0.012999 | Co-expression |
| Alb | Serpina6 | 0.01273 | Co-expression |
| Alb | Ahsg | 0.027527 | Co-expression |
| Alb | Ambp | 0.015758 | Co-expression |
| Alb | Tff1 | 0.004653 | Co-localization |
| Ambp | Ahsg | 0.018423 | Co-expression |
| Ambp | C3 | 0.017286 | Co-expression |
| Ambp | Ahsg | 0.013201 | Co-expression |
| Ambp | C3 | 0.005404 | Co-expression |
| Ambp | Ahsg | 0.016078 | Co-expression |
| Ambp | C3 | 0.00712 | Co-expression |
| Ambp | Ahsg | 0.017263 | Co-expression |
| Ambp | Ahsg | 0.011256 | Co-expression |
| Ambp | B2m | 0.006192 | Co-expression |
| Ambp | C3 | 0.007278 | Co-expression |
| Ambp | Ahsg | 0.012506 | Co-expression |
| Ambp | C3 | 0.01619 | Co-expression |
| Ambp | Ahsg | 0.01321 | Co-expression |
| Ambp | C3 | 0.009222 | Co-expression |
| Ambp | Ptgds | 0.023649 | Shared protein domains |
| Ambp | Ptgds | 0.013891 | Shared protein domains |
| Anpep | Cdh1 | 0.020638 | Co-expression |
| B2m | Ptgds | 0.004566 | Co-expression |
| B2m | Igh-1a | 0.019513 | Shared protein domains |
| B2m | Igh-1a | 0.020804 | Shared protein domains |
| C3 | Ahsg | 0.019542 | Co-expression |
| C3 | Ahsg | 0.01309 | Co-expression |
| C3 | Ahsg | 0.012705 | Co-expression |
| C3 | Ahsg | 0.017776 | Co-expression |
| C3 | Ahsg | 0.015876 | Co-expression |
| C3 | Col1a1 | 0.011504 | Co-expression |
| C3 | B2m | 0.018893 | Co-expression |
| C3 | B2m | 0.014057 | Co-expression |
| Col1a1 | B2m | 0.02051 | Co-expression |
| Col1a1 | Ptgds | 0.004917 | Co-expression |
| Col1a1 | Scpep1 | 0.00578 | Co-expression |
| Gaa | Ptgds | 0.00516 | Co-expression |
| Gaa | Slc3a1 | 0.010127 | Shared protein domains |
| Gc | Ahsg | 0.016676 | Co-expression |
| Gc | Plau | 0.013381 | Co-expression |
| Gc | C3 | 0.016056 | Co-expression |
| Gc | Ambp | 0.014788 | Co-expression |
| Gc | Plg | 0.006661 | Co-expression |
| Gc | Alb | 0.015963 | Co-expression |
| Gc | Ahsg | 0.014848 | Co-expression |
| Gc | C3 | 0.006052 | Co-expression |
| Gc | Ambp | 0.006106 | Co-expression |
| Gc | Serpina6 | 0.006252 | Co-expression |
| Gc | Alb | 0.014888 | Co-expression |
| Gc | Ahsg | 0.014557 | Co-expression |
| Gc | C3 | 0.006519 | Co-expression |
| Gc | Ambp | 0.008089 | Co-expression |
| Gc | Serpina6 | 0.00728 | Co-expression |
| Gc | Alb | 0.014017 | Co-expression |
| Gc | Ggh | 0.017682 | Co-expression |
| Gc | Ahsg | 0.017528 | Co-expression |
| Gc | Plg | 0.016152 | Co-expression |
| Gc | Ahsg | 0.014205 | Co-expression |
| Gc | C3 | 0.010727 | Co-expression |
| Gc | Ambp | 0.005834 | Co-expression |
| Gc | Serpina6 | 0.008907 | Co-expression |
| Gc | Tff1 | 0.004407 | Co-expression |
| Gc | Alb | 0.01317 | Co-expression |
| Gc | C3 | 0.029065 | Co-expression |
| Gc | Ambp | 0.016958 | Co-expression |
| Gc | Alb | 0.027561 | Co-expression |
| Gc | Ahsg | 0.013146 | Co-expression |
| Gc | Ambp | 0.010243 | Co-expression |
| Gc | Serpina6 | 0.010146 | Co-expression |
| Gc | Plg | 0.008467 | Co-expression |
| Gc | Alb | 0.010849 | Co-expression |
| Gc | Ahsg | 0.01936 | Co-expression |
| Gc | Ambp | 0.012053 | Co-expression |
| Gc | Anpep | 0.013028 | Co-expression |
| Gc | B2m | 0.010225 | Co-expression |
| Gc | Slc3a1 | 0.006838 | Co-expression |
| Gc | Plau | 0.010678 | Co-localization |
| Gc | Slc3a1 | 0.013554 | Co-localization |
| Gc | Alb | 0.205487 | Shared protein domains |
| Gc | Alb | 0.25 | Shared protein domains |
| Myh6 | Lpo | 0.011527 | Co-expression |
| Plg | Ahsg | 0.006891 | Co-expression |
| Plg | C3 | 0.006431 | Co-expression |
| Plg | Ambp | 0.006456 | Co-expression |
| Plg | C3 | 0.005009 | Co-expression |
| Plg | Ambp | 0.004985 | Co-expression |
| Plg | Serpina6 | 0.005363 | Co-expression |
| Plg | C3 | 0.005318 | Co-expression |
| Plg | Ahsg | 0.014061 | Co-expression |
| Plg | Ambp | 0.01147 | Co-expression |
| Plg | Ahsg | 0.013441 | Co-expression |
| Plg | Ambp | 0.008622 | Co-expression |
| Plg | Serpina6 | 0.008508 | Co-expression |
| Plg | Ahsg | 0.00568 | Co-expression |
| Plg | C3 | 0.005187 | Co-expression |
| Plg | Ambp | 0.003905 | Co-expression |
| Plg | Ahsg | 0.006843 | Co-expression |
| Plg | Serpina6 | 0.002631 | Co-localization |
| Plg | Plau | 0.01458 | Shared protein domains |
| Plg | Plau | 0.014892 | Shared protein domains |
| Serpina6 | Ahsg | 0.013996 | Co-expression |
| Serpina6 | C3 | 0.005635 | Co-expression |
| Serpina6 | Ambp | 0.005599 | Co-expression |
| Serpina6 | Ahsg | 0.014283 | Co-expression |
| Serpina6 | C3 | 0.006364 | Co-expression |
| Serpina6 | Ambp | 0.008101 | Co-expression |
| Serpina6 | Ahsg | 0.006455 | Co-expression |
| Serpina6 | Ahsg | 0.016732 | Co-expression |
| Serpina6 | Ambp | 0.020414 | Co-expression |
| Serpina6 | C3 | 0.01191 | Co-expression |
| Serpina6 | Ahsg | 0.017395 | Co-expression |
| Serpina6 | Ahsg | 0.012708 | Co-expression |
| Serpina6 | Ambp | 0.009934 | Co-expression |
| Serpina6 | Myh6 | 0.022052 | Co-expression |
| Slc3a1 | Anpep | 0.015775 | Co-expression |
| Slc3a1 | Col1a1 | 0.013424 | Co-expression |
| Slc3a1 | Anpep | 0.016406 | Co-expression |
| Slc3a1 | Plau | 0.010248 | Co-expression |
| Slc3a1 | Anpep | 0.022114 | Co-expression |
| Slc3a1 | Anpep | 0.010525 | Co-expression |
| Slc3a1 | Plau | 0.006383 | Co-localization |
| Slc3a1 | Ggh | 0.010775 | Co-localization |
| Tff1 | Myh6 | 0.010872 | Co-expression |
| Tff1 | Plg | 0.010338 | Co-expression |
| Tff1 | Myh6 | 0.009644 | Co-expression |
| Tff1 | Ahsg | 0.015607 | Co-expression |
| Tff1 | Plg | 0.008436 | Co-expression |
| Tff1 | Ahsg | 0.004435 | Co-expression |
| Tff1 | Plg | 0.001188 | Co-expression |
| Tff1 | Ahsg | 0.0066 | Co-localization |
| Tff1 | Serpina6 | 0.00182 | Co-localization |
| Tff1 | Plg | 0.002334 | Co-localization |
| Tff1 | Gaa | 0.059058 | Shared protein domains |
| Tff1 | Gaa | 0.098556 | Shared protein domains |

**Table S7** Top 5 associated KEGG network analysis based on protein list.

| Pathway Name | Match Status | p | -log(p) | Holm p | FDR | Impact |
| --- | --- | --- | --- | --- | --- | --- |
| Complement and coagulation cascades | 3/67 | 3.68E-04 | 7.9063 | 0.078474 | 0.078474 | 0.46296 |
| Glutathione metabolism | 2/51 | 0.00541 | 5.2195 | 1 | 0.38413 | 0.86364 |
| Arachidonic acid metabolism | 2/82 | 0.013564 | 4.3003 | 1 | 0.7223 | 0.05 |
| Taurine and hypotaurine metabolism | 1/9 | 0.019794 | 3.9224 | 1 | 0.84323 | 0.33333 |
| Cardiac muscle contraction | 1/12 | 0.026312 | 3.6377 | 1 | 0.86647 | 0.36364 |

**Table S8** Enriched gene-metabolite pathways based on proteomic-metabolomic joint pathway analysis by MetaboAnalyst platform.

| **Pathway Name** | **Match Status** | **p** | **-log(p)** | **Holm p** | **FDR** | **Impact** |
| --- | --- | --- | --- | --- | --- | --- |
| Glutathione metabolism | 2/51 | 0.001275 | 6.6649 | 0.27156 | 0.27156 | 0.86364 |
| Taurine and hypotaurine metabolism | 1/9 | 0.009941 | 4.6111 | 1 | 1 | 0.33333 |
| Pentose phosphate pathway | 1/26 | 0.028494 | 3.5581 | 1 | 1 | 0.5 |
| Fructose and mannose metabolism | 1/37 | 0.040344 | 3.2103 | 1 | 1 | 0.38889 |
| Complement and coagulation cascades | 1/67 | 0.07205 | 2.6304 | 1 | 1 | 0.055556 |
| Adherens junction | 1/70 | 0.075172 | 2.588 | 1 | 1 | 0.125 |
| Glycolysis / Gluconeogenesis | 1/79 | 0.084486 | 2.4712 | 1 | 1 | 0.18182 |
| Arachidonic acid metabolism | 1/82 | 0.087573 | 2.4353 | 1 | 1 | 0.025 |
| Cell adhesion molecules (CAMs) | 1/148 | 0.15333 | 1.8752 | 1 | 1 | 0.014563 |
| Tuberculosis | 1/186 | 0.18936 | 1.6641 | 1 | 1 | 0.010309 |

**Table S9** GSH/GSSG ratio, and peptide MRM quantification results.(n=3, mean ± SEM)

| **MRM** | **GSH/GSSG ratio** | **Ggt1/200****µg urinary protein** | **Anpep/200µg** **urinary protein** |
| --- | --- | --- | --- |
| Control | 7.173±0.376 | 0.018±0.001 | 0.289±0.020 |
| OVX | 3.178±0.638 | 0.030±0.001^***^ | 0.172±0.013^***^ |
| HP30 | 7.255±0.432 | 0.015±0.001^NS, ##^ | 0.206±0.027 ^NS, ##^ |
| HP10 | 6.018±0.810 | 0.020±0.001^**, NS^ | 0.183±0.011^**, NS^ |

^*^p < 0.05, ^**^p < 0.005, ^***^p < 0.0005 versus control rats; ^#^p < 0.05, ^##^p < 0.005, ^###^p < 0.0005 versus OVX rats. NS, not significant.

**Table S10** Endo-metabolites MRM quantification results (n=6, mean ± SD).

| **Urinary metabolites** | **weeks** | **Control** | **OVX** | **HPH** | **HPL** |
| --- | --- | --- | --- | --- | --- |
| L-Glutamate | 3 | 1.19±0.018 | 1.36±0.003^NS^ | 2.46±0.133^***,###^ | 2.12±0.162^***,###^ |
|  | 6 | 1.94±0.035 | 3.51±0.093^***^ | 2.20±0.105^NS,###^ | 3.14±0.154^***,NS^ |
|  | 12 | 0.39±0.045 | 4.12±0.565^***^ | 0.13±0.015 ^NS,###^ | 0.21±0.010 ^NS,###^ |
| L-Glutamine | 3 | 17.71±0.627 | 25.91±0.435^***^ | 27.28±1.781^***,NS^ | 22.19±0.850^NS,NS^ |
|  | 6 | 11.12±0.102 | 22.69±0.308^***^ | 12.78±0.758 ^NS,###^ | 21.19±0.832^***,NS^ |
|  | 12 | 3.16±0.423 | 24.64±3.260^***^ | 1.44±0.072 ^NS,###^ | 2.07±0.022 ^NS,###^ |
| Pyroglutamic acid | 3 | 7.37±1.325 | 33.13±1.353^***^ | 21.52±2.105^***,###^ | 24.83±2.721^***,#^ |
|  | 6 | 12.38±0.241 | 22.39±0.579^***^ | 16.63±1.264^*,###^ | 28.15±1.421^***,##^ |
|  | 12 | 1.84±0.082 | 10.86±2.685^***^ | 2.68±0.189 ^NS,###^ | 2.82±0.389 ^NS,##^ |
| L-Cysteine | 3 | 6.15±0.562 | 1.87±0.146^***^ | 5.51±0.823^NS,NS^ | 3.23±0.909^NS,NS^ |
|  | 6 | 4.11±0.495 | 1.72±0.185^**^ | 5.86±0.531 ^NS,###^ | 5.09±0.665 ^NS,###^ |
|  | 12 | 6.59±0.686 | 3.18±0.357^***^ | 5.66±0.509 ^NS,#^ | 4.31±0.605 ^NS,NS^ |
| L-Cysteamine | 3 | 3.31±0.135 | 3.43±0.146 ^NS^ | 3.26±0.105 ^NS,NS^ | 2.79±0.150 ^NS,NS^ |
|  | 6 | 4.24±0.143 | 2.88±0.062^***^ | 3.14±0.158^***,NS^ | 4.81±0.216 ^NS,###^ |
|  | 12 | 3.17±0.191 | 0.84±0.246^***^ | 3.44±0.110^NS,###^ | 3.19±0.243^NS,###^ |
| L-Glycine | 3 | 1.27±0.039 | 1.07±0.010^**^ | 1.27±0.039^NS,##^ | 1.24±0.033^NS,#^ |
|  | 6 | 1.11±0.005 | 1.08±0.011^**^ | 1.13±0.005^NS,###^ | 1.09±0.004 ^NS,NS^ |
|  | 12 | 1.34±0.050 | 0.92±0.007^***^ | 1.03±0.014 ^NS,#^ | 0.93±0.003 ^*,NS^ |
| Taurine | 3 | 126.42±4.237 | 44.05±2.541^***^ | 67.55±3.656^***,###^ | 55.88±0.603^***,NS^ |
|  | 6 | 105.39±3.123 | 33.69±1.481^***^ | 94.45±0.647^**,###^ | 57.2±0.341^***,###^ |
|  | 12 | 100.84±2.202 | 22.67±2.279^***^ | 102.08±0.799 ^NS,###^ | 88.35±0.809^***,###^ |
| Serum | | | | | |
| L-Glutamate | 12 | 0.53±0.022 | 0.86±0.009^***^ | 0.57±0.010 ^NS,###^ | 0.61±0.019^**,###^ |
| L-Glutamine | 12 | 3.41±0.145 | 4.93±0.141^***^ | 3.39±0.415 ^NS,###^ | 4.07±0.091 ^NS,###^ |
| Pyroglutamic acid | 12 | 6.32±0.569 | 10.41±0.803^***^ | 7.61±0.766 ^NS,###^ | 7.85±0.533 ^NS,#^ |
| L-Cysteine | 12 | 1.28±0.025 | 1.14±0.042^*^ | 1.29±0.033 ^NS,##^ | 1.24±0.030 ^NS,NS^ |
| L-Cysteamine | 12 | 33.42±3.859 | 18.24±0.001^**^ | 32.18±2.804^NS,##^ | 44.32±2.974^NS,##^ |
| L-Glycine | 12 | 1.68±0.072 | 0.89±0.056^***^ | 1.84±0.080 ^NS,###^ | 1.33±0.062 ^**,###^ |
| Taurine | 12 | 3.94±0.134 | 0.08±0.003^***^ | 4.12±0.116 ^NS,###^ | 4.12±0.234 ^NS,###^ |

^*^p < 0.05, ^**^p < 0.005, ^***^p < 0.0005 *versus* control rats; ^#^p < 0.05, ^##^p < 0.005, ^###^p < 0.0005 *versus* OVX rats. NS, not significant.

**Table S11** Immunohistochemistry scan, Western blot and assay results (n=3, mean ± SEM).

| **IHC AOD ratio** | **ERα (Uterus)** | **ERβ (Uterus)** | **Ggt1 (Kidney)** | **Anpep (Kidney)** |
| --- | --- | --- | --- | --- |
| Control | 0.017±0.002 | 0.047±0.005 | 0.009±0.003 | 0.032±0.005 |
| OVX | 0.008±0.002^***^ | 0.001±0.0001^***^ | 0.026±0.003^*^ | 0.003±0.001^***^ |
| HPL-H | 0.019±0.002^NS, ##^ | 0.013±0.001^***, #^ | 0.011±0.002^NS, #^ | 0.024±0.004 ^NS, ##^ |
| HPL-L | 0.010±0.0003^**, NS^ | 0.012±0.0015^***, #^ | 0.016±0.0082^NS, NS^ | 0.012±0.0016^**, NS^ |
| **WB assay** | **ERα (Uterus)** | **ERβ (Uterus)** | **Ggt1 (Kidney)** | **Anpep (Kidney)** |
| Control | 0.497±0.024 | 0.569±0.030 | 0.195±0.039 | 0.476±0.036 |
| OVX | 0.258±0.124* | 0.206±0.042* | 0.470±0.105* | 0.264±0.024* |
| HPL-H | 0.483±0.058^NS, NS^ | 0.409±0.045^*, #^ | 0.342±0.098 ^NS, NS^ | 0.439±0.055 ^NS, #^ |
| HPL-L | 0.449±0.045^NS, NS^ | 0.388±0.024^*, ##^ | 0.198±0.046 ^NS, #^ | 0.420±0.064 ^NS, #^ |
| **qRT-PCR analysis** | / | / | **Ggt1 (Kidney)** | **Anpep (Kidney)** |
| Control | / | / | 1.003±0.098 | 1.007±0.038 |
| OVX | / | / | 1.683±0.289 | 0.840±0.051 |
| HPL-H | / | / | 0.790±0.023 | 1.257±0.194 |
| HPL-L | / | / | 0.877±0.090 | 1.013±0.040 |

^*^p < 0.05, ^**^p < 0.005, ^***^p < 0.0005 versus control rats; ^#^p < 0.05, ^##^p < 0.005, ^###^p < 0.0005 versus. NS, not significant.

^&^ Immunohistochemistry (IHC) average optional density (AOD) ratio：DAB staining as indicated by brown color in immunohistochemistry analysis was interpreted as positive expression. Image-Pro Plus 6.0 software was used to measure the IHC DAB staining area to obtain the integrated optical density (IOD) and staining area (Area) values. Then the AOD ratio indicates the ratio of IOD to Area, that a larger AOD ratio represent a higher protein expression level.


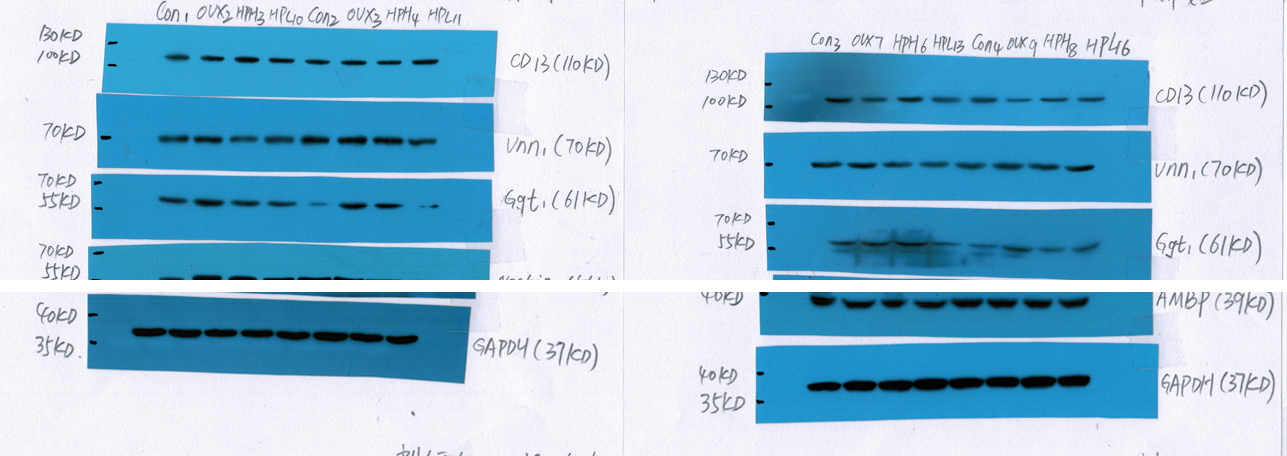


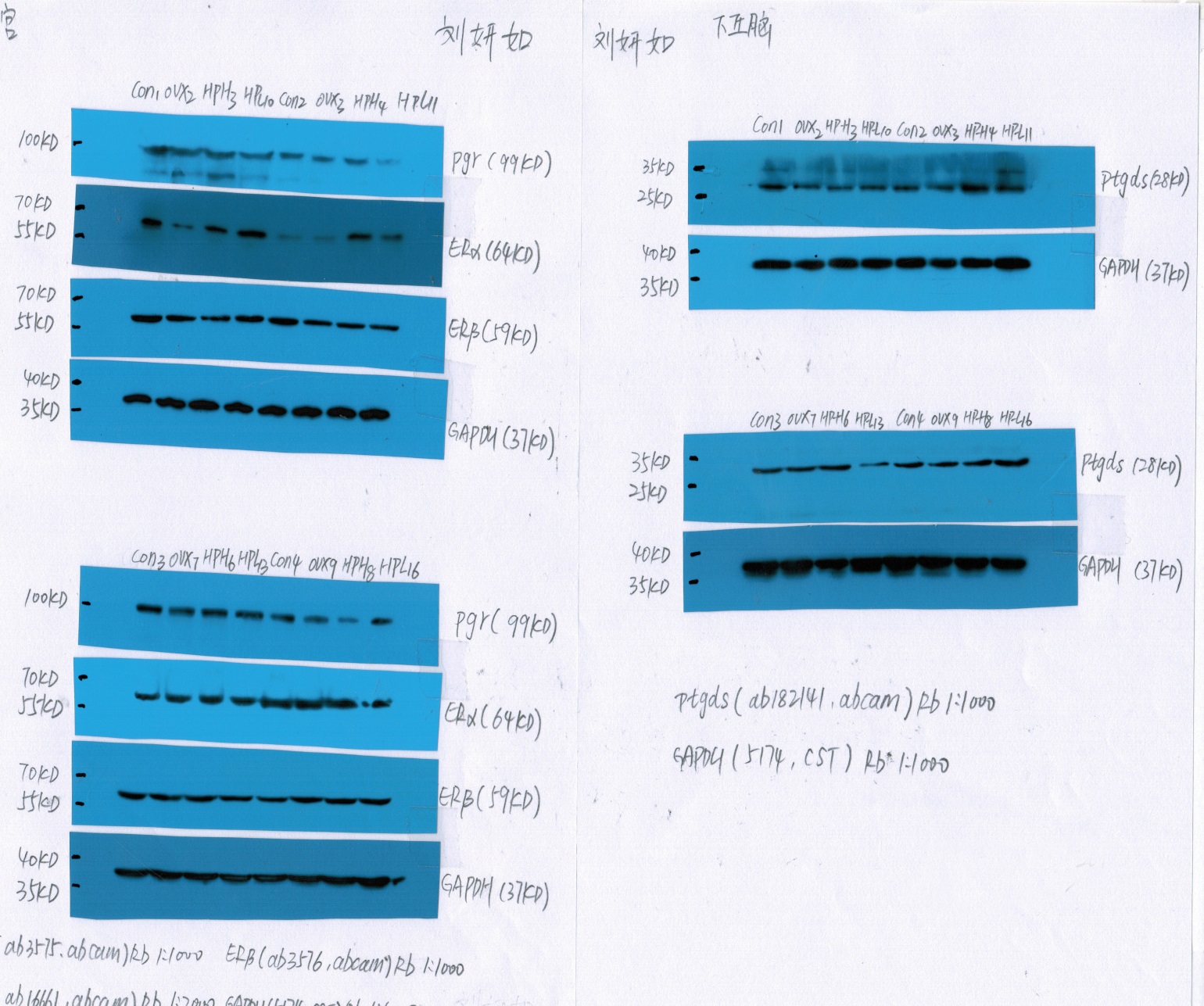

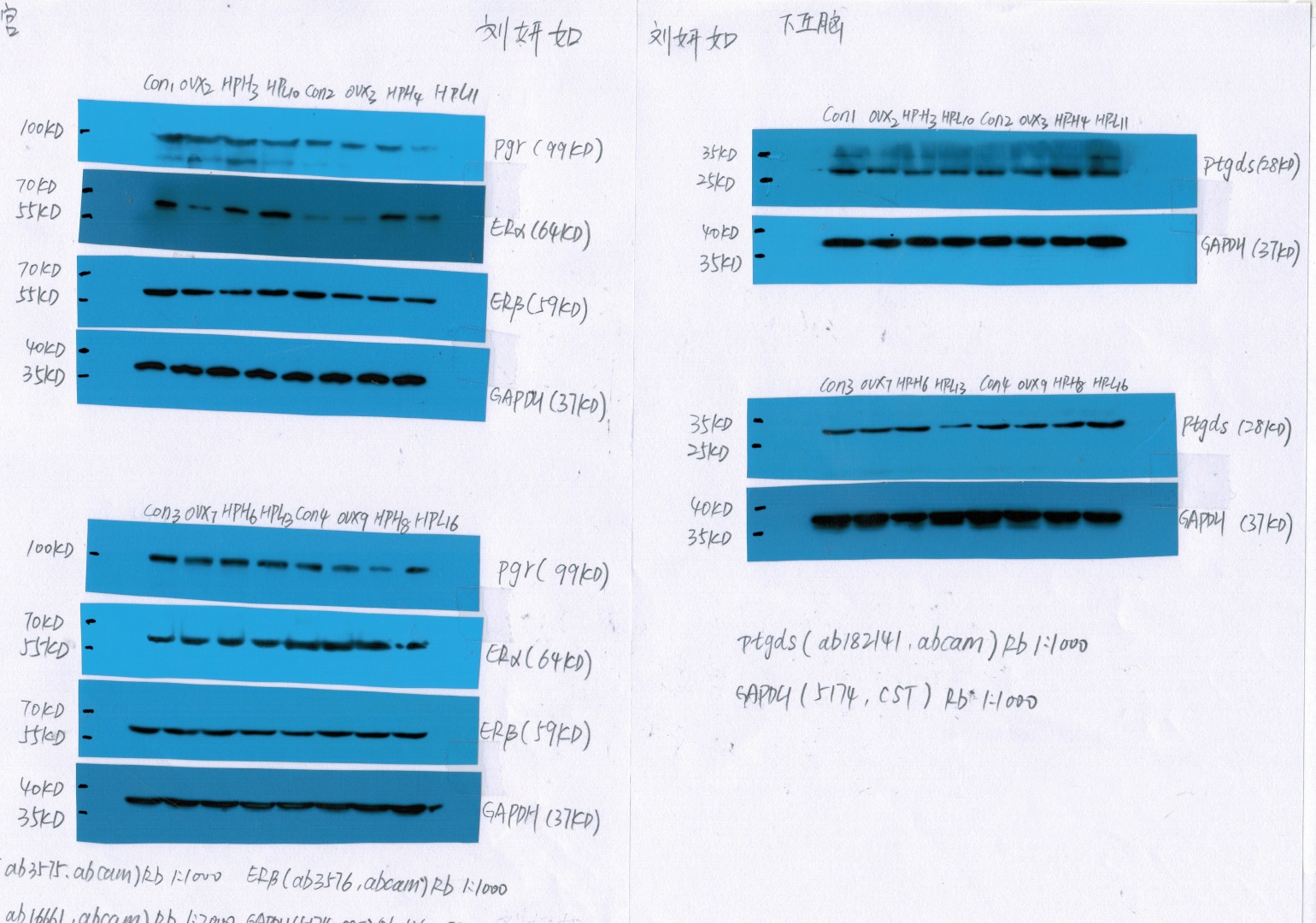


**Figure S1** Western blot original images.
